# Asymmetric Synthesis of Nortropanes *via* Rh-Catalyzed Allylic Arylation

**DOI:** 10.1021/acscatal.2c02259

**Published:** 2022-07-12

**Authors:** Yan Zhang, F. Wieland Goetzke, Kirsten E. Christensen, Stephen P. Fletcher

**Affiliations:** Department of Chemistry, Chemistry Research Laboratory, University of Oxford, 12 Mansfield Road, Oxford OX1 3TA, United Kingdom

**Keywords:** alkaloids, asymmetric catalysis, bicycles, rhodium, Suzuki−Miyaura coupling, kinetic resolution

## Abstract

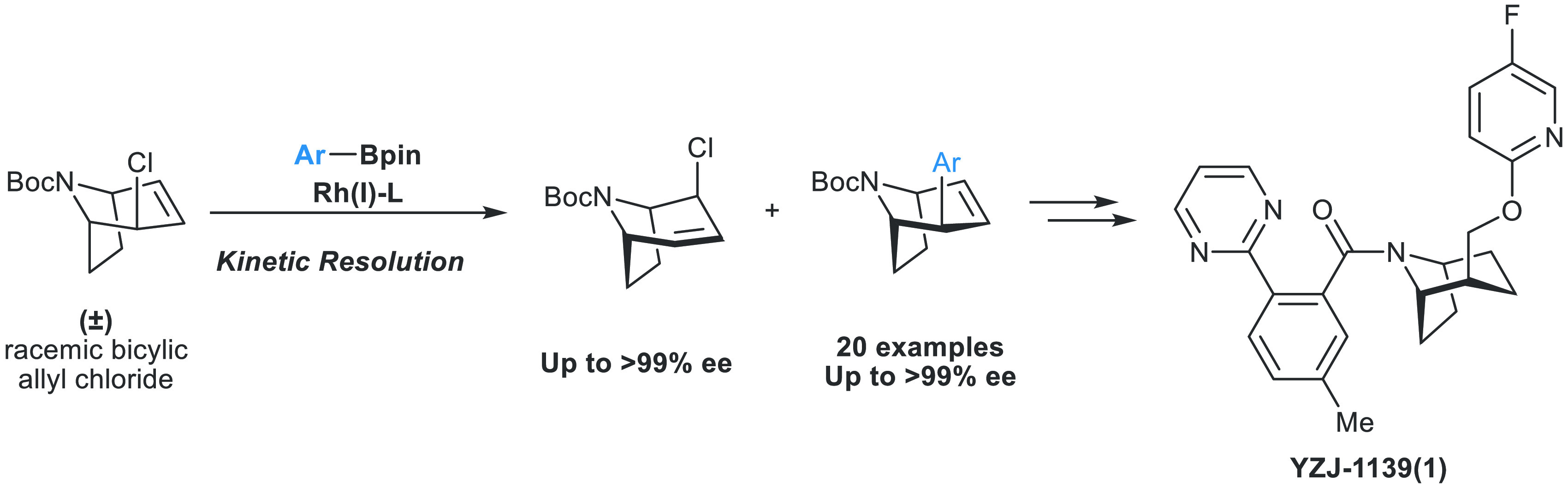

Tropane derivatives are extensively used in medicine, but catalytic asymmetric methods for their synthesis are underexplored. Here, we report Rh-catalyzed asymmetric Suzuki–Miyaura-type cross-coupling reactions between a racemic *N*-Boc-nortropane-derived allylic chloride and (hetero)aryl boronic esters. The reaction proceeds *via* an unexpected kinetic resolution, and the resolved enantiopure allyl chloride can undergo highly enantiospecific reactions with N-, O-, and S-containing nucleophiles. The method was applied in a highly stereoselective formal synthesis of YZJ-1139(1), a potential insomnia treatment that recently completed Phase II clinical trials. Our report represents an asymmetric catalytic method for the synthesis of YZJ-1139(1) and related compounds.

## Introduction

**Scheme 1:**
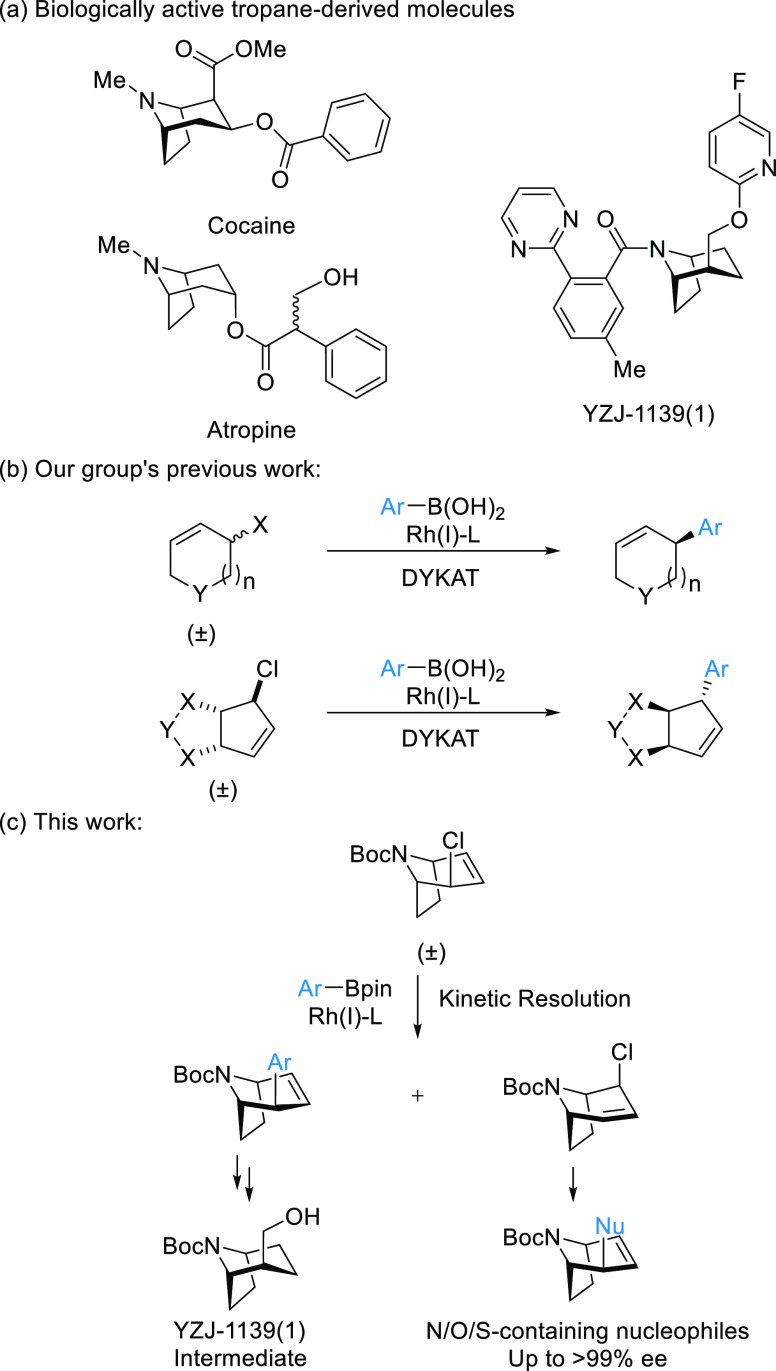
Tropane Derivatives and Works on Rh-Catalyzed Cross-Coupling Reactions of Cyclic Compounds

Molecules with *N*-methyl-8-aza-bicyclo[3.2.1]octane scaffolds, generally known as the tropane alkaloids, display a wide array of biological and pharmaceutical activities.^1−3^ Tropane derivatives, for example, cocaine (Scheme 1a) and scopolamine, are well-known for displaying psychoactive effects, and other tropane derivatives, including atropine (Scheme 1a), are used as anticholinergics and stimulants for treatment of neurological and psychiatric disorders such as Parkinson’s disease and depression.^4−6^ A 8-aza-bicyclo[3.2.1]octane (nortropane)-derived molecule YZJ-1139(1) (Scheme 1a) was also reported recently as an orexin receptor antagonist, which has completed Phase II clinical trials and may become a treatment for insomnia.^7^

Historically, tropane alkaloids had been extracted from plants, but their unique biological activities have inspired the development of synthetic routes to tropane derivatives. Willstätter’s first synthesis of cocaine^8^ and Robinson’s highly efficient double-Mannich approach toward tropinone^9^ are early milestones in this highly active field. Many strategies toward enantioenriched tropanes rely on the derivatization of natural tropane alkaloids, chiral resolution, and synthesis from the chiral pool.^10^

Given the fame of these molecules, and their importance to medicine, it is remarkable that stereoselective methods using achiral starting materials are limited.^3^ Current methods largely use one of two approaches: (1) desymmetrization of *meso* tropinone and its derivatives with stoichiometric chiral lithium amide bases,^11−14^ and (2) enantioselective synthesis of the tropane scaffold where the stereochemical information is introduced concomitant with the formation of the bicycle.^15^ Chiral auxiliary^16−20^ and asymmetric catalytic approaches are known for the latter.^21−27^

Previously, our group reported asymmetric Suzuki–Miyaura-type cross-coupling reactions with racemic mono- and bicyclic allyl chlorides (Scheme 1b).^28−34^ In these highly enantioselective transformations, both enantiomers of the starting material are converted into a single enantiomer of the product. Deracemization is believed to occur *via* the formation of a common *pseudo*-prochiral or *meso* Rh-π-allyl complex (DYKAT type II).^35,36^ We wondered if we could apply a related strategy to the catalytic asymmetric synthesis of sterically congested bicyclic *N*-heterocycles and hence develop an asymmetric cross-coupling approach to the nortropane scaffold (Scheme 1c).

## Results and Discussion

A suitable nortropane-derived allyl chloride (±)-**1a** was synthesized from *N*-Boc-nortropinone in five synthetic steps (see the Supporting Information). Using previously reported conditions for Rh-catalyzed Suzuki–Miyaura cross-couplings established by our group,^31^ the reaction of allyl chloride (±)-**1a** with phenyl boronic acid **2aa** afforded **3a** in 94% enantiomeric excess as a single diastereomer (>20:1), albeit only in 28% yield (Table 1, entry 1).

**Table 1:**
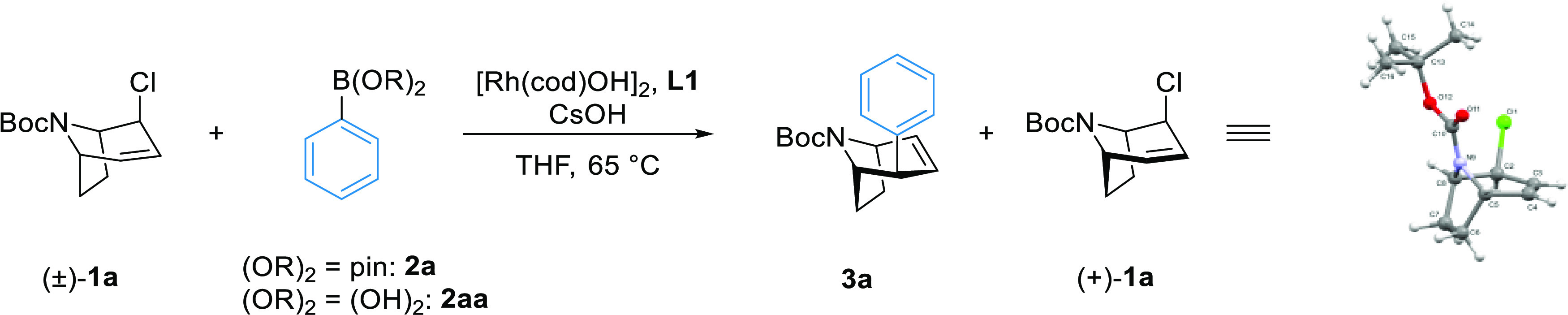

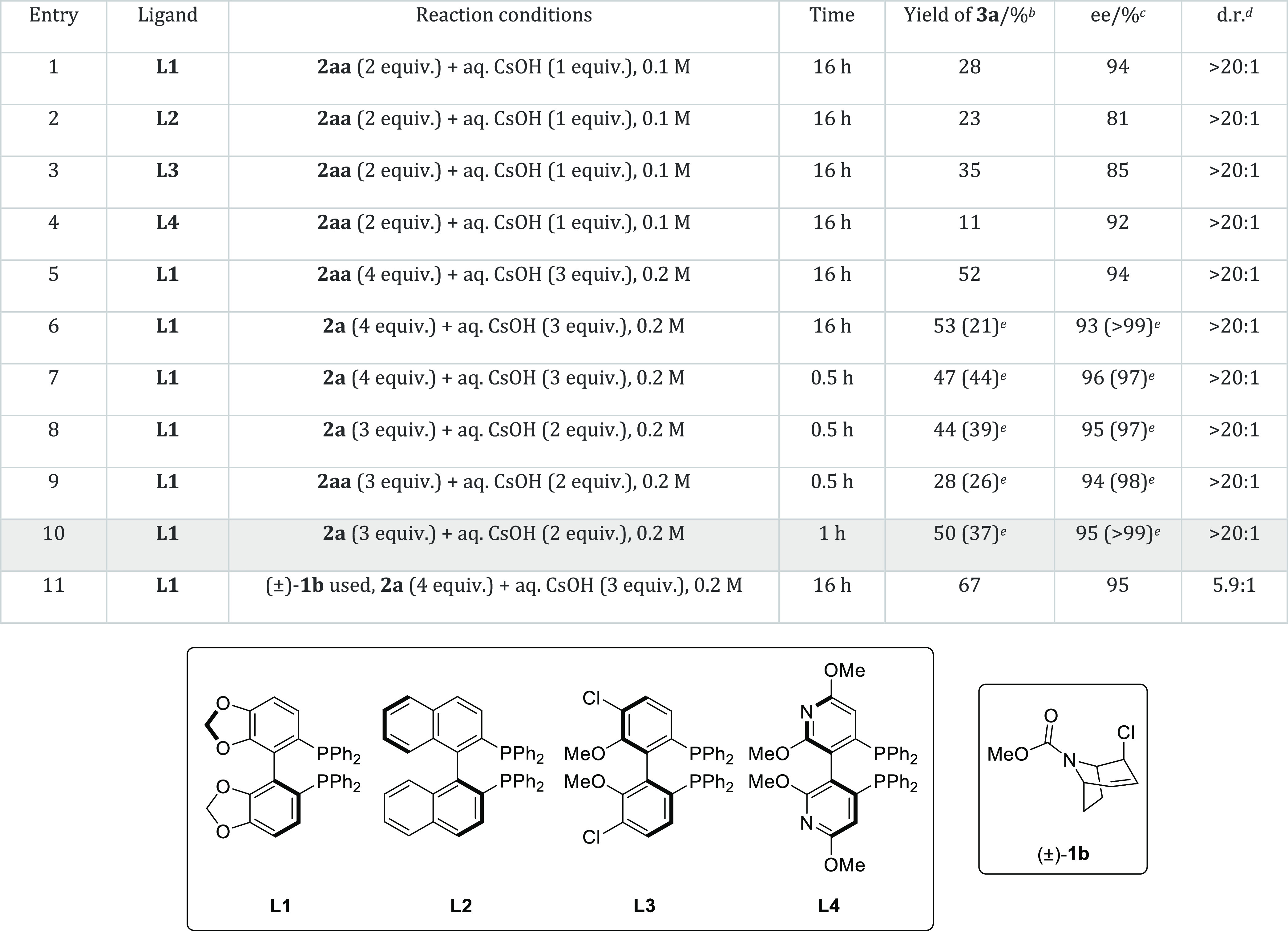
Selected Optimization Experiments^a^

a Rh[(cod)OH]_2_ (2.5 mol %), ligand (6 mol %), (±)-**1a** (0.2 mmol), **2aa** or **2a**, CsOH (50 wt % aq.), THF, 65 °C.

b Isolated yield.

c Enantiomeric excess determined by supercritical fluid chromatography (SFC) analysis on a chiral nonracemic stationary phase.

d Diastereomeric ratio determined by the integration of ^1^H NMR spectra.

e Numbers in brackets refer to the yield and ee of recovered allyl chloride.

We extensively examined the influence of temperature, solvent, base, boronic acid derivatives, catalyst loading, equivalents of reagents, and the use of additives (selected examples are presented in Table 1; for additional data see SI Tables S1 and S2). The protecting group on nitrogen and the leaving group of the nortropinone-derived substrate were investigated. We found that **L1** was superior to related bidentate phosphine ligands regarding both reactivity and enantioselectivity (Table 1, entries 1–4). An increase in yield was observed by increasing both the equivalents of the base and the coupling partner (entry 5). Similar results were obtained using phenyl boronic pinacol ester **2a** as the nucleophile (entry 6), and along with **3a**, we also isolated enantiopure (>99% ee) allyl chloride (+)-**1** in 21% yield.

Upon shortening the reaction time from overnight to 0.5 h, the yields of **3a** were similar, but the yield of (+)-**1a** increased to 37% (entry 7) with a slight decrease in ee (97% ee). We attribute the decreased yield of (+)-**1a** during longer durations to the slower but competitive hydrolysis of **1a** under the reaction conditions. Using less equivalents of boronic pinacol ester and base (entries 7 and 8) gave similar results but purification by column chromatography was easier. Here, using the pinacol ester gave superior results compared to the free boronic acid (entries 8 and 9); at 1 h reaction time, **3a** was isolated in 50% yield (95% ee), and enantiopure (+)-**1a** was isolated in 37% yield (entry 10).

Changing the protecting group on nitrogen from *N-*Boc to methyl carbamate resulted in a higher yield at 63% and ee of 93% (entry 11). However, the diastereoselectivity decreased drastically from >20:1 to 5.9:1, likely due to steric reasons.

**Scheme 2:**
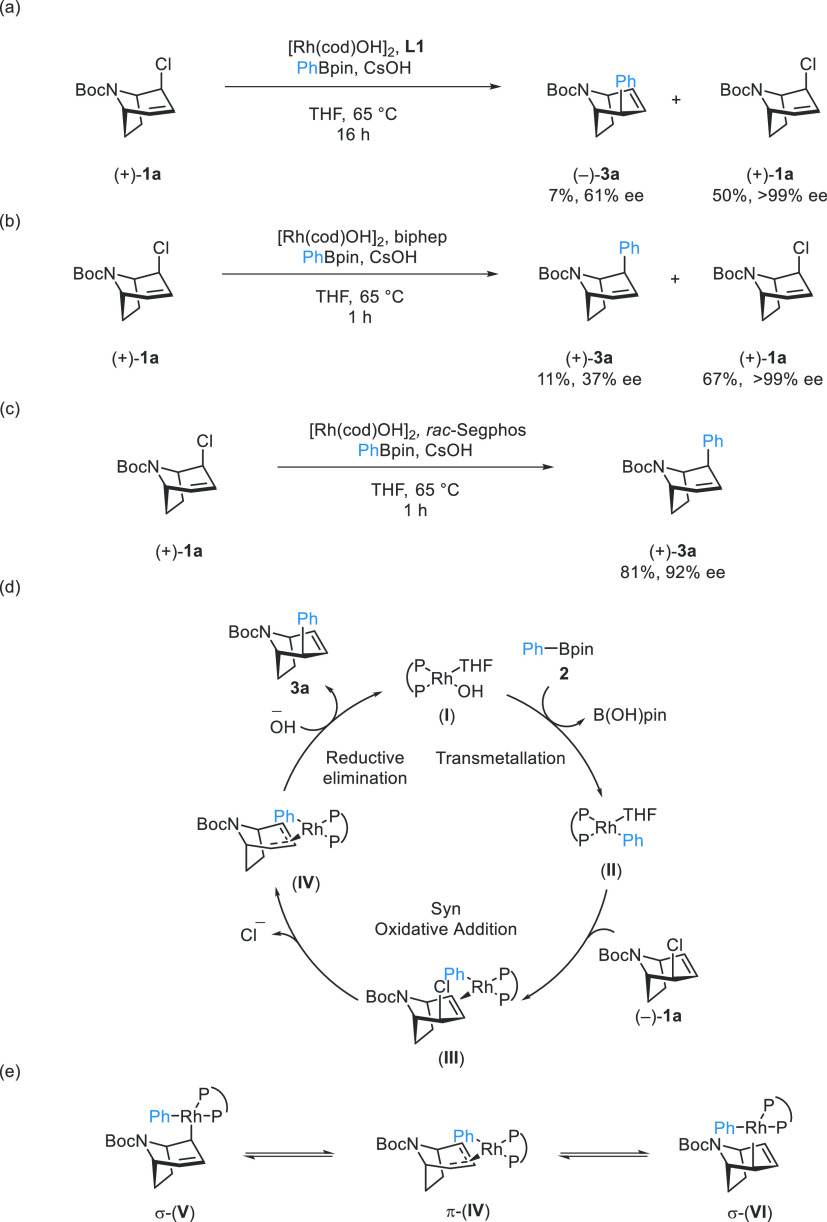
Mechanistic Investigations and the Proposed Mechanism^,^ Rh-catalyzed Suzuki–Miyaura coupling reaction with (+)-**1a** and **2a**. Rh-catalyzed Suzuki–Miyaura coupling reaction with (+)-**1a** and biphep as ligand. Rh-catalyzed Suzuki–Miyaura coupling reaction with (+)-**1a** and *rac*-Segphos as ligand. Proposed mechanism. Equilibration between two Rh-σ complexes.

A highly selective kinetic resolution would intrinsically limit the obtained yields of these reactions to 50%, but we also speculated that catalyst deactivation or decomposition of the boronic ester could limit conversion. To help distinguish between these scenarios, we subjected (+)-**1** (>99% ee) instead of racemic allyl chloride to our standard reaction conditions and allowed the reaction to occur overnight (Scheme 2a). Small amounts of the desired coupling product were obtained (7%), albeit to our surprise only with 61% ee. Additionally, some enantiopure allyl chloride (+)-**1a** (50%) was recovered, while the rest of the substrate was hydrolyzed to the corresponding allyl alcohol.

This result indicates that, unlike the previous DYKAT process developed by our group, where both the enantiomers form a common symmetric Rh-π-allyl complex intermediate, when using **L1**, oxidative addition of (+)-**1a** either does not occur or does not give the same intermediate as enantiomer (−)-**1a** (Scheme 2d), and product formation *via* (+)-**1a** is slow.

To test if oxidative addition (−)-**1a** would result in the formation of *meso* π-complex, which would result in complete loss in stereochemical information upon oxidative addition, we performed a cross-coupling reaction with enantiopure allyl chloride (+)-**1a** with an achiral ligand biphep (Scheme 2b). Under these conditions, we obtained (+)-**3a** in 11 and 37% ee. The significant loss in ee in the reaction with biphep suggests that reductive elimination is at least partially enantio-determining and is controlled by the ligand when Segphos is used. We attribute the partial, but not complete, loss in stereochemical information in the experiment with biphep to either σ–π–σ isomerization mechanism (Scheme 2e), which occurs at a rate similar to reductive elimination, or to a lack of selectivity amongst different oxidative addition-type pathways when biphep is used.

Using a racemic mixture of Segphos as the ligand in the reaction with enantiopure (+)-**1a**, we obtained (+)-**3a** in 81% yield and 92% ee (Scheme 2c). In analogy to the combination of racemic allyl chloride and enantiopure ligand, this experiment shows the strong matched effect between (+)-**1a** and (*R*)-Segphos in the oxidative addition step (or (−)-**1a** and (*S*)-Segphos), most likely for steric reasons, which ultimately results in a highly selective kinetic resolution.

The partial DKYAT character of this transformation (Scheme 2a,e) does not allow for a meaningful quantification of *s*-factors (see SI*p*. S36).

**Scheme 3:**
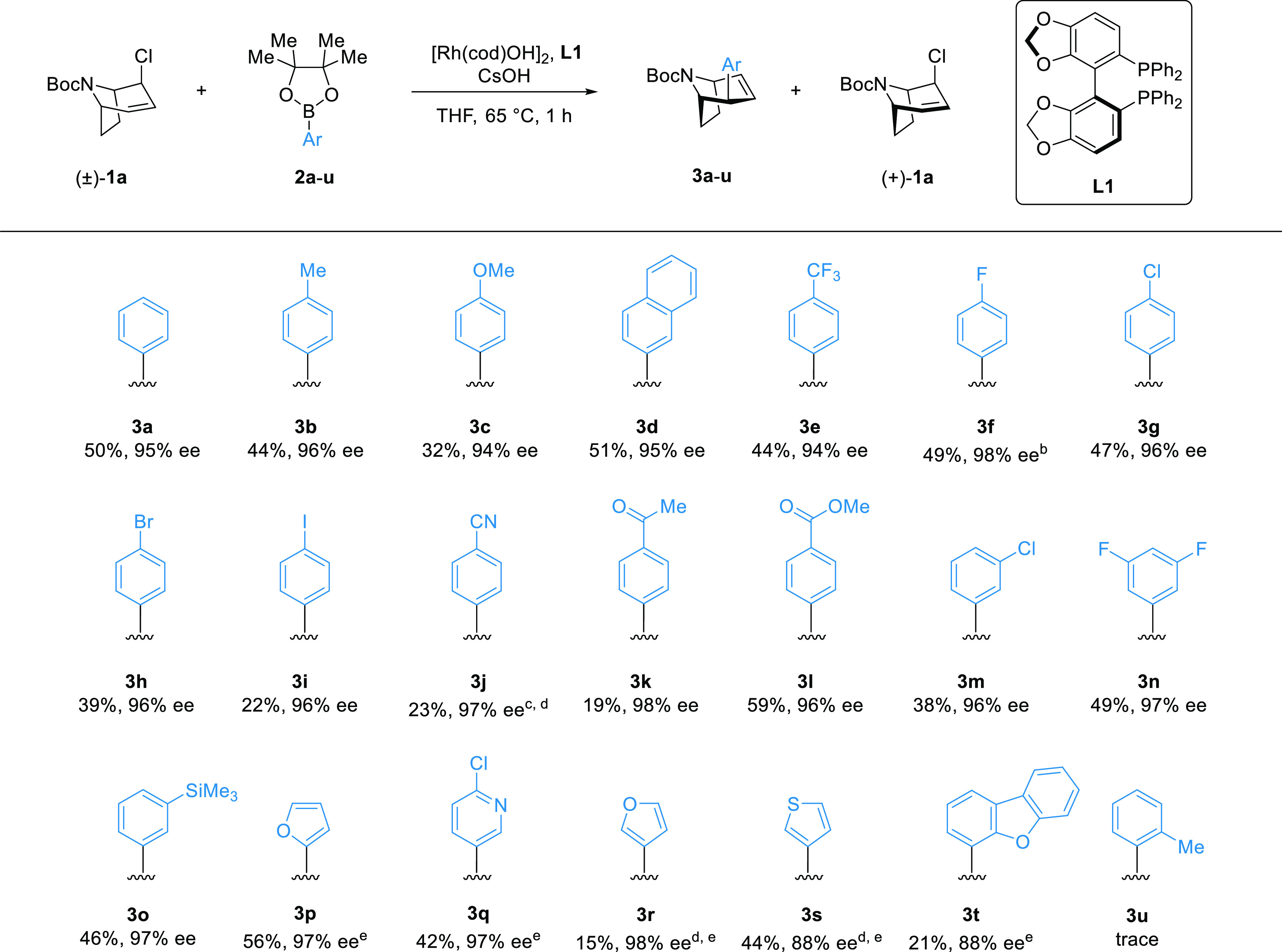
Scope of the Reaction Rh[(cod)OH]_2_ (2.5 mol %), **L1** (6 mol %), (±)-**1a** (0.2 mmol, 1.0 equiv), **2** (3.0 equiv), CsOH (50 wt % aq.; 2.0 equiv), THF (0.2 M), 65 °C, 1 h. All yields presented are isolated yields. Enantiomeric excess determined by supercritical fluid chromatography (SFC) analysis on a chiral nonracemic stationary phase. Single diastereomer (>20:1) obtained unless stated. 4 h. Dioxane instead of THF, 80 °C overnight. **3j**: 18:1 d.r., **3r**: 17:1 d.r., **3s**: 19:1 d.r. 2 h.

A range of aryl boronic pinacol esters with electron-withdrawing and donating substituents at the *para*- and *meta*-positions yielded the desired coupling products typically in 40–50% yield (Scheme 3, **3a**–**3h**, **3l**–**3o**) and >94% ee as single diastereomers (>20:1 d.r.). These examples included various aryl halides, alkoxy groups, ester, and an aryl silane, which are useful intermediates for further reactions.

More challenging coupling partners featuring an iodide, cyano, or acetyl group (**3i**–**3k**) resulting in diminished yields but consistently high enantioselectivity.

We observed only trace product formation with 2-methylphenylboronic pinacol ester (**2u**), likely due to sterics and/or competitive protodeborylation.^37^

Heteroaryl boronic pinacol esters can be used. 2-Furanyl- and 2-chloropyridyl-boronic pinacol esters performed well with yields over 40% and high enantioselectivities (**3p**, **3q**). 3-Furanylboronic pinacol esters gave only 15% yield, likely due to rapid protodeborylation—a common problem with heterocyclic boronic acids and esters.^38^

Interestingly, a few examples gave >50% yield (**3l** and **3p** gave 59 and 56% yield, respectively), which we have briefly investigated but still do not fully understand, and the yield of **3p** was found to further improve upon scale-up (see below).

**Scheme 4:**
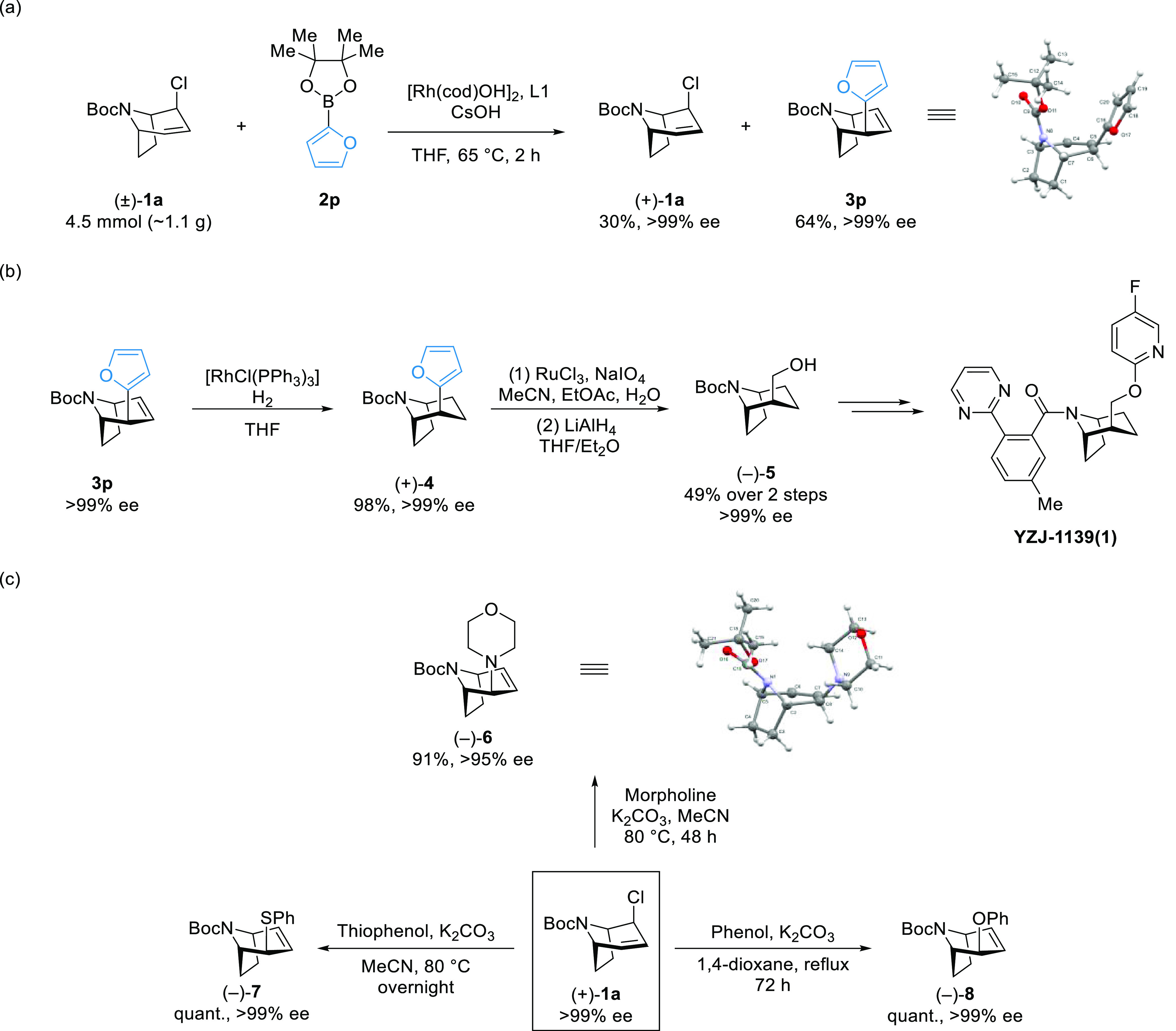
Scale-Up of the Cross-Coupling Reaction with 2p and Transformation of the Coupling Product and Enantioenriched Substrate^,^ Reaction at 1 g scale with 2-furanylboronic pinacol ester **2p**. Formal synthesis of YZJ-1139(1). Enantiospecific transformation of enantiopure allyl chloride (+)-**1a**.

The absolute and relative stereochemistries of the product and resolved allyl chloride (Schemes 3a and 4a) were determined by single-crystal X-ray diffraction of **3p** and (+)-**1a**.^39,40^

To demonstrate the synthetic utility of our method, we applied Rh-catalyzed Suzuki–Miyaura coupling with allyl chloride (±)-**1a** to formal synthesis of the orexin receptor antagonist YZJ-1129(1) (Scheme 4a,b),^41^ which recently passed Phase II clinical trials for the treatment of sleep disorders. To the best of our knowledge, the only other previously reported synthesis of YZJ-1129(1) was recently reported by a process chemistry group and relied on preparative high-performance liquid chromatography (HPLC) separation of the enantiomers or a chiral auxiliary approach.^7^

A gram-scale cross-coupling reaction between (±)-**1a** and 2-furanylboronic pinacol ester **2p** afforded **3p** in 64% isolated yield and >99% ee (Scheme 4a). The enantioenriched allyl chloride (+)-**1a** was isolated in 30% yield and >99% ee.

Reduction of **3p** with Wilkinson’s catalyst gave (+)-**4** in 98% yield and >99% ee. The furyl group was then converted into a hydroxymethyl group *via* a two-step oxidative cleavage/reduction protocol to give (−)-**5** in 49% yield, a previously reported intermediate in the synthesis of YZJ-1129(1).

Previously, our group reported a Cu-catalyzed kinetic resolution reaction of a piperidine-derived allyl chloride, and the enantioenriched allyl chloride was used in enantiospecific substitution reactions with heteroatom-based nucleophiles,^42^ and we wondered if related substitutions with (+)-**1a** were possible.

The substitution with morpholine, thiophenol, and phenol gave (−)-**6**, (−)-**7**, and (−)-**8** in excellent yield and enantiospecificity (Scheme 4c), respectively. Other enantiospecific substitution reactions with this substrate are likely possible. The absolute stereochemistry of (−)-**6** was determined by single-crystal X-ray diffraction, and this, combined with the knowledge of the absolute configuration of the starting material, indicates that the substitution reaction proceeds *via* an syn-S_N_2′ pathway (Scheme 4c) The relative and absolute stereochemistry of (−)-**7** and (−)-**8** is assigned in analogy to (−)-**6**.

## Conclusions

In summary, we developed an efficient kinetic resolution of a nortropane-derived allyl chloride *via* Rh(I)-catalyzed Suzuki–Miyaura cross-couplings. The reaction tolerates a range of different aryl- and heteroaryl boronic pinacol esters with synthetically useful functional groups in high enantioselectivity and diastereoselectivity. The coupling product with 2-furanyl pinacol ester was used in a formal asymmetric synthesis of YZJ-1129(1). Further, the resolved enantiopure allyl chloride can undergo enantiospecific syn-S_N_2′ reactions with *O*-, *S*-, and *N*-nucleophiles. Overall, this work provides access to a wide range of enantiomerically enriched tropane derivatives.

## Supporting Information Available

Experimental procedure; characterization of substrates and products (NMR, IR, HRMS, optical rotation); ^1^H, ^13^C, and ^19^F NMR spectra; SFC traces and X-ray data (PDF)
Crystallographic data for (+)-**1a**, **3p**, and (−)-**6** have been deposited at the Cambridge Crystallographic Data Centre as supplementary publication nos. CCDC 2156812, 2156553, and 2156805 and can be obtained *via*www.ccdc.cam.ac.uk/data_request/cif (CIF)
Checkcif.pdf (PDF)

## Supplementary Material

cs2c02259_si_001.pdf

cs2c02259_si_002.cif

## Notes

Oxford University Innovation has filed a patent application (PCT/GB2016/051612) with S.P.F. named as an inventor. Y.Z. F.W.G., and K.E.C. declare no competing financial interests.

## Dedication

Dedicated to the memory of Stephen A. Westcott.
