# Supplementary material for: Asymmetric Synthesis of Nortropanes via Rh-Catalyzed Allylic Arylation
Source: ACS Catal. 2022 Jul 12;12(15):8995–9002. doi: 10.1021/acscatal.2c02259 (PMC9361292; doi:10.1021/acscatal.2c02259)
Supplement: Supplementary file 1 — cs2c02259_si_001.pdf [file cs2c02259_si_001.pdf]

Supporting Information

Asymmetric synthesis of nortropanes *via* Rh-catalyzed allylic arylation

Yan Zhang,[a] F. Wieland Goetzke,[a] Kirsten E. Christensen,[a] and Stephen P. Fletcher *[a]

[a] Department of Chemistry, Chemistry Research Laboratory, University of Oxford, 12 Mansfield Road, Oxford, OX1 3TA (UK) * stephen.fletcher@chem.ox.ac.uk

Table of Contents

1. Experimental procedures S3-36

1.1 General Information S3

1.2 Synthesis of the starting materials S4-8

1.3 Rh-catalyzed Suzuki-Miyaura Reactions S9-26

1.3.1 General Procedure A S9

1.3.2 Reaction Optimization S9-11

1.3.3 Product Charaterizations S12-30

1.4 Upscale and derivatization of products S31-35

1.5 Calculation of Apparent *S*-factors..... S36

2. References S37

3. NMR spectra S38-69

4. SFC traces S70-97

5. X-ray crystallographic analysis S98-101

6. Author Contributions S101

1. Experimental Procedures

1.1 General Information

All oxygen and moisture sensitive reactions were performed in flame-dried flasks under argon atmosphere with standard Schlenk techniques. Magnetic stirring was present throughout all reactions. All reactions performed with heating is performed utilizing DrySyn heating blocks. All the reactions at 0 ℃ were performed in ice-water cooling bath with cotton wool covering.

Nuclear Magnetic Resonance spectroscopy (^{1}H, ^{13}C, ^{19}F, COSY, HSQC, NOESY) was measured at room temperature in deuterated solvent (Sigma Aldrich) using Bruker AVIII HD 400 (400 MHz for ^{1}H NMR, 101 MHz for ^{13}C NMR, 376 MHz for ^{19}F NMR) or Bruker NOE600 (600/151 MHz) spectrometers. Chemical shifts were reported in parts per million (ppm) relative to residual solvent peak. Assignments were made in accordance with COSY and HSQC spectra.

Infrared spectroscopy was carried out on a Bruker Tensor 27 FT-IR spectrometer with internal calibration range of 4000-600 cm^{-1}.

Optical rotations ($[\alpha]^{25}_{D}$) were recorded using a Schmidt Haensch Unipol L2000 Polarimeter in a cell with a path length of 1 dm (using the sodium D line, 589 nm). Concentrations (c) are reported in g/100 mL. Temperatures are reported in °C.

Chiral SFC separation was conducted on Waters Acquity UPC2 system in Waters Empower software. Chiralpak columns (150 × 3 mm, particle size 3 μm) used for separation were specified in individual reactions. HPLC grade solvent was used (Sigma Aldrich/Fisher Scientific).

High Resolution Mass spectra were carried out by internal service at the University of Oxford. Electron spray ionisation (ESI^{+}) was recorded on a Fisons Platform II. Analyses were performed using a Thermo Exactive mass spectrometer equipped with Waters Acquity liquid chromatography system.

Commercially available reagents were purchased from: Sigma Aldrich, Alfa Aesar, Acros Organics, Fluorochem and Strem Chemical, they were used without further purifications unless otherwise stated. The ligands screened in catalytic reactions were either purchased or synthesized by group members. All the boronic pinacol esters were synthesized from the corresponding boronic acid and purified *via* column chromatography.

Anhydrous solvent was collected freshly from mBraun SPS-800 solvent purification system after passed through anhydrous alumina columns. Anhydrous THF for all Rh-catalyzed reactions was purchased from Acros under molecular sieves.

Medium-pressure chromatography was performed on a CombiFlash Next Gen 100 system using a RediSep Gold® column. Manual Flash column chromatography was carried out using Merck 60 Å silica gel and VWR (40-63 μm) silica. Pressure was applied at the column head *via* a flow of nitrogen with the solvent system used in parentheses.

1.2 Synthesis of starting materials

Synthesis of Nortropane-derived allyl chloride (±)-1a

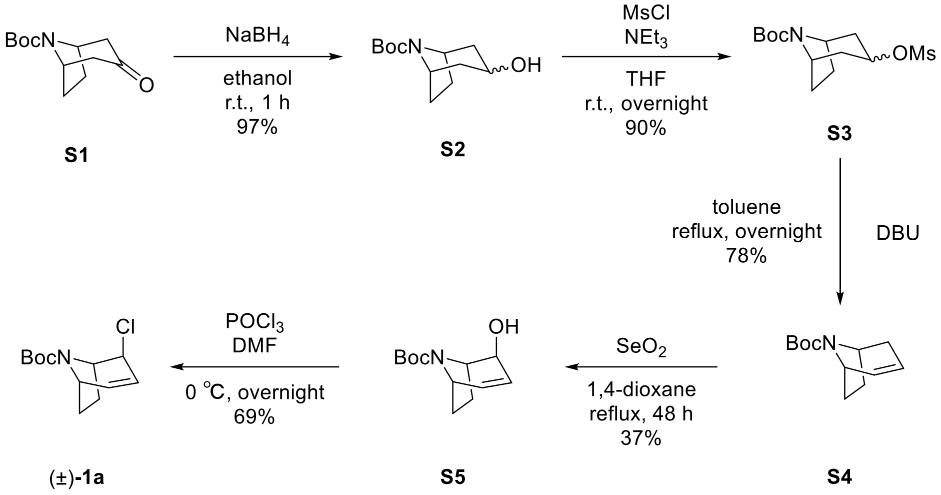

Scheme S1 Overview of synthesis of allyl chloride (±)-**1a**

Synthesis of S2

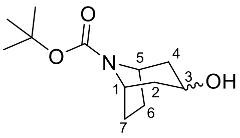

S2

Sodium borohydride (1.68 g, 44.4 mmol) was added portionwise to a solution of **S1** (5.00 g, 22.2 mmol) in ethanol (100 mL) at 0 °C, then the reaction mixture was allowed to warm up to r. t.. After stirring for 1h, NH_4Cl solution (sat. aq., 50 mL) followed by H_2O (50 mL) were added. The aqueous layer was extracted with EtOAc (100 mL × 3), and the combined organic layer was dried over $MgSO_4$. The resulting solution was concentrated in *vacuo* to afford **S2** as a mixture of diastereomers (d.r. ~ 1.1:1). **S2** was used directly in the next step without purification (4.85 g, 97% yield). The spectroscopic data are in agreement with the literature.[1]

^{1}H NMR ($CDCl_3$, 400 MHz, both diastereomers): δ (ppm) = 4.26-4.06 (m, 3H, C_1-H, C_3-H, C_5-H), 2.25-1.81 (m, 4H, C_2-H, C_4-H), 1.77-1.48 (m, 4H, C_6-H, C_7-H), 1.46/1.45 (2 × s, 9H, -C(CH_3)$_3$).

^{13}C NMR ($CDCl_3$, 101 MHz, both diastereomers): δ (ppm) = 153.6 (-C=O), 153.4 (-C=O), 79.5 (-$\underline{C}(CH_3)_3$), 79.2 (-$\underline{C}(CH_3)_3$), 65.5 (C_3), 64.2 (C_3), 53.3 (C_1/C_5), 52.8 (C_1/C_5), 40.7 (C_2/C_4), 38.6 (C_2/C_4), 28.66 (-$C(\underline{C}H_3)_3$), 28.65 (-$C(\underline{C}H_3)_3$), 28.1 (C_6, C_7).

HRMS (APCI): m/z calculated for $C_{12}H_{21}O_3NNa^+$ $[M+Na]^+$ 250.1414, found 250.1414.

Synthesis of S3

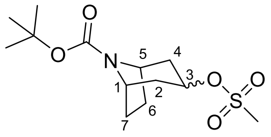

S3

According to a literature procedure by Nagase *et. al.*,[2] triethylamine (4.7 mL, 36.8 mmol) was added to a solution of **S2** (5.57 g, 24.5 mmol) in tetrahydrofuran (50 mL) under an argon atmosphere. Methanesulfonyl chloride (2.1 mL, 29.6 mmol) was added dropwise at 0 °C. The reaction mixture was allowed to reach r. t. and stirred overnight. Then, H_2O (50 mL) was added, and the resulting mixture was extracted with EtOAc (50 mL × 3) and the combined organic layer was dried over $MgSO_4$. The solution was concentrated *in vacuo* and the crude product was purified by flash chromatography (hex/EtOAc=50/50) to afford **S3** (d.r. ~ 4.3:1, 64% yield) with recovered **S2** as a light yellow oil. The mixture of **S2** and **S3** was dissolved in THF (20 mL), followed by the addition of triethylamine (1.7 mL, 13.2 mmol) and methanesulfonyl chloride (0.76 mL, 8.82 mmol) were added dropwise at 0 °C. The reaction mixture was allowed to reach r. t. and stirred overnight. Then, H_2O (20 mL) was added, and the resulting mixture was extracted with EtOAc (20 mL x 3) and the combined organic layer was dried over $MgSO_4$. The solution was concentrated *in vacuo* and the crude product was purified by flash chromatography (hex/EtOAc=50/50) to afford **S3** (d.r. ~ 1.4:1, 90% yield) The spectroscopic data is in agreement with the literature.[2]

^{1}H NMR ($CDCl_3$, 400 MHz, major diastereomer): δ (ppm) = 4.98 (tt, *J* = 10.9, 6.2 Hz, 1H, C_3-H), 4.35-4.07 (m, 2H, C_1-H, C_5-H), 2.94 (s, 3H, -CH_3), 2.20-1.88 (m, 4H, C_2-H, C_4-H), 1.88-1.68 (m, 2H, C_6-H/C_7-H), 1.67-1.51 (m, 2H, C_6-H/C_7-H), 1.47 (s, 9H, -$C(CH_3)_3$), 1.45 (s, -$C(CH_3)_3$)

^{13}C NMR ($CDCl_3$, 101 MHz, major diastereomer): δ (ppm) = 153.2 (-C=O), 80.0 (-$\underline{C}(CH_3)_3$), 75.1 (C_3), 52.6 (C_1, C_5), 38.9 (CH_3), 38.5 (C_2/C_4, C_6/C_7), 38.0 (C_2/C_4, C_6/C_7), 37.2 (C_2/C_4, C_6/C_7), 28.4 (-$C(\underline{C}H_3)_3$), 27.5 (C_2/C_4, C_6/C_7)

HRMS (APCI): m/z calculated for $C_{13}H_{23}O_5NSNa$+ $[M+Na]^+$ 328.1189, found 328.1188.

Synthesis of S4

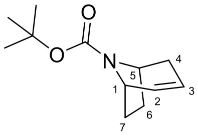

S4

According to a literature procedure by Dallanoce and coworkers,[3] DBU (4.0 mL, 27 mmol) was added to a solution of **S3** (2.91g, 9.13 mmol) in toluene (11 mL), and the reaction mixture was stirred under reflux overnight. The reaction mixture was filtered over Celite® and concentrated *in vacuo*. Flash column chromatography (hex/EtOAc=85/15) afforded **S4** as a light yellow oil (1.56 g, 78% yield). The spectroscopic data is in agreement with the literature.[4]

^{1}H NMR ($CDCl_3$, 400 MHz): δ (ppm) = 5.91 (s, 1H, C_2-H), 5.44 (dddd, *J* = 9.6, 4.1, 2.4, 1.3 Hz, 1H, C_3-H), 4.45-4.02 (m, 2H, C_1-H, C_5-H), 2.68 (s, 1H, C_4-H). 2.08 (m, 1H, C_6-H/C_7-H), 1.84 (tdd, *J* = 11.8, 10.2, 6.6 Hz, 2H, C_6-H/C_7-H), 1.71 (dd, *J* = 17.6, 4.4 Hz, 1H, C_4-H), 1.61 (m, 1H, C_6-H/C_7-H), 1.38 (s, 9H, -$C(CH_3)_3$).

^{13}C NMR ($CDCl_3$, 101 MHz): δ (ppm) = 154.1 (-C=O), 132.9 (C_2), 123.8 (C_3), 79.1 (-<u>C</u>(CH_3)$_3$), 52.8 (C_1, C_5), 34.7 (C_4, C_6/C_7), 29.9 (C_6/C_7), 28.4 (-C(<u>C</u>H_3)$_3$).

HRMS (APCI): m/z calculated for $C_{12}H_{19}O_2NNa^+$ $[M +Na]^+$ 232.1308, found 232.1309.

Synthesis of (±)-S5

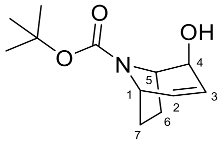

(±)-**S5**

According to a related literature procedure by Chavan *et. al.*,[5] SeO_2 (0.550 g, 4.96 mmol) was added to a solution of **S4** (0.944 g, 4.51mmol) in 1,4-dioxane (270 mL). The reaction mixture was stirred under reflux for 48 h. The reaction mixture was then cooled to r.t., filtered through Celite® and concentrated *in vacuo*. The crude product was dissolved in EtOAc (30 mL) and washed with $NaHCO_3$ (sat. aq., 20 mL); the aqueous layer was extracted with EtOAc (20 mL × 3), and the combined organic layer was washed with brine (20 mL) and dried over $MgSO_4$. The solution was concentrated *in vacuo* and the crude product was purified by flash column chromatography (hex/EtOAc= 70/30) to afford (±)-**S5** as a yellow oil (0.399 g, 37 % yield) as a single diastereomer (d.r. > 20:1).

^{1}H NMR ($CDCl_3$, 400 MHz): δ (ppm) = 6.12 (dd, J = 9.6, 5.3 Hz, 1H, C_2-H), 5.78 – 5.59 (m, 1H, C_3-H), 4.46 (app. d, J = 8.2 Hz, 2H, C_1-H, C_5-H), 3.72 (dd, J = 9.7, 4.1 Hz, 1H, C_4-H), 3.27 (br. s, 1H, -OH), 2.23 – 2.07 (m, 1H, C_6/C_7-H), 1.98 – 1.72 (m, 1H, C_6/C_7-H), 1.72 – 1.54 (m, 2H, C_6/C_7-H), 1.46 (s, 9H, - C(CH_3)$_3$), 1.33 – 1.19 (m, 1H, C_6/C_7-H).

^{13}C NMR ($CDCl_3$, 101 MHz): δ (ppm) = 170.6 (-C=O), 133.7 (C_2), 126.2 (C_3), 79.6 (-<u>C</u>(CH_3)$_3$), 71.1 (C_4), 58.0 (C_1/C_5), 53.5 (C_1/C_5), 30.2 (C_6/C_7), 28.1 (-C(<u>C</u>H_3)$_3$), 24.3 (C_6/C_7).

HRMS (APCI): m/z calculated for $C_{12}H_{19}O_3NNa^+$ $[M+Na]^+$ 248.1257, found 248.1258.

IR: 3431, 2975, 1670, 1478, 1393, 1366, 1341, 1298, 1251, 1160, 11106, 1057, 1023, 957, 894, 868, 849, 831, 774, 718 cm^{-1}

Synthesis of (±)-1a

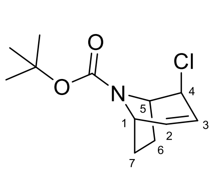

(±)-**1a**

According to a related literature procedure by Schäfer *et al.*,[6] (±)-**S5** (0.492 g, 2.18 mmol) was dissolved in DMF (3.5 mL) at 0 °C and cold $POCl_3$ (0.45 mL) in DMF (3.5 mL) was added dropwise. The reaction was allowed to reach r. t. overnight. Then, NaOH solution (1M in H_2O, 2.5 mL) was added, and the mixture was extracted with EtOAc (5 mL). The combined organic layer was dried over $MgSO_4$ and concentrated *in vacuo*. The crude product was purified *via* flash column chromatography (hex/EtOAc=85/15) to afford (±)-**1a** as a colourless oil, which solidified at –20°C (0.368 g, 69% yield) to give an off white solid. The product was afforded as a single diastereomer (d.r. > 20:1).

The relative stereochemistry was assigned *via* X-ray diffraction.

^{1}H NMR ($CDCl_3$, 400 MHz): δ (ppm) = 6.13 (m, 1H, C_2-H), 5.65 (d, , *J* = 9.5 Hz, 1H, C_3-H), 4.57 (m, 2H, C_1-H, C_5-H), 4.23 (s, 1H, C_4-H), 2.29-2.06 (m, 1H, C_6/C_7-H), 1.85 (s, 1H, C_6/C_7-H), 1.77-1.65 (m, 1H, C_6/C_7-H), 1.60-1.52 (m, 1H, C_6/C_7-H), 1.47 (s, 9H, -$C(CH_3)_3$)

^{13}C NMR ($CDCl_3$, 101 MHz): δ (ppm) = 153.1 (-C=O), 134.9 (C_2), 124.6 (C_3), 80.0 (-$\underline{C}(CH_3)_3$), 59.1 (C_4), 58.7 (C_1/C_5), 58.1 (rot. C_1/C_5), 53.4 (rot. C_1/C_5), 51.7 (C_1/C_5), 31.6 (C_6/C_7), 28.5 (-$C(\underline{C}H_3)_3$), 27.5 (C_6/C_7).

HRMS (APCI): m/z calculated for $C_{12}H_{18}ClO_2NNa^+$ $[M+Na]^+$ 266.0918, found 266.0919.

IR: 2975, 2929, 2883, 1677, 1629, 1475, 1422, 1389, 1366, 1344, 1317, 1297, 1246, 1224, 1160, 1104, 1040, 1009, 969, 906, 891, 869 cm^{-1}

Melting Point: 55 - 59 °C

Synthesis of (±)-1b

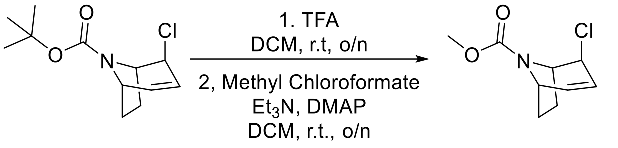

Scheme S2 Overview of synthesis of allyl chloride (±)-**1b**

According to related procedure by Karabiyikoglu *et al.*,[7] (±)-**1a** (0.546g, 2.24 mmol) was dissolved in DCM (17 mL) at 0 °C and TFA (1.8 mL, 22.2 mmol) was added dropwise. The ice bath as removed and the reaction mixture was left stirring overnight. The crude mixture was concentrated *in vacuo*. DMAP (54.5 mg, 0.45 mmol) was added, followed by DCM (7.2 mL). Triethylamine (1.9 mL, 13.4 mmol) and methyl chloroformate (0.48 mL, 3.4 mmol) were added dropwise at 0 °C. The reaction mixture was allowed to reach r.t. overnight. DCM (60 mL) was added, and the organic layer was washed with $NaHCO_3$ (sat. aq. solution, 60 mL), brine (60 mL) and dried over $MgSO_4$. The reaction mixture was concentrated *in vacuo* and purified by flash column chromatography (hex/EtOAc = 90/10 to 80/20) to afford (±)-**1b** as a colourless oil (0.45 g, quant. yield).

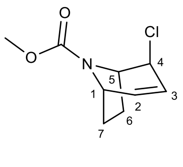

(±)-**1b**

^{1}H NMR ($CDCl_3$, 400 MHz): δ (ppm) = 6.19 – 6.02 (m, 1H, C_2-H), 5.72 – 5.61 (m, 1H, C_3-H), 4.62 (s, 2H, C_1-H, C_5-H), 4.25 (s, 1H, C_4-H), 3.71 (s, 3H, -$COOCH_3$), 2.19 (dddd, *J* = 13.2, 10.8, 8.3, 2.4 Hz, 1H, C_6/C_7-H), 1.86 (d, *J* = 5.5 Hz, 1H, C_6/C_7-H), 1.79 – 1.72 (m, 1H, C_6/C_7-H), 1.63 – 1.50 (m, 1H, C_6/C_7-H).

^{13}C NMR ($CDCl_3$, 101 MHz): δ (ppm) = 154.5 (-C=O), 134.1 (C_2), 124.6 (C_3), 58.7 (C_4, C_1/C_5), 52.5 (-$COOCH_3$, C_1/C_5), 31.7 (C_6/C_7), 29.8, 27.3 (C_6/C_7).

HRMS (APCI): m/z calculated for $C_9H_{13}ClO_2N^+$ $[M+H]^+$ 202.0629, found 202.0632.

IR: 2955, 1703, 1453, 1405, 1340, 1298, 1223, 1193, 1109, 1045, 959, 904, 824, 801, 779, 744, 708, 675, 647, 628, 603 cm^{-1}

Synthesis of Aryl- and Heteroaryl Boronic Acid Pinacol Esters

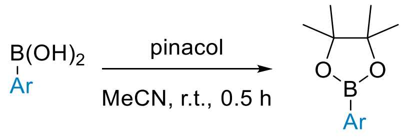

2b - **2t**

Scheme S3 Overview of synthesis of boronic acid pinacol esters **2b** – **2t**.

According to the previous procedure by González *et al.*,[8] boronic acid (1.0 equiv.) and pinacol (1.02 equiv.) are dissolved in MeCN (1.2 M) and stirred under open air for 0.5 h until the solution became clear. The result solution was concentrated *in vacuo* and the crude product was purified using automated medium-pressure chromatography (hexane/EtOAc = 95/5 to 85/15) afforded the boronic pinacol ester as a solid (>90% yield).

1.3 Rh-catalyzed Suzuki-Miyaura Reactions

1.3.1 General Procedure A

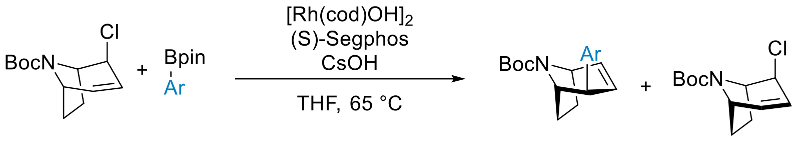

Scheme S4 Rh-catalyzed Suzuki-Miyaura cross-coupling reaction between (±)-**1a** and boronic acid pinacol esters.

$[Rh(cod)OH]_2$ (2.3 mg, 2.5 mol%) and (*S*)-Segphos (7.3 mg, 6.0 mol%) were dissolved in THF (0.5 mL) under an argon atmosphere and CsOH (50 wt% aq., 70 µL, 0.40 mmol) was added. This catalyst solution was heated to 65 °C. After 0.5 h, a solution of boronic pinacol ester (0.40 mmol) and (±)-**1a** (48.7 mg, 0.20 mmol) in THF (0.40 mL) was added into the mixture. THF (0.10 mL) was used to rinse the flask. The reaction mixture was stirred at 65 °C for 1 h. The reaction mixture was cooled to r.t.. Hexane (2 mL) and silica (1 g) were added and the solvent was removed under reduced pressure. Automated liquid chromatography was performed to purify the product using hexane/EtOAc as eluent.

Racemic samples of the products were synthesized with *rac*-BINAP (purchased from commercial suppliers) or *rac*-Segphos (mixed by manually mixing the *R* and *S*-Segphos in 1:1 ratio) as the ligand.

1.3.2 Reaction Optimization

Optimization of Leaving Groups

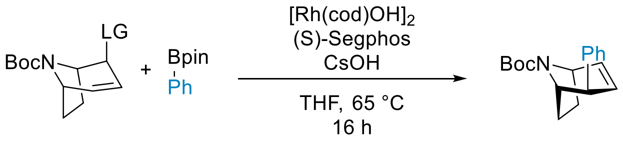

Scheme S5 Rh-catalyzed Suzuki-Miyaura cross-coupling reaction between substrates with different LG and boronic acid pinacol esters.

During optimization of the experiments, different leaving groups were screened and the results are presented in Table 1 below. The reactions were all quenched after 16 h, 4 equiv. of phenyl boronic pinacol ester and 3 equiv. of CsOH were used while all other reaction conditions were same to General Procedure A.

Table S1 Optimization Results for Different Leaving Groups

Entry	Leaving Group	Yield of **3a**/%	ee/%	d.r.	Note
1	**-F**	30	18	>20:1	-
2	**-Br**	10	78	>20:1	-
3	**-OC(O)CH$_3$**	trace	N/A	N/A	Hydrolysis to allylic alcohol
4	**-OP(O)(OEt)$_2$**	trace	N/A	N/A	Hydrolysis to allylic alcohol
5	**-OC(O)OEt**	trace	N/A	N/A	Hydrolysis to allylic alcohol
6	**-OC(O)OPh**	trace	N/A	N/A	Hydrolysis to allylic alcohol

Among reaction optimization, Design of Experiment (DoE) was performed on the methyl carbamate protected substrate (±)-1b. Different factors including numerical factors (reaction temperature, equivalents of phenyl boronic pinacol ester, equivalents of base) and categorical factors (base, solvent) were included and their correlations with yield and ee were calculated by execution of 16 different experiments with different factors generated by the JMP software, the results and the generated correlation plots are shown below. The correlation predict profiler indicated that more equivalents of the boronic pinacol ester and base are more desirable for high yielding and enantioselectivity, while CsOH and THF remained as the optimal base and solvent, and the optimal reaction temperature is 65 °C.

Table S2 Design of Experiment Optimization for (±)-**1b**

Entry	Solvent	Temperature/°C	Base	Equiv. of Ph-Bpin	Yield [a]/%	ee/%
1	Dioxane	65	RbOH (4 equiv)	1	7	24
2	THF	65	CsOH(1 equiv)	3	40	98
3	Dioxane	50	CsOH(4 equiv)	2	25	95
4	THF	50	LiOtBu (4 equiv)	4	14	45
5	Toluene	80	NaOMe(4 equiv)	3	34	88
6	Toluene	50	CsOH (2 equiv)	1	4	62
7	Toluene	65	LiOtBu (3 equiv)	2	11	85
8	THF	50	NaOMe(3 equiv)	1	27	99
9	Dioxane	50	LiOtBu (2 equiv)	3	5	35
10	Dioxane	80	CsOH (3 equiv)	4	57	90
11	Toluene	50	RbOH (1 equiv)	4	9	75
12	Dioxane	65	NaOMe(2 equiv)	4	3	61
13	THF	80	RbOH (2 equiv)	2	19	72
14	Dioxane	50	RbOH (3 equiv)	3	7	84
15	Dioxane	50	NaOMe(1 equiv)	2	4	21
16	Dioxane	80	LiOtBu(1 equiv)	1	10	3

[a] As mixture with minor diastereomer.

Figure S1 Design of Experiment fit model prediction profiler generated by JMP software.

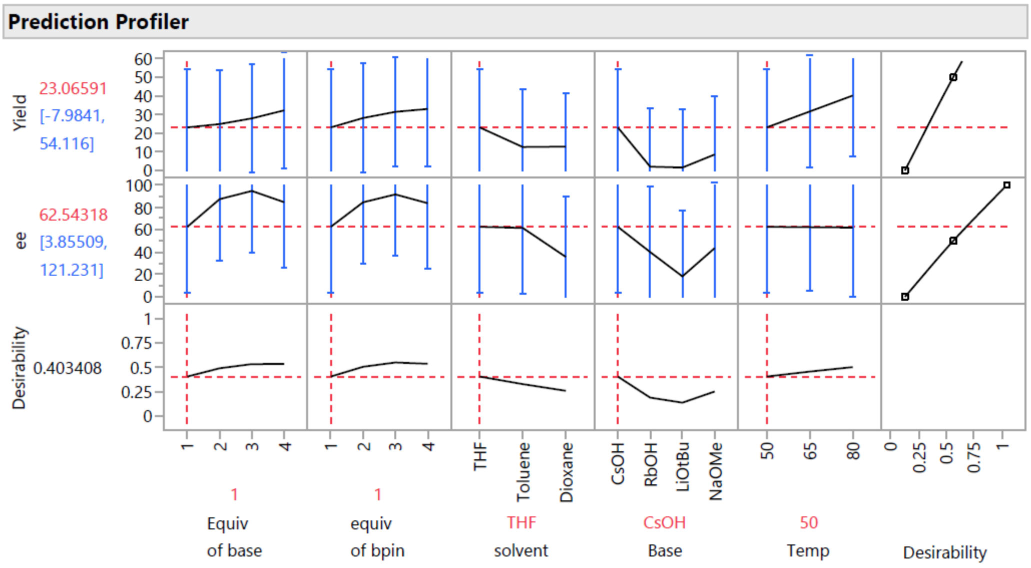

1.3.3 Product Characterizations

Synthesis of 3a

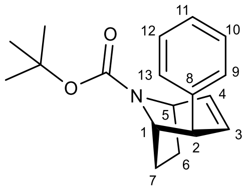

3a

The corresponding compound was prepared following General Procedure A using (±)-**1a** and phenyl boronic pinacol ester **2a**. Purification by automated medium-pressure chromatography (hexane/EtOAc = 95/5 to 90/10) afforded the product **3a** as a white solid (29 mg, 50% yield). SFC analysis showed an enantiomeric excess of 95%.

Further, (+)-**1a** was isolated as a white solid (19 mg, 39% yield). SFC analysis showed an enantiomeric excess of >99%.

^{1}H NMR ($CDCl_3$, 400 MHz): δ (ppm) = 7.35-7.26 (m, 2H, Aryl-H), 7.25-7.15 (m, 3H, Aryl-H), 6.24 (d, *J* = 8.3 Hz, 1H, C_4-H), 5.59 (ddd, *J* = 9.6, 4.1, 1.7 Hz, 1H, C_3-H), 4.70-4.48 (m, 1H, C_1/C_5-H), 4.31 (d, *J* = 7.9 Hz, 1H, C_1/C_5-H), 3.22 (dt, *J* = 3.6, 1.6 Hz, 1H, C_2-H), 2.22 (dt, *J* = 17.4, 5.7 Hz, 1H, C_6/C_7-H), 1.94-1.68 (m, 2H, C_6/C_7-H), 1.34 (m, 1H, C_6/C_7-H), 1.04 (s, 9H, -$C(CH_3)_3$)

^{13}C NMR ($CDCl_3$, 101 MHz): δ (ppm) = 152.5 (-C=O), 142.6 (C_8), 133.3 (C_4), 131.9 (rot. C_4), 128.5 (C_9, C_{13}), 128.4 (C_{10}, C_{12}), 126.5 (C_{11}), 125.5 (C_3), 78.8 (-$\underline{C}(CH_3)_3$) ,59.0 (C_1/C_5), 57.5 (rot. C_1/C_5), 53.6 (rot. C_1/C_5), 52.0 (C_1/C_5), 51.5 (C_2), 34.0 (C_6/C_7), 30.1 (C_6/C_7), 28.5 (rot. -$C(\underline{C}H_3)_3$), 28.05 (-$C(\underline{C}H_3)_3$).

HRMS (APCI): m/z calculated for $C_{18}H_{23}O_2NNa^+$ $[M+Na]^+$ 308.1621, found 308.1621.

IR: 2969, 2927, 2868, 1678, 1478, 1425, 1390, 1363, 1344, 1308, 1247, 1171, 1130, 1105, 1040, 1005, 968, 896, 875, 856, 829, 750, 717, 700, 633 cm^{-1}

Melting Point: 96- 99 °C

SFC Chiralpak ® IF; 1500 psi, 30 °C; flow: 1.5 ml/min; from 1% to 20% MeOH in 5 min (major enantiomer t_R = 2.63 min; minor enantiomer t_R = 2.93 min)

$[\alpha]^{25}_D$ = −106.7 (c = 1.0, $CHCl_3$).

Analytical data for enantioenriched (+)-1a

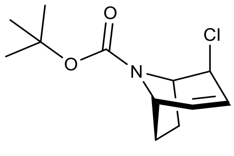

SFC Chiralpak ® IG; 1500 psi, 30 °C; flow: 1.5 ml/min; from 1% to 30% MeOH in 5 min (major enantiomer t_R = 2.33 min)

[α]^{25}$_D$ = +217.6 (c = 1.0, $CHCl_3$, ee >99%).

Synthesis of 3ab

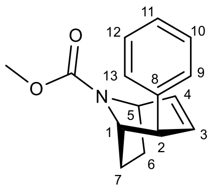

3ab

The corresponding compound was prepared following General Procedure A using (±)-**1b** and phenyl boronic pinacol ester **2a**. heating the reaction overnight. Purification by flash column chromatography (hexane/EtOAc = 100/0 to 80/20) afforded the product **3ab** as a colourless oil (33 mg, 67% yield). ^{1}H NMR showed a diastereomeric excess of 5.9:1. SFC analysis showed an enantiomeric excess of 94%.

^{1}H NMR ($CDCl_3$, 400 MHz): δ (ppm) = 7.34 – 7.28 (m, 2H, Aryl-H), 7.20 (td, *J* = 7.0, 1.3 Hz, 3H, Aryl-H), 6.24 (s, 1H, C_4-H), 5.60 (ddd, *J* = 9.7, 4.2, 1.7 Hz, 1H, C_3-H), 4.67 – 4.23 (m, 2H, C_1-H, C_5-H), 3.61 (s, 1H, C_2-H), 3.34 – 2.99 (m, 3H, -$COOCH_3$), 2.27 – 2.20 (m, 1H, C_6/C_7-H), 2.00 – 1.88 (m, 2H, C_6/C_7-H), 1.85 – 1.80 (m, 1H, C_6/C_7-H).

^{13}C NMR ($CDCl_3$, 101 MHz): δ (ppm) = 154.4 (-C=O), 132.9 (C_4), 128.4 (C_8), 128.3 (Aryl-C), 126.7 (Aryl-C), 125.6 (C_3), 59.0 (C_1/C_5), 52.9 (C_2), 51.6 (-$COOCH_3$), 34.0 (C_6/C_7), 30.0 (C_6/C_7).

HRMS (APCI): m/z calculated for $C_{15}H_{18}O_2N^+$ $[M+H]^+$ 244.1332, found 244.1333.

IR: 2923, 2854, 1703, 1453, 1405, 1334, 1194, 1109, 1045, 752, 715, 700, 665, 656, 601 cm^{-1}

SFC Chiralpak ® IE; 1500 psi, 30 °C; flow: 1.5 ml/min; from 1% to 30% MeOH in 5 min (major enantiomer t_R = 3.46 min; minor enantiomer t_R = 3.35 min)

[α]^{25}$_D$ = −47.0 (c = 1.0, $CHCl_3$).

Synthesis of 3b

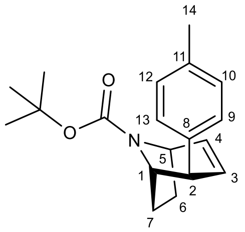

3b

The corresponding compound was prepared following General Procedure A using (±)-**1a** and **2b**. Purification by automated medium-pressure chromatography (hexane/EtOAc = 100/0 to 90/10) afforded the product **3b** as a white solid (26 mg, 44% yield). SFC analysis showed an enantiomeric excess of 96%.

Further, (+)-**1a** was isolated as a white solid (approx. 15 mg, 30% yield), contaminated with small amounts of **2b.** SFC analysis showed an enantiomeric excess of >99%.

^{1}H NMR ($CDCl_3$, 600 MHz) δ (ppm) = 7.10 (s, 4H, Aryl-H), 6.30 – 6.04 (m, 1H, C_4-H), 5.58 (ddd, *J* = 9.7, 4.2, 1.7 Hz, 1H, C_3-H), 4.65 – 4.19 (m, 2H, C_1-H, C_5-H), 3.18 (d, *J* = 4.2 Hz, 1H, C_2-H), 2.32 (s, 3H, C_{14}-H), 2.27 – 2.12 (m, 1H, C_6/C_7-H), 1.97 – 1.85 (m, 2H, C_6/C_7-H), 1.85 – 1.74 (m, 1H, C_6/C_7-H), 1.05 (s, 9H, -$C(CH_3)_3$).

^{13}C NMR ($CDCl_3$, 151 MHz) δ (ppm) = 152.5 (-C=O), 139.6 (C_8), 135.9 (C_{11}), 133.1 (C_4), 131.66 (rot. C_4), 129.0 (Aryl-C), 128.3 (Aryl-C), 128.1 (Aryl-C), 126.8 (rot. C_3), 125.7 (C_3), 79.1 (-$\underline{C}(CH_3)_3$), 59.0 (C_1/C_5), 57.6 (rot. C_1/C_5), 53.5 (rot. C_1/C_5), 52.0 (C_1/C_5), 51.2 (C_2), 34.5 (rot. C_6/C_7), 34.0 (C_6/C_7), 30.0 (C_6/C_7), 29.2 (rot. C_6/C_7), 28.5 (rot. -$C(\underline{C}H_3)_3$), 28.0 (-$C(\underline{C}H_3)_3$), 21.1 (C_{14}).

HRMS (APCI): m/z calculated for $C_{19}H_{25}O_2NNa^+$ $[M+Na]^+$ 322.1778, found 322.1775.

IR: 2974, 2928, 2871, 2360, 2341, 1693, 1513, 1478, 1422, 1391, 1365, 1339, 1303, 1251, 1230, 1173, 1132, 1104, 1062, 1042, 1022, 1007, 971, 912, 803, 783, 686, 640 cm^{-1}

Melting Point: 91.8 - 92.6 °C

SFC Chiralpak ® IB; 1500 psi, 30 °C; flow: 1.5 ml/min; from 1% to 30% MeOH in 5 min (major enantiomer t_R = 1.81 min; minor enantiomer t_R = 1.72 min)

$[\alpha]^{25}_D$ = −114.0 (c = 1.0, $CHCl_3$).

Synthesis of 3c

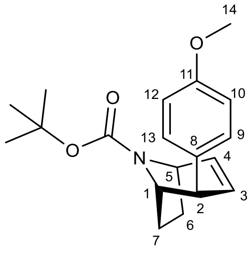

3c

The corresponding compound was prepared following General Procedure A using (±)-**1a** and **2c**. Purification by automated medium-pressure chromatography (hexane/EtOAc = 100/0 to 90/10) afforded the product **3c** as a white solid (20 mg, 32% yield). SFC analysis showed an enantiomeric excess of 94%.

Further, (+)-**1a** was isolated as a white solid (18 mg, 37% yield). SFC analysis showed an enantiomeric excess of >99%.

^{1}H NMR ($CDCl_3$, 600 MHz) δ (ppm) = 7.12 (d, J = 8.4 Hz, 2H, Aryl-H), 6.83 (d, J = 8.2 Hz, 2H, Aryl-H), 6.28 – 6.04 (m, 1H, C_4-H), 5.56 (ddd, J = 9.6, 4.2, 1.6 Hz, 1H, C_3-H), 4.63 – 4.21 (m, 2H, C_1-H, C_5-H), 3.78 (s, 3H, C_{14}-H), 3.17 (d, J = 4.1 Hz, 1H, C_2-H), 2.26 – 2.12 (m, 1H, C_6/C_7-H), 1.98 – 1.81 (m, 2H, C_6/C_7-H), 1.81 – 1.64 (m, 1H, C_6/C_7-H), 1.08 (s, 9H, -$C(CH_3)_3$).

^{13}C NMR ($CDCl_3$, 151 MHz) δ (ppm) = 158.5 (-C=O), 152.5 (C_{11}), 134.9 (C_8), 132.9 (C_4), 131.6 (rot. C_4), 129.4 (C_9/C_{13}), 129.2 (C_9/C_{13}), 126.8 (rot. C_3), 125.8 (C_3), 113.8 (C_{10}, C_{12}), 78.7 (-$\underline{C}(CH_3)_3$), 59.0 (C_1/C_5), 57.6 (rot. C_1/C_5), 55.5 (C_{14}), 53.5 (rot. C_1/C_5), 52.0 (C_1/C_5), 50.8 (C_2), 34.0 (C_6/C_7), 29.8 (C_6/C_7), 29.0 (rot. C_6/C_7), 28.5 (rot. -$C(\underline{C}H_3)_3$), 28.1 (-$C(\underline{C}H_3)_3$).

HRMS (APCI): m/z calculated for $C_{19}H_{25}O_3NNa^+$ $[M+Na]^+$ 338.1726, found 338.1724.

IR: 2934, 2835, 2349, 1689, 1611, 1510, 1423, 1340, 1246, 1177, 1106, 1038, 1007, 895, 861, 824, 786, 760, 706 cm^{-1}

Melting Point: 93.3- 94.3 °C

SFC Chiralpak ® IC; 1500 psi, 30 °C; flow: 1.5 ml/min; from 1% to 5% MeOH in 5 min (major enantiomer t_R = 4.27 min; minor enantiomer t_R = 4.08 min)

$[\alpha]^{25}_D$ = −115.1 (c = 1.0, $CHCl_3$).

Synthesis of 3d

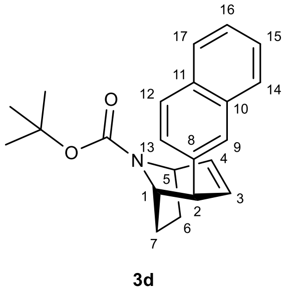

3d

The corresponding compound was prepared following General Procedure A using (±)-**1a** and **2d**. Purification by automated medium-pressure chromatography (hexane/EtOAc = 100/0 to 90/10) afforded the product **3d** as a white solid (34 mg, 51% yield). SFC analysis showed an enantiomeric excess of 95%.

Further, (+)-**1a** was isolated as a white solid (15 mg, 31% yield). SFC analysis showed an enantiomeric excess of >99%.

^{1}H NMR ($CDCl_3$, 600 MHz) δ (ppm) = 7.89 – 7.68 (m, 3H, Aryl-H), 7.60 (s, 1H, Aryl-H), 7.51 – 7.34 (m, 3H, Aryl-H), 6.38 – 6.11 (m, 1H, C_4-H), 5.70 (dd, *J* = 10.2, 4.1 Hz, 1H, C_3-H), 4.67 – 4.37 (m, 2H, C_1-H, C_5-H), 3.38 (d, *J* = 4.0 Hz, 1H, C_2-H), 2.36 – 2.17 (m, 1H, C_6/C_7-H), 2.08 – 1.91 (m, 2H, C_6/C_7-H), 1.91 – 1.72 (m, 1H, C_6/C_7-H), 1.08 (s, 9H, -C(CH_3)$_3$).

^{13}C NMR ($CDCl_3$, 151 MHz) δ (ppm) = 152.5 (-C=O), 134.0 (C_8), 133.7 (C_4), 133.6 (Aryl-C), 132.5 (rot. C_4), 132.2 (Aryl-C), 128.0 (Aryl-C), 127.9 (Aryl-C), 127.5 (Aryl-C), 127.1 (Aryl-C), 126.9 (Aryl-C), 126.0 (Aryl-C), 125.5 (C_3), 125.4 (Aryl-C), 78.6 (-$\underline{C}$(CH_3)$_3$), 58.6 (C_1/C_5), 57.4 (rot. C_1/C_5), 53.6 (rot. C_1/C_5), 52.1 (C_1/C_5), 51.6 (C_2), 34.0 (C_6/C_7), 30.1 (C_6/C_7), 28.5 (rot. -C($\underline{C}H_3$)$_3$), 27.8 (-C($\underline{C}H_3$)$_3$).

HRMS (APCI): m/z calculated for $C_{22}H_{25}O_2NNa^+$ $[M+Na]^+$ 358.1777, found 358.1774.

IR: 2974, 1689, 1421, 1339, 1305, 1247, 1174, 1133, 1104, 1043, 1008, 973, 897, 859, 815, 786, 746, 713, 626, 607 cm^{-1}

Melting Point: 134.4 – 135.0 °C

SFC Chiralpak ® IB; 1500 psi, 30 °C; flow: 1.5 ml/min; from 1% to 30% MeOH in 5 min (major enantiomer t_R = 3.00 min; minor enantiomer t_R = 2.63 min)

$[\alpha]^{25}_D$ = −228.2 (c = 1.0, $CHCl_3$).

Synthesis of 3e

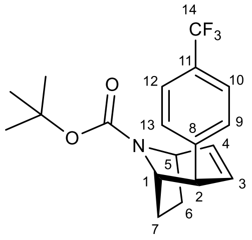

3e

The corresponding compound was prepared following General Procedure A using (±)-**1a** and **2e**. Purification by automated medium-pressure chromatography (hexane/EtOAc = 100/0 to 90/10) afforded the product **3e** as a white solid (31 mg, 44% yield). SFC analysis showed an enantiomeric excess of 94%.

Further, (+)-**1a** was isolated as a white solid (10 mg, 21% yield). SFC analysis showed an enantiomeric excess of >99%.

^{1}H NMR ($CDCl_3$, 600 MHz) δ (ppm) = 7.55 (d, *J* = 8.0 Hz, 2H, Aryl-H), 7.34 (d, *J* = 8.0 Hz, 2H, Aryl-H), 6.34 – 6.10 (m, 1H, C_4-H), 5.57 (ddd, *J* = 9.6, 4.2, 1.7 Hz, 1H, C_3-H), 4.66 – 4.19 (m, 2H, C_1-H, C_5-H), 3.28 (d, *J* = 4.4 Hz, 1H, C_2-H), 2.35 – 2.18 (m, 1H, C_6/C_7-H), 1.99 – 1.84 (m, 2H, C_6/C_7-H), 1.84 – 1.72 (m, 1H, C_6/C_7-H), 1.02 (s, 9H, -C(CH_3)$_3$).

^{13}C NMR ($CDCl_3$, 151 MHz) δ (ppm) = 152.3 (-C=O), 146.7 (C_8), 134.1 (C_4), 132.7 (rot. C_4), 129.2 (d, *J* = 129.18 Hz, C_{11}), 128.9 (C_9/C_{13}), 128.6 (C_9/C_{13}), 125.3 (q, *J* = 3.8 Hz, C_{10}, C_{12}), 124.5 (C_3), 124.5 (q, *J* = 271.9 Hz, -CF_3), 79.1 (-$\underline{C}$(CH_3)$_3$), 58.8 (C_1/C_5), 57.3 (rot. C_1/C_5), 53.6 (rot. C_1/C_5), 52.1 (C_1/C_5), 51.1 (C_2), 34.4 (rot. C_6/C_7), 33.9 (C_6/C_7), 30.0 (C_6/C_7), 29.2 (rot. C_6/C_7), 28.4 (rot. -C($\underline{C}H_3$)$_3$), 28.0 (-C($\underline{C}H_3$)$_3$).

^{19}F NMR (376 MHz, $CDCl_3$) δ (ppm) = −62.48 (s, rot. -CF_3), −62.57 (s, -CF_3).

HRMS (APCI): m/z calculated for $C_{19}H_{22}F_3O_2NNa^+$ $[M+Na]^+$ 376.1495, found 376.1495.

IR: 2360. 2341. 1678. 1477. 142. 1330, 1160, 1109, 1069, 1019, 863, 825, 778, 756, 716, 669, 655 cm^{-1}

Melting Point: 99.2 – 100.5 °C

SFC Chiralpak ® IF; 1500 psi, 30 °C; flow: 1.5 ml/min; from 1% to 5% MeOH in 5 min (major enantiomer t_R = 2.25 min; minor enantiomer t_R = 2.34 min)

$[\alpha]^{25}_D$ = −80.1 (c = 1.0, $CHCl_3$).

Synthesis of 3f

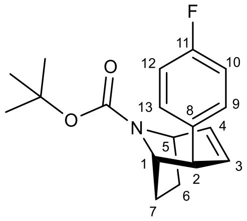

3f

The corresponding compound was prepared following General Procedure A using (±)-**1a** and **2f**. The reaction was stirred for 4 h. Purification by automated medium-pressure chromatography (hexane/EtOAc = 100/0 to 90/10) afforded the product **3f** as a white solid (30 mg, 49% yield). SFC analysis showed an enantiomeric excess of 98%.

Further, (+)-**1a** was isolated as a white solid (approx. 19 mg, 38% yield), contaminated with small amounts of **3f**. SFC analysis showed an enantiomeric excess of >99%.

^{1}H NMR ($CDCl_3$, 600 MHz) δ (ppm) = 7.23 – 7.11 (m, 2H, Aryl-H), 6.98 (t, *J* = 8.7 Hz, 2H, Aryl-H), 6.35 – 6.04 (m, 1H, C_4-H), 5.55 (ddd, *J* = 9.6, 4.3, 1.7 Hz, 1H, C_3-H), 4.68 – 4.18 (m, 2H, C_1-H, C_5-H), 3.20 (d, *J* = 4.2 Hz, 1H, C_2-H), 2.31 – 2.14 (m, 1H, C_6/C_7-H), 1.98 – 1.84 (m, 2H, C_6/C_7-H), 1.84 – 1.65 (m, 1H, C_6/C_7-H), 1.08 (s, 9H, -C(CH_3)$_3$).

^{13}C NMR ($CDCl_3$, 151 MHz) δ (ppm) = 161.8 (d, J = 244.5 Hz, C_{11}), 152.4 (-C=O), 138.2 (C_8), 133.2 (C_4), 132.0 (rot. C_4), 129.8 (d, J = 7.8 Hz, C_9, C_{13}), 126.2 (rot. C_3), 125.2 (C_3), 115.0 (d, J = 20.8 Hz, C_{10}, C_{12}), 78.8 (-$\underline{C}(CH_3)_3$), 58.8 (C_1/C_5), 57.5 (rot. C_1/C_5), 53.4 (rot. C_1/C_5), 51.9 (C_1/C_5), 50.6 (C_2), 34.3 (rot. C_6/C_7), 33.8 (C_6/C_7), 29.7 (C_6/C_7), 28.9 (rot. C_6/C_7), 28.3 (rot. -C($\underline{C}H_3)_3$), 28.0 (-C($\underline{C}H_3)_3$).

^{19}F NMR (376 MHz, $CDCl_3$) δ (ppm) = -116.72 (s, rot. C-F), -117.21 (s, C-F).

HRMS (APCI): m/z calculated for $C_{18}H_{22}$ FO_2NNa^+ $[M+ Na]^+$ 326.1527, found 326.1524.

IR: 2941, 2360, 2341, 1678, 1508, 1430, 1345, 1224, 1176, 1109 862, 724, 685 cm^{-1}

Melting Point: 91.6 - 92.5 °C

SFC Chiralpak ® IB; 1500 psi, 30 °C; flow: 1.5 ml/min; from 1% to 5% MeOH in 5 min (major enantiomer t_R = 1.91 min; minor enantiomer t_R = 1.82 min)

$[\alpha]^{25}_D$ = −113.0 (c = 1.0, $CHCl_3$).

Synthesis of 3g

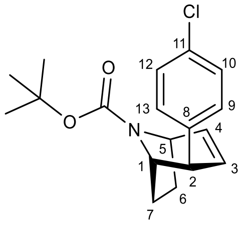

3g

The corresponding compound was prepared following General Procedure A using (±)-**1a** and **2g**. Purification by automated medium-pressure chromatography (hexane/EtOAc = 100/0 to 90/10) afforded the product **3g** as a white solid (30 mg, 47% yield). SFC analysis showed an enantiomeric excess of 96%.

Further, (+)-**1a** was isolated as a white solid (12 mg, 25% yield). SFC analysis showed an enantiomeric excess of >99%.

^{1}H NMR ($CDCl_3$, 600 MHz) δ (ppm) = 7.38 – 7.18 (m, 2H, Aryl-H), 7.21 – 7.05 (m, 2H, Aryl-H), 6.35 – 6.03 (m, 1H, C_4-H), 5.62 – 5.49 (m, 1H C_3-H), 4.76 – 4.13 (m, 2H, C_1-H, C_5-H), 3.21 (d, J = 4.1 Hz, 1H, C_2-H), 2.31 – 2.17 (m, 1H, C_6/C_7-H), 1.96 – 1.88 (m, 2H, C_6/C_7-H), 1.88 – 1.73 (m, 1H, C_6/C_7-H), 1.10 (s, 9H, -C($CH_3)_3$).

^{13}C NMR ($CDCl_3$, 151 MHz) δ (ppm) = 152.4 (-C=O), 141.1 (C_8), 133.6 (C_4), 132.4 (C_{11}), 129.9 (Aryl-C), 129.6 (Aryl-C), 128.4 (Aryl-C), 126.1 (rot. C_3), 125.0 (C_3), 79.0 (-$\underline{C}(CH_3)_3$), 58.8 (C_1/C_5), 57.4 (rot. C_1/C_5), 53.5 (rot. C_1/C_5), 52.1 (C_1/C_5), 50.8 (C_2), 34.4 (rot. C_6/C_7), 29.9 (C_6/C_7), 29.1 (rot. C_6/C_7), 28.5 (rot. -C($\underline{C}H_3)_3$), 28.1 (-C($\underline{C}H_3)_3$).

HRMS (APCI): m/z calculated for $C_{18}Cl_1H_{22}O_2NNa^+$ $[M+Na]^+$ 342.1231, found 342.1229.

IR: 2943, 2834, 1676, 1427, 1109, 1025, 689 cm^{-1}

Melting Point: 117.8 – 119.4 °C

SFC Chiralpak ® IB; 1500 psi, 30 °C; flow: 1.5 ml/min; from 1% to 30% MeOH in 5 min (major enantiomer t_R = 1.98 min; minor enantiomer t_R = 1.88 min)

$[\alpha]^{25}_D$ = −80.6 (c = 1.0, $CHCl_3$).

Synthesis of 3h

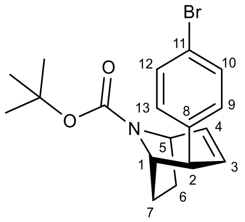

3h

The corresponding compound was prepared following General Procedure A using (±)-**1a** and **2h**. Purification by automated medium-pressure chromatography (hexane/EtOAc = 100/0 to 90/10) afforded the product **3h** as a white solid (28 mg, 39% yield). SFC analysis showed an enantiomeric excess of 96%.

Further, (+)-**1a** was isolated as a white solid (approx. 17 mg, 35% yield), contaminated with small amounts of **2h**. SFC analysis showed an enantiomeric excess of >99%.

^{1}H NMR ($CDCl_3$, 600 MHz) δ (ppm) = 7.48 – 7.32 (m, 2H, Aryl-H), 7.12 – 7.05 (m, 2H, Aryl-H), 6.29 – 6.07 (m, 1H, C_4-H), 5.54 (ddd, *J* = 9.6, 4.2, 1.7 Hz, 1H, C_3-H), 4.68 – 4.13 (m, 2H, C_1-H, C_5-H), 3.18 (d, *J* = 4.2 Hz, 1H, C_2-H), 2.28 – 2.16 (m, 1H, C_6/C_7-H), 1.97 – 1.82 (m, 2H, C_6/C_7-H), 1.82 – 1.68 (m, 1H, C_6/C_7-H), 1.24 (s, 9H, -$C(CH_3)_3$).

^{13}C NMR ($CDCl_3$, 151 MHz) δ (ppm) = 152.4 (-C=O), 141.6 (C_8), 133.7 (C_4), 132.3 (rot. C_4), 131.4 (Aryl-C), 130.3 (Aryl-C), 126.0 (rot. C_3), 124.9 (C_3), 120.4 (C_{11}), 79.1 (-$\underline{C}(CH_3)_3$), 58.8 (C_1/C_5), 57.4 (rot. C_1/C_5), 53.6 (rot. C_1/C_5), 52.1 (C_1/C_5), 50.9 (C_2), 34.4 (rot. C_6/C_7), 33.9 (C_6/C_7), 29.9 (C_6/C_7), 29.1 (rot. C_6/C_7), 28.5 (rot. -$C(\underline{C}H_3)_3$), 28.1 (-$C(\underline{C}H_3)_3$).

HRMS (APCI): m/z calculated for $C_{18}H_{22}BrO_2NNa^+$ $[M+Na]^+$ 386.0726 and 388.0706, found 386.0725 and 388.0704.

IR: 2974, 1692, 1487, 1420, 1339, 1171, 1132, 1105, 1072, 1043, 1010, 972, 894, 858, 818, 758, 724, 658 cm^{-1}

Melting Point: 103.6 – 104.1 °C

SFC Chiralpak ® IB; 1500 psi, 30 °C; flow: 1.5 ml/min; from 1% to 30% MeOH in 5 min (major enantiomer t_R = 2.17 min; minor enantiomer t_R = 2.07 min)

[α]$^{25}_{D}$ = −85.7 (c = 1.0, $CHCl_3$).

Synthesis of 3i

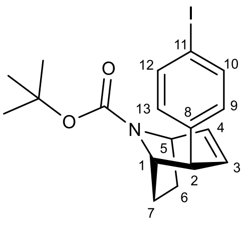

3i

The corresponding compound was prepared following General Procedure A using (±)-**1a** and **2i**. Purification by automated medium-pressure chromatography (hexane/EtOAc = 100/0 to 90/10) afforded the product **3i** as a white solid (18 mg, 22% yield). SFC analysis showed an enantiomeric excess of 96%.

Further, (+)-**1a** was isolated as a white solid (approx. 32 mg, 66% yield), contaminated with small amounts of **2i**. SFC analysis showed an enantiomeric excess of 59%.

^{1}H NMR ($CDCl_3$, 600 MHz) δ (ppm) = 7.75 – 7.50 (m, 2H, Aryl-H), 7.07 – 6.87 (m, 2H, Aryl-H), 6.29 – 6.09 (m, 1H, C_4-H), 5.54 (ddd, *J* = 9.6, 4.2, 1.7 Hz, 1H, C_3-H), 4.70 – 4.07 (m, 2H, C_1-H, C_5-H), 3.16 (d, *J* = 4.1 Hz, 1H, C_2-H), 2.31 – 2.10 (m, 1H, C_6/C_7-H), 1.96 – 1.81 (m, 2H, C_6/C_7-H), 1.81 – 1.59 (m, 1H, C_6/C_7-H), 1.24 (s, 9H, -C(CH_3)$_3$).

^{13}C NMR ($CDCl_3$, 151 MHz) δ (ppm) = 152.4 (-C=O), 142.4 (C_8), 137.4 (C_{10}, C_{12}), 133.8 (C_4), 132.4 (rot. C_4), 130.6 (C_9, C_{13}), 126.0 (rot. C_3), 124.8 (C_3), 91.8 (C_{11}), 79.1 (-$\underline{C}$(CH_3)$_3$), 58.8 (C_1/C_5), 57.4 (rot. C_1/C_5), 53.6 (rot. C_1/C_5), 52.1 (C_1/C_5), 50.9 (C_2), 34.4 (rot. C_6/C_7), 33.9 (C_6/C_7), 29.9 (C_6/C_7), 29.2 (rot. C_6/C_7), 28.5 (rot. -C($\underline{C}H_3$)$_3$), 28.1 (-C($\underline{C}H_3$)$_3$).

HRMS (APCI): m/z calculated for $C_{18}H_{22}IO_2NNa^+$ $[M+Na]^+$ 434.0587, found 434.0583.

IR: 2974, 1690, 1482, 1365, 1300, 1248, 1172, 1132, 1104, 1062, 1042, 1006, 972, 894, 857, 817, 758, 723, 650 cm^{-1}

Melting Point: 104.7 – 105.3 °C

SFC Chiralpak ® IB; 1500 psi, 30 °C; flow: 1.5 ml/min; from 1% to 30% MeOH in 5 min (major enantiomer t_R = 2.41 min; minor enantiomer t_R = 2.33 min)

[α]$^{25}_{D}$ = −99.6 (c = 1.0, $CHCl_3$).

Synthesis of 3j

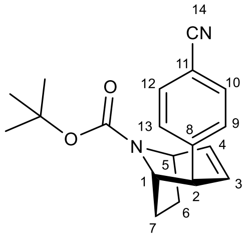

3j

The corresponding compound was prepared following General Procedure A using (±)-**1a** and **2j**. The reaction was stirred overnight at 80 °C in 1,4-dioxane. Purification by automated medium-pressure chromatography (hexane/EtOAc = 100/0 to 90/10) afforded the product **3j** as a white solid (14 mg, 23% yield). ^{1}H NMR integration showed a diastereomeric ratio of 18:1. SFC analysis showed an enantiomeric excess of 97%.

Further, (+)-**1a** was isolated as a white solid (approx. 22 mg, 46% yield), contaminated with small amounts of **2j**. SFC analysis showed an enantiomeric excess of 39%.

^{1}H NMR ($CDCl_3$, 600 MHz) δ (ppm) = 7.69 – 7.48 (m, 2H, Aryl-H), 7.34 (d, *J* = 7.9 Hz, 2H, Aryl-H), 6.36 – 6.13 (m, 1H, C_4-H), 5.55 (ddd, *J* = 9.6, 4.2, 1.7 Hz, 1H, C_3-H), 4.67 – 4.18 (m, 2H, C_1-H, C_5-H), 3.27 (d, *J* = 4.1 Hz, 1H, C_2-H), 2.28 – 2.18 (m, 1H, C_6/C_7-H), 1.96 – 1.86 (m, 2H, C_6/C_7-H), 1.86 – 1.71 (m, 1H, C_6/C_7-H), 1.16 (s, 9H, -$C(CH_3)_3$).

^{13}C NMR ($CDCl_3$, 151 MHz) δ (ppm) = 152.3 (-C=O), 148.2 (C_8), 134.4 (C_4), 132.2 (Aryl-C), 129.4 (Aryl-C), 124.1 (C_3), 119.2 (-CN), 110.4 (C_{11}), 79.2 (-$\underline{C}(CH_3)_3$), 58.7 (C_1/C_5), 53.6 (rot. C_1/C_5), 52.2 (C_1/C_5), 51.3 (C_2), 34.4 (rot. C_6/C_7), 33.9 (C_6/C_7), 30.1 (rot. C_6/C_7), 29.9 (C_6/C_7), 28.4 (rot. -$C(\underline{C}H_3)_3$), 28.1 (-$C(\underline{C}H_3)_3$).

HRMS (APCI): m/z calculated for $C_{19}H_{22}O_2N_2Na^+$ $[M+Na]^+$ 333.1573, found 333.1573.

IR: 2974, 1690, 1482, 1365, 1300, 1248, 1172, 1132, 1104, 1062, 1042, 1006, 972, 894, 857, 817, 758, 723, 650 cm^{-1}

Melting Point: 103.8 – 104.9 °C

SFC Chiralpak ® IB; 1500 psi, 30 °C; flow: 1.5 ml/min; from 1% to 30% MeOH in 5 min (major enantiomer t_R = 2.24 min; minor enantiomer t_R = 2.17 min)

$[\alpha]^{25}_D$ = −104.4 (c = 0.5, $CHCl_3$).

Synthesis of 3k

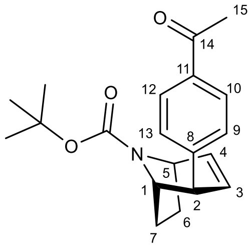

3k

The corresponding compound was prepared following General Procedure A using (±)-**1a** and **2k**. Purification by automated medium-pressure chromatography (hexane/EtOAc = 100/0 to 75/25) afforded the product **3k** as a white solid (12 mg, 19% yield). SFC analysis showed an enantiomeric excess of 98%.

Further, (+)-**1a** was isolated as a white solid (approx. 32 mg, 65% yield), contaminated with small amounts of **2k**. SFC analysis showed an enantiomeric excess of 31%.

^{1}H NMR ($CDCl_3$, 600 MHz) δ (ppm) = 7.89 (d, *J* = 7.9 Hz, 2H, Aryl-H), 7.32 (d, *J* = 8.0 Hz, 2H, Aryl-H), 6.36 – 6.15 (m, 1H, C_4-H), 5.58 (ddd, *J* = 9.7, 4.3, 1.5 Hz, 1H, C_3-H), 4.79 – 4.27 (m, 2H, C_1-H, C_5-H), 3.27 (d, *J* = 4.1 Hz, 1H, C_2-H), 2.58 (s, 3H, C_{15}-H), 2.30 – 2.12 (m, 1H, C_6/C_7-H), 1.98 – 1.83 (m, 2H, C_6/C_7-H), 1.83 – 1.69 (m, 1H, C_6/C_7-H), 1.20 (s, 9H, -C(CH_3)$_3$).

^{13}C NMR ($CDCl_3$, 151 MHz) δ (ppm) = 198.1 (C_{14}), 152.4 (-C=O), 148.2 (C_8), 135.7 (C_{11}), 133.9 (C_4), 132.7 (rot. C_4), 128.8 (Aryl-C), 128.5 (Aryl-C), 125.6 (rot. C_3), 124.7 (C_3), 79.0 (-$\underline{C}$(CH_3)$_3$), 58.7 (C_1/C_5), 57.3 (rot. C_1/C_5), 53.6 (rot. C_1/C_5), 52.2 (C_1/C_5), 51.4 (C_2), 34.5 (rot. C_6/C_7), 34.0 (C_6/C_7), 30.1 (C_6/C_7), 29.3 (rot. C_6/C_7), 28.5 (rot. -C($\underline{C}H_3$)$_3$), 28.1 (-C($\underline{C}H_3$)$_3$), 26.8 (C_{15}).

HRMS (APCI): m/z calculated for $C_{20}H_{25}O_3NNa^+$ $[M+Na]^+$ 350.1727, found 350.1728.

IR: 2974, 1684, 1606, 1422, 1365, 1340, 1269, 1173, 1105, 1043, 973, 861, 755, 664 cm^{-1}

Melting Point: 116.7 – 117.4 °C

SFC Chiralpak ® IF; 1500 psi, 30 °C; flow: 1.5 ml/min; from 1% to 30% MeOH in 5 min (major enantiomer t_R = 3.58 min; minor enantiomer t_R = 3.46 min)

$[\alpha]^{25}_D$ = −112.2 (c = 0.5, $CHCl_3$).

Synthesis of 3l

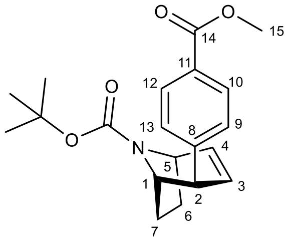

3l

The corresponding compound was prepared following General Procedure A using (±)-**1a** and **2l**. Purification by automated medium-pressure chromatography (hexane/EtOAc = 100/0 to 75/25) afforded the product **3l** as a white solid (41 mg, 59% yield). SFC analysis showed an enantiomeric excess of 96%.

Further, (+)-**1a** was isolated as a white solid (approx. 13 mg, 27% yield), contaminated with small amounts of **2l**. SFC analysis showed an enantiomeric excess of >99%.

^{1}H NMR ($CDCl_3$, 600 MHz) δ (ppm) = 7.96 (d, *J* = 7.9 Hz, 2H, Aryl-H), 7.29 (d, 2H, Aryl-H), 6.33 – 6.13 (m, 1H, C_4-H), 5.57 (ddd, *J* = 9.6, 4.1, 1.7 Hz, 1H, C_3-H), 4.65 – 4.19 (m, 2H, C_1-H, C_5-H), 3.90 (s, 3H, C_{15}-H), 3.33 – 3.21 (m, 1H, C_2-H), 2.36 – 2.15 (m, 1H, C_6/C_7-H), 1.97 – 1.86 (m, 2H, C_6/C_7-H), 1.86 – 1.71 (m, 1H, C_6/C_7-H), 1.14 (s, 9H, -$C(CH_3)_3$).

^{13}C NMR ($CDCl_3$, 151 MHz) δ (ppm) = 167.3 (C_{14}), 152.4 (-C=O), 148.0 (C_8), 133.8 (C_4), 132.6 (rot. C_4), 129.7 (2 × Aryl-C), 128.6 (2 × Aryl-C), 128.4 (C_{11}), 125.7 (rot. C_3), 124.8 (C_3), 79.0 (-$\underline{C}(CH_3)_3$), 58.7 (C_1/C_5), 57.4 (rot. C_1/C_5), 53.6 (rot. C_1/C_5), 52.2 (C_1/C_5), 52.1 (C_2), 51.4 (C_{15}), 34.4 (rot. C_6/C_7), 33.9 (C_6/C_7), 30.2 (C_6/C_7), 29.3 (rot. C_6/C_7), 28.5 (rot. -$C(\underline{C}H_3)_3$), 28.1 (-$C(\underline{C}H_3)_3$).

HRMS (APCI): m/z calculated for $C_{20}H_{25}O_4NNa^+$ $[M+Na]^+$ 366.1676, found 366.1673.

IR: 2974, 2926, 2874, 1721, 1693, 1574, 1477, 1420, 1391, 1366, 1340, 1312, 1279, 1174, 1157, 1132, 1104, 1062, 1043, 1020, 1008, 971, 913, 895, 877, 862, 826, 762, 720, 707, 662 cm^{-1}

Melting Point: 98.5 – 98.8 °C

SFC Chiralpak ® IC; 1500 psi, 30 °C; flow: 1.5 ml/min; from 1% to 30% MeOH in 5 min (major enantiomer t_R = 3.58 min; minor enantiomer t_R = 3.46 min)

$[\alpha]^{25}_D$ = −102.9 (c = 1.0, $CHCl_3$).

Synthesis of 3m

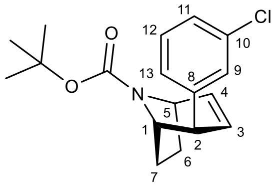

3m

The corresponding compound was prepared following General Procedure A using (±)-**1a** and **2m**. Purification by automated medium-pressure chromatography (hexane/EtOAc = 100/0 to 90/10) afforded the product **3m** as a white solid (24 mg, 38% yield). SFC analysis showed an enantiomeric excess of 96%.

Further, (+)-**1a** was isolated as a white solid (6 mg, 12% yield). SFC analysis showed an enantiomeric excess of >99%.

^{1}H NMR ($CDCl_3$, 600 MHz) δ (ppm) = 7.24 – 7.16 (m, 3H, Aryl-H), 7.15 – 7.08 (m, 1H, Aryl-H), 6.30 – 6.13 (m, 1H, C_4-H), 5.55 (ddd, *J* = 9.6, 4.1, 1.7 Hz, 1H, C_3-H), 4.66 – 4.22 (m, 2H, C_1-H, C_5-H), 3.19 (d, *J* = 4.0 Hz, 1H, C_2-H), 2.30 – 2.16 (m, 1H, C_6/C_7-H), 1.95 – 1.79 (m, 2H, C_6/C_7-H), 1.84 – 1.67 (m, 1H, C_6/C_7-H), 1.08 (s, 9H, -$C(CH_3)_3$).

^{13}C NMR ($CDCl_3$, 151 MHz) δ (ppm) = 152.4 (-C=O), 144.8 (C_8), 134.3 (C_{10}), 133.9 (C_4), 132.7 (rot. C_4), 129.6 (Aryl-C), 128.6 (Aryl-C), 127.0 (Aryl-H), 126.7 (Aryl-H), 125.7 (rot. C_3), 124.8 (C_3), 79.0 (-$\underline{C}(CH_3)_3$), 58.8 (C_1/C_5), 57.4 (rot. C_1/C_5), 53.5 (rot. C_1/C_5), 52.1 (C_1/C_5), 51.0 (C_2), 34.4 (rot. C_6/C_7), 33.9 (C_6/C_7), 30.0 (C_6/C_7), 29.1 (rot. C_6/C_7), 28.5 (rot. -$C(\underline{C}H_3)_3$), 28.1 (-$C(\underline{C}H_3)_3$).

HRMS (APCI): m/z calculated for $C_{18}H_{22}ClO_2NNa^+$ $[M+Na]^+$ 342.1231, found 342.1230.

IR: 2974, 2928, 2872, 2362, 1692, 1596, 1572, 1476, 1421, 1391, 1366, 1339, 1315, 1294, 1249, 1230, 1173, 1157, 1133, 1104, 1080, 1061, 1043, 1008, 974, 921, 892, 864, 846, 829, 772, 729, 690 cm^{-1}

Melting Point: 90.7 – 91.8 °C

SFC Chiralpak ® ID; 1500 psi, 30 °C; flow: 1.5 ml/min; from 1% to 30% MeOH in 5 min (major enantiomer t_R = 2.29 min; minor enantiomer t_R = 2.42 min)

$[\alpha]^{25}_D$ = −128.6 (c = 1.0, $CHCl_3$).

Synthesis of 3n

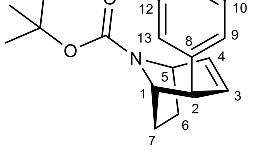

3n

The corresponding compound was prepared following General Procedure A using (±)-**1a** and **2n**. Purification by automated medium-pressure chromatography (hexane/EtOAc = 100/0 to 90/10) afforded the product **3n** as a white solid (31 mg, 49% yield). SFC analysis showed an enantiomeric excess of 97%.

Further, (+)-**1a** was isolated as a white solid (approx. 22 mg, 46% yield), contaminated with small amounts of **2n**. SFC analysis showed an enantiomeric excess of >99%.

^{1}H NMR ($CDCl_3$, 600 MHz) δ (ppm) = 6.76 (d, J = 7.7 Hz, 2H, Aryl-H), 6.70 – 6.59 (m, 1H, Aryl-H), 6.31 – 6.13 (m, 1H, C_4-H), 5.53 (ddd, J = 9.6, 4.3, 1.7 Hz, 1H, C_3-H), 4.68 – 4.20 (m, 2H, C_1-H, C_5-H), 3.18 (d, J = 4.2 Hz, 1H, C_2-H), 2.31 – 2.16 (m, 1H, C_6/C_7-H), 1.97 – 1.82 (m, 2H, C_6/C_7-H), 1.82 – 1.68 (m, 1H, C_6/C_7-H), 1.26 (s, 9H, -C(CH_3)$_3$).

^{13}C NMR ($CDCl_3$, 151 MHz) δ (ppm) = 163.1 (dd, J = 247.8, 12.7 Hz, C_{10}, C_{12}), 152.7 (-C=O), 152.6 (d, J = 87.3 Hz, C_9/C_{13}), 146.5 (d, J = 77.0 Hz, C_9/C_{13}), 134.4 (C_4), 133.2 (rot. C_4), 125.17 (rot. C_3), 124.3 (C_3), 111.3 (d, J = 25.5 Hz, C_8), 101.93 (t, J = 25.5 Hz, C_{11}), 79.1 (-$\underline{C}$(CH_3)$_3$), 58.6 (C_1/C_5), 57.3 (rot. C_1/C_5), 53.5 (rot. C_1/C_5), 52.07 (C_1/C_5), 50.9 (C_2), 34.3 (rot. C_6/C_7), 33.8 (C_6/C_7), 30.0 (C_6/C_7), 29.1 (rot. C_6/C_7), 28.4 (rot. -C($\underline{C}H_3$)$_3$), 28.1 (-C($\underline{C}H_3$)$_3$).

^{19}F NMR (377 MHz, $CDCl_3$) δ (ppm) = −110.71 to −110.75 (br. m, C-F).

HRMS (APCI): m/z calculated for $C_{18}H_{21}F_2O_2NNa^+$ $[M+Na]^+$ 344.1433, found 344.1430.

IR: 2929,1684, 1625, 1597, 1456, 1428, 1392, 1367, 1343, 1319, 1216, 1172, 1116, 1045 993, 952, 894, 845, 754, 684, 667, 628 cm^{-1}

Melting Point: 78.3 – 79.2 °C

SFC Chiralpak ® ID; 1500 psi, 30 °C; flow: 1.5 ml/min; from 1% to 30% MeOH in 5 min (major enantiomer t_R = 1.73 min; minor enantiomer t_R = 1.93 min)

$[\alpha]^{25}_D$ = −97.4 (c = 1.0, $CHCl_3$).

Synthesis of 3o

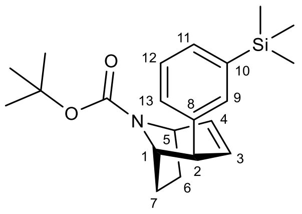

3o

The corresponding compound was prepared following General Procedure A using (±)-**1a** and **2o**. Purification by automated medium-pressure chromatography (hexane/EtOAc = 100/0 to 90/10) afforded the product **3o** as a colourless oil (33 mg, 46% yield). SFC analysis showed an enantiomeric excess of 97%.

Further, (+)-**1a** was isolated as a white solid (18 mg, 36% yield). SFC analysis showed an enantiomeric excess of >99%.

^{1}H NMR ($CDCl_3$, 600 MHz) δ (ppm) = 7.40 – 7.31 (m, 2H, Aryl-H), 7.28 (t, *J* = 7.4 Hz, 1H, Aryl-H), 7.19 (d, *J* = 7.8 Hz, 1H, Aryl-H), 6.30 – 6.18 (m, 1H, C_4-H), 5.65 – 5.52 (m, 1H, C_3-H), 4.67 – 4.22 (m, 2H, C_1-H, C_5-H), 3.32 – 3.20 (m, 1H, C_2-H), 2.32 – 2.09 (m, 1H, C_6/C_7-H), 1.96 – 1.86 (m, 2H, C_6/C_7-H), 1.86 – 1.77 (m, 1H, C_6/C_7-H), 1.20 (s, 9H, -$C(CH_3)_3$), 0.25 (s, 9H, -$Si(CH_3)_3$).

^{13}C NMR ($CDCl_3$, 151 MHz) δ (ppm) = 152.4 (-C=O), 141.7 (C_{10}), 140.3 (C_8), 133.4 (C_4), 133.3 (Aryl-C), 131.5 (Aryl-C), 129.0 (Aryl-C), 127.9 (Aryl-C), 126.9 (rot. C_3), 125.4 (C_3), 78.6 (-$\underline{C}(CH_3)_3$), 59.2 (C_1/C_5), 57.4 (rot. C_1/C_5), 53.6 (rot. C_1/C_5), 52.1 (C_1/C_5), 51.5 (C_2), 34.5 (rot. C_6/C_7), 30.0 (C_6/C_7), 29.4 (rot. C_6/C_7), 28.6 (rot. -$C(\underline{C}H_3)_3$), 28.0 (-$C(\underline{C}H_3)_3$), −0.9 (-$Si(CH_3)_3$).

HRMS (APCI): m/z calculated for $C_{21}H_{31}O_2NSiNa^+$ $[M+Na]^+$ 380.2016, found 380.2014.

IR: 2951, 2871, 1745, 1695, 1613, 1422, 1366, 1340, 1296, 1249, 1172, 1105, 1052, 1015, 977, 946, 867, 838, 771, 753, 713, 693 cm^{-1}

SFC Chiralpak ® IC; 1500 psi, 30 °C; flow: 1.5 ml/min; from 1% to 20% MeOH in 5 min (major enantiomer t_R = 2.33 min; minor enantiomer t_R = 2.24 min)

$[\alpha]^{25}_D$ = −54.9 (c = 1.0, $CHCl_3$).

Synthesis of 3p

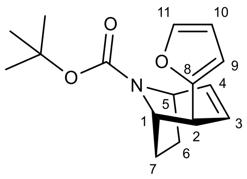

3p

The corresponding compound was prepared following General Procedure A using (±)-**1a** and **2p**. The reaction was stirred for 2 h. Purification by automated medium-pressure chromatography (hexane/EtOAc = 100/0 to 90/10) afforded the product **3p** as a white solid (31 mg, 56% yield). SFC analysis showed an enantiomeric excess of 97%.

Further, (+)-**1a** was isolated as a white solid (14 mg, 28% yield). SFC analysis showed an enantiomeric excess of >99%.

^{1}H NMR ($CDCl_3$, 600 MHz) δ (ppm) = 7.37 (s, 1H, Aryl-H), 6.33 – 6.25 (m, 1H, C_4-H), 6.24 – 5.93 (m, 2H, Aryl-H), 5.60 (dd, *J* = 9.9, 4.0 Hz, 1H, C_3-H), 4.69 – 4.38 (m, 2H, C_1-H, C_5-H), 3.24 (d, *J* = 4.2 Hz, 1H, C_2-H), 2.31 – 2.18 (m, 1H, C_6/C_7-H), 1.97 – 1.85 (m, 2H, C_6/C_7-H), 1.82 – 1.68 (m, 1H, C_6/C_7-H), 1.20 (s, 9H, -$C(CH_3)_3$).

^{13}C NMR ($CDCl_3$, 151 MHz) δ (ppm) = 155.6 (-C=O), 152.6 (C_8), 141.5 (C_{11}), 134.6 (C_4), 133.0 (rot. C_4), 123.8 (rot. C_3), 122.9 (C_3), 110.5 (C_{10}), 107.0 (C_9), 79.0 (-$\underline{C}(CH_3)_3$), 55.7 (C_1/C_5), 55.1 (rot. C_1/C_5), 53.5 (rot. C_1/C_5), 52.0 (C_1/C_5), 44.9 (C_2), 34.3 (rot. C_6/C_7), 33.9 (C_6/C_7), 29.4 (C_6/C_7), 28.5 (rot. -$C(\underline{C}H_3)_3$), 28.1 (-$C(\underline{C}H_3)_3$).

HRMS (APCI): m/z calculated for $C_{16}H_{21}O_3NNa^+$ $[M+Na]^+$ 298.1414, found 298.1414.

IR: 2360, 2344, 1694, 1421, 1340, 1249, 1227, 1175, 1141, 1106, 1044, 1005, 971, 870, 830, 791, 734, 668, 647, 640, 635, 625, 618, 608 cm^{-1}

Melting Point: 224 – 226 °C

SFC Chiralpak ® ID; 1500 psi, 30 °C; flow: 1.5 ml/min; from 1% to 20% MeOH in 5 min (major enantiomer t_R = 3.50 min; minor enantiomer t_R = 3.89 min)

$[\alpha]^{25}_D$ = −30.8 (c = 1.0, $CHCl_3$).

Synthesis of 3q

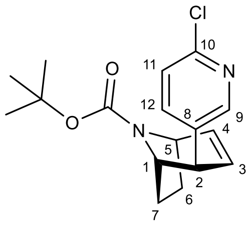

3q

The corresponding compound was prepared following General Procedure A using (±)-**1a** and **2q**. The reaction was stirred for 2 h. Purification by automated medium-pressure chromatography (hexane/EtOAc = 100/0 to 70/30) afforded the product **3q** as a white solid (27 mg, 42% yield). SFC analysis showed an enantiomeric excess of 97%.

Further, (+)-**1a** was isolated as a white solid (approx. 13 mg, 26% yield), contaminated with small amounts of **2q**. SFC analysis showed an enantiomeric excess of >99%.

^{1}H NMR ($CDCl_3$, 600 MHz) δ (ppm) = 8.25 (s, 1H, Aryl-H), 7.51 (dd, *J* = 8.0, 2.5 Hz, 1H, Aryl-H), 7.40 – 7.15 (m, 1H, Aryl-H), 6.35 – 6.09 (m, 1H, C_4-H), 5.52 (ddd, *J* = 9.6, 4.2, 1.7 Hz, 1H, C_3-H), 4.76 – 4.17 (m, 2H, C_1-H, C_5-H), 3.23 (d, *J* = 4.1 Hz, 1H, C_2-H), 2.33 – 2.08 (m, 1H, C_6/C_7-H), 2.04 – 1.82 (m, 2H, C_6/C_7-H), 1.82 – 1.75 (m, 1H, C_6/C_7-H), 1.25 (s, 9H, -$C(CH_3)_3$).

^{13}C NMR ($CDCl_3$, 151 MHz) δ (ppm) = 152.4 (-C=O), 149.8 (C_{10}), 149.6 (C_9), 138.9 (Aryl-C), 137.0 (Aryl-C), 134.5 (C_4), 133.2 (rot. C_4), 124.9 (rot. C_3), 123.9 (Aryl-C, C_3), 79.5 (-$\underline{C}(CH_3)_3$), 58.7 (C_1/C_5), 57.3 (rot. C_1/C_5), 53.6 (rot. C_1/C_5), 52.2 (C_1/C_5), 48.2 (C_2), 34.3 (rot. C_6/C_7), 33.8 (C_6/C_7), 30.5 (rot. C_6/C_7), 29.9 (C_6/C_7), 29.1 (rot. -$C(\underline{C}H_3)_3$), 28.2 (-$C(\underline{C}H_3)_3$).

HRMS (APCI): m/z calculated for $C_{17}H_{22}ClO_2N_2^+$ $[M+H]^+$ 321.1364, found 321.1362.

IR: 2981, 1683, 1426, 1216, 1107, 753, 668 cm^{-1}

Melting Point: 106.0 – 106.7 °C

SFC Chiralpak ® IA; 1500 psi, 30 °C; flow: 1.5 ml/min; from 1% to 30% MeOH in 5 min (major enantiomer t_R = 3.30 min; minor enantiomer t_R = 3.17 min)

[α]^{25}$_D$ = −106.8 (c = 1.0, $CHCl_3$).

Synthesis of 3r

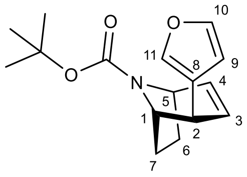

3r

The corresponding compound was prepared following General Procedure A using (±)-**1a** and **2r**. The reaction was stirred for 2 h. Purification by automated medium-pressure chromatography (hexane/EtOAc = 100/0 to 90/10) afforded the product as a white solid (8 mg, 15% yield). ^{1}H NMR integration showed a diastereomeric ratio of 17:1. SFC analysis showed an enantiomeric excess of 98%.

Further, (+)-**1a** was isolated as a white solid (32 mg, 65% yield). SFC analysis showed an enantiomeric excess of 26%.

^{1}H NMR ($CDCl_3$, 600 MHz) δ (ppm) = 7.40 (s, 1H, Aryl-H), 7.17 (s, 1H, Aryl-H), 6.39 – 6.32 (m, 1H, Aryl-H), 6.20 – 5.96 (m, 1H, C_4-H), 5.60 (ddd, *J* = 9.6, 4.2, 1.7 Hz, 1H, C_3-H), 4.74 – 4.13 (m, 2H, C_1-H, C_5-H), 3.09 (s, 1H, C_2-H), 2.29 – 2.12 (m, 1H, C_6/C_7-H), 1.96 – 1.81 (m, 2H, C_6/C_7-H), 1.81 – 1.66 (m, 1H, C_6/C_7-H), 1.31 (s, 9H, -$C(CH_3)_3$).

^{13}C NMR ($CDCl_3$, 151 MHz) δ (ppm) = 152.8 (-C=O), 142.8 (C_{10}), 140.2 (C_{11}), 132.9 (C_4), 131.3 (rot. C_4), 126.6 (C_8), 126.4 (rot. C_3), 125.2 (C_3), 110.8 (C_9), 79.0 (-$\underline{C}(CH_3)_3$), 57.5 (C_1/C_5), 56.3 (rot. C_1/C_5), 53.6 (rot. C_1/C_5), 52.1 (C_1/C_5), 42.9 (C_2), 34.3 (rot. C_6/C_7), 34.0 (C_6/C_7), 29.6 (C_6/C_7), 28.8 (rot. C_6/C_7), 28.5 (rot. -$C(\underline{C}H_3)_3$), 28.2 (-$C(\underline{C}H_3)_3$).

HRMS (APCI): m/z calculated for $C_{16}H_{21}O_3NNa^+$ $[M+Na]^+$ 298.1414, found 298.1412.

IR: 2974, 2924, 2872, 2852, 1692, 1500, 1477, 1421, 1391, 1366, 1339, 1248, 1220, 1172, 1159, 1133, 1104, 1066, 1045, 1024, 1006, 956, 907, 893, 874, 850, 829, 777 cm^{-1}

Melting Point: 63.6 – 64.0 °C

SFC Chiralpak ® IC; 1500 psi, 30 °C; flow: 1.5 ml/min; from 1% to 30% MeOH in 5 min (major enantiomer t_R = 2.15 min; minor enantiomer t_R = 2.22 min)

[α]^{25}$_D$ = −93.4 (c = 0.5, $CHCl_3$).

Synthesis of 3s

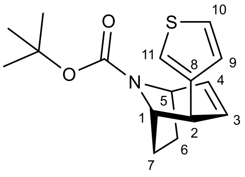

3s

The corresponding compound was prepared following General Procedure A using (±)-**1a** and **2s**. The reaction was stirred for 2 h. Purification by automated medium-pressure chromatography (hexane/EtOAc = 100/0 to 90/10) afforded the product **3s** as a white solid (27 mg, 44% yield). ^{1}H NMR integration showed a diastereomeric ratio of 19:1. SFC analysis showed an enantiomeric excess of 88%.

Further, (+)-**1a** was isolated as a white solid (19 mg, 40% yield). SFC analysis showed an enantiomeric excess of >99%.

^{1}H NMR ($CDCl_3$, 600 MHz) δ (ppm) = 7.28 – 7.26 (m, 1H, Aryl-H), 7.15 – 6.99 (m, 1H, Aryl-H), 6.99 – 6.91 (m, 1H, Aryl-H), 6.27 – 5.97 (m, 1H, C_4-H), 5.66 (ddd, *J* = 9.7, 4.2, 1.7 Hz, 1H, C_3-H), 4.62 – 4.29 (m, 2H, C_1-H, C_5-H), 3.31 (d, *J* = 4.1 Hz, 1H, C_2-H), 2.31 – 2.12 (m, 1H, C_6/C_7-H), 1.96 – 1.84 (m, 2H, C_6/C_7-H), 1.84 – 1.68 (m, 1H, C_6/C_7-H), 1.17 (s, 9H, -C(CH_3)$_3$).

^{13}C NMR ($CDCl_3$, 151 MHz) δ (ppm) = 152.6 (-C=O), 132.9 (C_4), 131.3 (rot. C_4), 127.9 (Aryl-C), 127.5 (Aryl-C), 126.7 (rot. C_3), 125.6 (C_3), 125.2 (Aryl-C), 121.8 (Aryl-C), 78.9 (-$\underline{C}$(CH_3)$_3$), 58.1 (C_1/C_5), 56.6 (rot. C_1/C_5), 53.6 (rot. C_1/C_5), 52.0 (C_1/C_5), 47.0 (C_2), 34.4 (rot. C_6/C_7), 34.0 (C_6/C_7), 29.9 (rot. C_6/C_7), 29.7 (C_6/C_7), 28.5 (rot. -C($\underline{C}H_3$)$_3$), 28.2 (-C($\underline{C}H_3$)$_3$).

HRMS (APCI): m/z calculated for $C_{16}H_{21}O_2SNNa^+$ $[M+Na]^+$ 314.1185, found 314.1183.

IR: 2959, 2923, 2852, 1677, 1634, 1475, 1432, 1390, 1363, 1344, 1311, 1292, 1274, 1251, 1229, 1179, 1163, 1132, 1110, 1041, 1007, 974, 947, 906, 891, 871, 849, 837, 763, 715, 695, 675, 652, 631 cm^{-1}

Melting Point: 78.1 - 79.4 °C

SFC Chiralpak ® IC; 1500 psi, 30 °C; flow: 1.5 ml/min; from 1% to 30% MeOH in 5 min (major enantiomer t_R = 2.14 min; minor enantiomer t_R = 2.23 min)

$[\alpha]^{25}_D$ = −106.4 (c = 0.5, $CHCl_3$).

Synthesis of 3t

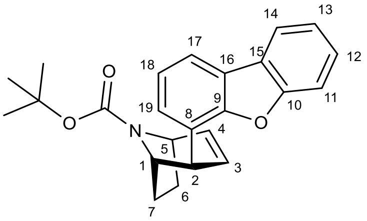

3t

The corresponding compound was prepared following General Procedure A using (±)-**1a** and **2t**. The reaction was stirred for 2 h. Purification by automated medium-pressure chromatography (hexane/EtOAc = 100/0 to 90/10) afforded the product **3t** as a white solid (16 mg, 21% yield). SFC analysis showed an enantiomeric excess of 88%.

Further, (+)-**1a** was isolated as a white solid (27 mg, 56% yield). SFC analysis showed an enantiomeric excess of 38%.

^{1}H NMR ($CDCl_3$, 600 MHz) δ (ppm) = 7.99 (d, *J* = 7.7 Hz, 1H, Aryl-H), 7.87 (d, *J* = 7.3 Hz, 1H, Aryl-H), 7.64 (d, *J* = 8.3 Hz, 1H, Aryl-H), 7.49 (t, *J* = 7.8 Hz, 1H, Aryl-H), 7.37 (t, *J* = 7.5 Hz, 1H, Aryl-H), 7.32 – 7.26 (m, 2H, Aryl-H), 6.46 – 6.19 (m, 1H, C_4-H), 5.72 (dd, *J* = 9.8, 4.2 Hz, 1H, C_3-H), 4.84 – 4.38 (m, 2H, C_1-H, C_5-H), 4.13 – 3.81 (m, 1H, C_2-H), 2.47 – 2.27 (m, 1H, C_6/C_7-H), 2.10 – 1.86 (m, 3H, C_6/C_7-H), 0.88 (s, 9H, -C(CH_3)$_3$).

^{13}C NMR ($CDCl_3$, 151 MHz) δ (ppm) = 156.2 (-C=O), 154.2 (C_9), 152.4 (C_{10}), 134.3 (C_4), 132.9 (rot. C_4), 127.3 (Aryl-C), 127.0 (Aryl-C), 126.8 (Aryl-C), 125.4 (Aryl-C), 124.7 (rot. C_3), 124.5 (C_3), 123.7 (Aryl-C), 123.0 (Aryl-C), 122.8 (Aryl-C), 120.8 (Aryl-C), 119.0 (Aryl-C), 111.8 (Aryl-C), 78.6 (-$\underline{C}$(CH_3)$_3$), 56.8 (C_1/C_5), 56.3 (rot. C_1/C_5), 53.7 (rot. C_1/C_5), 52.3 (C_1/C_5), 45.1 (C_2), 34.5 (rot. C_6/C_7), 34.1 (C_6/C_7), 30.0 (C_6/C_7), 29.3 (rot. C_6/C_7), 28.5 (rot. -C($\underline{C}H_3$)$_3$), 27.8 (-C($\underline{C}H_3$)$_3$).

HRMS (APCI): m/z calculated for $C_{24}H_{25}O_3NNa^+$ $[M+Na]^+$ 398.1727, found 398.1723.

IR: 2975, 1690, 1476, 1451, 1423, 1390, 1365, 1340, 1302, 1264, 1183, 1134, 1105, 1042, 1008, 976, 894, 845, 754, 713, 624 cm^{-1}

Melting Point: 93.8 – 94.6 °C

SFC Chiralpak ® IB; 1500 psi, 30 °C; flow: 1.5 ml/min; from 1% to 30% MeOH in 5 min (major enantiomer t_R = 2.95 min; minor enantiomer t_R = 3.18 min)

$[\alpha]^{25}_D$ = +74.5 (c = 1.0, $CHCl_3$).

1.4 Upscale and Derivatization of the product

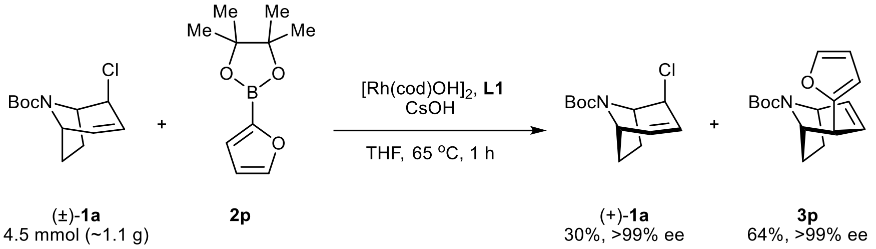

Scheme S6 Upscale Rh-catalyzed Suzuki-Miyaura cross-coupling reaction between (±)-**1a** and **2p**.

General Procedure A was used for the upscale reaction of (±)-**1a** (1.10 g, 4.5 mmol) and **2t** (2.60 g, 13.5 mmol) with reaction time of 2 h. Purification by automated medium-pressure chromatography (hexane/EtOAc = 100/0 to 90/10) afforded the product **3p** as a white solid (0.793 g, 64% yield) and enantiopure (+)-**1a** (0.326 g, 30%). SFC analysis showed an enantiomeric excess of >99% for both **3p** and (+)-**1a**.

Derivatization of product

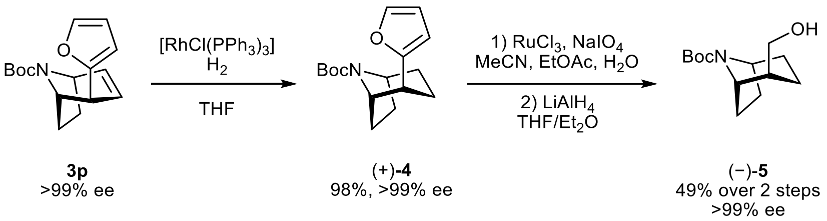

Scheme S7 Overview of derivatization of cross-coupling product **3p**.

Synthesis of (+)-4

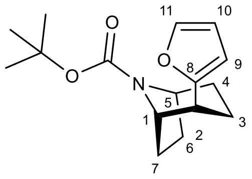

(+)-**4**

3p (0.165 g, 0.60 mmol) and [Rh(PPh$_3$)$_3$Cl] (55.5 mg, 10 mol%) was dissolved in THF (0.5 mL) and H_2 was bubbled through the solution for 3 min and the reaction was left stirring under H_2 overnight at r.t.. The crude product was loaded on to hydromatrix bulk material and purified by flash column chromatography to afford the product **of** (+)-**4** as a white solid (0.115 g, 98% yield). SFC analysis showed an enantiomeric excess of >99%.

^{1}H NMR (CDCl$_3$, 600 MHz) δ (ppm) = 7.39 – 7.31 (m, 1H, Aryl-H), 6.43 – 6.24 (m, 1H, Aryl-H), 6.23 – 6.01 (m, 1H, Aryl-H), 4.64 – 4.42 (m, 1H, C$_1$/C$_5$-H), 4.42 – 4.17 (m, 1H, C$_1$/C$_5$-H), 2.93 – 2.81 (m, 1H, C$_2$-H), 2.18 – 1.81 (m, 6H, C$_3$-H, C$_4$-H, C$_6$/C$_7$-H), 1.80 – 1.57 (m, 2H, C$_6$/C$_7$-H), 1.43 – 1.23 (s, 9H, -C(CH$_3$)$_3$).

^{13}C NMR (CDCl$_3$, 151 MHz) δ (ppm) = 157.4 (-C=O), 153.0 (Aryl-C), 140.6 (Aryl-C), 110.3 (Aryl-C), 105.5 (Aryl-C), 78.8 (-$\underline{C}$(CH$_3$)$_3$), 56.6 (C$_1$/C$_5$), 55.5 (rot. C$_1$/C$_5$), 53.9 (rot. C$_1$/C$_5$), 52.7 (C$_1$/C$_5$), 39.8 (C$_2$), 29.1 (C$_6$/C$_7$), 28.5 (C$_3$), 28.3 (-C($\underline{C}$H$_3$)$_3$), 27.9 (rot. -C($\underline{C}$H$_3$)$_3$), 27.2 (C$_6$/C$_7$), 20.0 (C$_4$).

HRMS (APCI): m/z calculated for $C_{16}H_{23}O_3NNa^+$ $[M+Na]^+$ 300.1570, found 300.1566.

IR: 2974, 1688, 1506, 1478, 1418, 1391, 1365, 1340, 1232, 1170, 1105, 1024, 953, 923, 874, 854, 759, 731, 600 cm^{-1}

Melting Point: 44.3 – 45.4 °C

SFC Chiralpak ® ID; 1500 psi, 30 °C; flow: 1.5 ml/min; from 1% to 5% MeOH in 5 min (major enantiomer t_R = 3.15 min)

$[\alpha]^{25}_D$ = +41.0 (c = 1.0, $CHCl_3$).

Synthesis of (−)-5

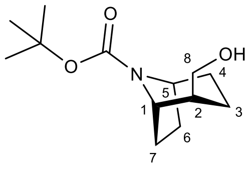

(−)-**5**

$RuCl_3$ (10.5 mg, 10 mol%) and $NaIO_4$ (1.62 g, 7.56 mmol) was dissolved in H_2O (4.0 mL), EtOAc (2.0 mL) and MeCN (0.40 mL) and stirred for 30 min until the mixture turned orange. A solution of (+)-**4** (0.111 g, 0.40 mmol) in DCM (2.0 mL) was added dropwise at 0 °C (each addition was made after the colour of the reaction mixture turned back orange). After stirring the mixture for 2 h, brine (5 mL) was added and the aqueous layer was extracted with EtOAc (10 mL × 3). The organic layer was dried over $MgSO_4$ and concentrated *in vacuo*. The mixture was purified *via* flash column chromatography (DCM/MeOH/AcOH = 94/5/1), then concentrated *in vacuo* to afford a black solid which was directly used in the next step. The black solid was dissolved in THF/Et_2O (1.3 mL / 0.86 mL) and added dropwise to solution of $LiAlH_4$ (3.0 equiv., 1.3 mL (1 M in THF)) in Et_2O (0.86 mL) at 0 °C. The reaction mixture was slowly allowed to reach r.t.. After 4 h, EtOAc (2.0 mL) and potassium sodium tartrate solution (sat.aq., 8 mL) were added and stirring was continued overnight. The reaction mixture was extracted with EtOAc (10 mL × 3) and dried over $MgSO_4$ and concentrated *in vacuo*. Purification by flash column chromatography afforded the product as a colourless oil (47 mg, 49 % yield). SFC analysis showed an enantiomeric excess of >99%.

^{1}H NMR ($CDCl_3$, 600 MHz) δ (ppm) = 4.47 – 3.97 (m, 2H, C_1-H, C_5-H), 3.57 – 3.27 (m, 2H, C_8-H), 2.13 – 2.03 (m, 1H, $C_4/C_6/C_7$-H), 2.03 – 1.91 (m, 1H, $C_4/C_6/C_7$-H), 1.90 – 1.78 (m, 2H, C_2-H, $C_4/C_6/C_7$-H), 1.73 – 1.62 (m, 3H, $C_4/C_6/C_7$-H), 1.47 (s, 9H, -$C(CH_3)_3$), 1.35 – 1.23 (m, 1H, C_3-H), 1.23 – 1.10 (m, 1H, C_3-H).

^{13}C NMR ($CDCl_3$, 151 MHz) δ (ppm) = 154.5 (-C=O), 79.8 (-$\underline{C}(CH_3)_3$), 63.1 (rot. C_8), 62.5 (C_8), 54.3 (C_1/C_5), 53.6 (rot. C_1/C_5), 52.4 (C_1/C_5), 42.4 (rot. C_2), 42.0 (C_2), 28.6 (-$C(\underline{C}H_3)_3$), 28.0 ($C_4/C_6/C_7$), 27.8 ($C_4/C_6/C_7$), 27.6 ($C_4/C_6/C_7$), 18.4 (C_3).

HRMS (APCI): m/z calculated for $C_{13}H_{23}O_3NNa^+$ $[M+Na]^+$ 264.1570, found 264.1567.

IR: 3441, 2933, 1690, 1667, 1476, 1422, 1365, 1322, 1241, 1164, 1112, 1042, 986, 959, 942, 920, 869, 763 cm^{-1}

SFC Chiralpak ® IG; 1500 psi, 30 °C; flow: 1.5 ml/min; from 1% to 20% MeOH in 5 min (major enantiomer t_R = 3.62 min; minor enantiomer t_R = 3.21 min)

$[\alpha]^{25}_D$ = −1.3 (c = 1.0, $CHCl_3$).

Derivatization of Enantiopure Allyl Chloride

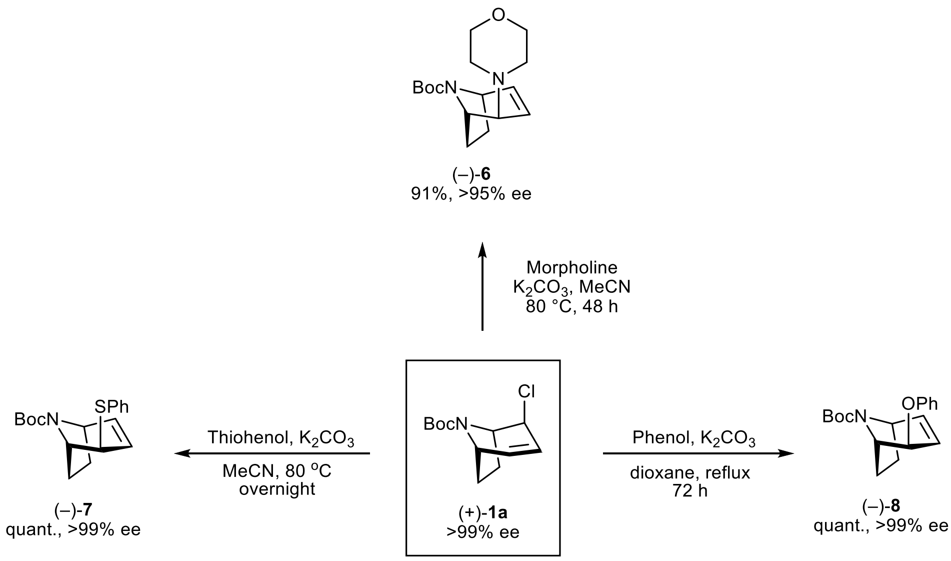

Scheme S8 Overview of derivatization of resolved enantiopure allyl chloride (+)-**1a**.

Synthesis of (−)-6

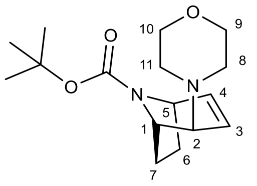

(−)-**6**

According to a previous procedure by the group with modified conditions,[7] (+)-**1a** (57.6 mg, 0.24 mol), K_2CO_3 (98.1 mg, 0.71 mmol) was dissolved in MeCN (2.4 mL) and morpholine (29 µL, 0.34 mmol) was added. The reaction mixture was heated to 80 °C and stirred for 48 h before cooling to r.t. and dry loading onto silica. Purification by automated medium-pressure chromatography (hexane/acetone = 100/0 to 60/40) afforded the product (−)-**6** as a white solid (mg, 91% yield). SFC analysis showed an enantiomeric excess of >95% due to inability to find a gradient to separate two peaks.

^{1}H NMR ($CDCl_3$, 600 MHz) δ (ppm) = 6.16 (s, 1H, C_4-H), 5.52 (s, 1H, C_3-H), 4.74 – 4.36 (m, 2H, C_1-H, C_5-H), 3.75 – 3.57 (m, 4H, C_8-H, C_{11}-H), 2.75 (ddd, J = 14.8, 7.3, 4.3 Hz, 3H, C_2-H, C_9/C_{10}-H), 2.68 – 2.57 (m, 2H, C_9/C_{10}-H), 2.09 – 1.96 (m, 1H, C_6/C_7-H), 1.73 (d, J = 9.5 Hz, 3H, C_6/C_7-H), 1.53 – 1.28 (m, 9H, -$C(CH_3)_3$).

^{13}C NMR ($CDCl_3$, 151 MHz) δ (ppm) = 152.9 (-C=O), 135.1 (C_4), 134.5 (rot. C_4), 124.7 (rot. C_3), 124.3 (C_3), 79.8 (-$\underline{C}(CH_3)_3$), 67.8 (C_8, C_{11}), 67.6 (C_2), 53.5 (rot. C_1/C_5), 52.4 (C_1/C_5), 52.2 (C_1/C_5), 51.2 (rot. C_1/C_5), 49.7 (C_9, C_{10}), 28.7 (-$C(\underline{C}H_3)_3$), 28.0 (C_6/C_7), 27.4 (C_6/C_7).

HRMS (APCI): m/z calculated for $C_{16}H_{27}O_3N_2^+$ $[M+H]^+$ 295.2016, found 295.2016.

IR: 2955, 2920, 2851, 1693, 1477, 1452, 1420, 1392, 1366, 1338, 1296, 1253, 1228, 1166, 1135, 1116, 1100, 1069, 1043, 1004, 979, 957, 924, 905, 893, 869, 830, 770, 712, 697, 662, 647, 619, 607 cm^{-1}

Melting Point: 89.6 – 90.7 °C

SFC Chiralpak ® IG; 1500 psi, 30 °C; flow: 1.5 ml/min; from 1% to 20% MeOH in 5 min (major enantiomer t_R = 3.01 min).

[α]^{25}$_D$ = −34.3 (c = 1.0, $CHCl_3$).

Synthesis of (−)-7

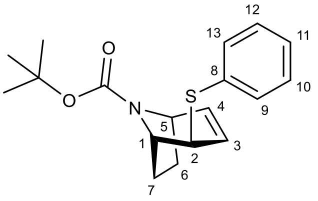

(−)-**7**

According to a previous procedure by the group with modified conditions,[7] (+)-**1a** (37.4 mg, 0.125 mmol), K_2CO_3 (0.11 g, 0.25 mmol) was dissolved in MeCN (1.6 mL) and thiophenol (38.0 µL) was added (0.04 mL, 0.375 mmol). Then, the reaction mixture was heated at 80 °C overnight before cooling to r.t. and dry loading onto silica. Purification by automated medium-pressure chromatography (hexane/EtOAc = 100/0 to 90/10) afforded the product (−)-**7** as a white solid (39 mg, quantitative yield). SFC analysis showed an enantiomeric excess of >99%.

^{1}H NMR ($CDCl_3$, 600 MHz) δ (ppm) = 7.64 – 7.40 (m, 2H, Aryl-H), 7.30 (t, J = 7.6 Hz, 2H, Aryl-H), 7.24 (t, J = 7.4 Hz, 1H, Aryl-H), 6.14 – 5.97 (m, 1H, C_4-H), 5.69 – 5.50 (m, 1H, C_3-H), 4.63 – 4.42 (m, 2H, C_1-H, C_5-H), 3.62 – 3.43 (m, 1H, C_2-H), 2.18 – 2.02 (m, 1H, C_6/C_7-H), 1.90 – 1.75 (m, 2H, C_6/C_7-H), 1.62 – 1.55 (m, 1H, C_6/C_7-H), 1.46 (s, 9H, -C(CH_3)$_3$).

^{13}C NMR ($CDCl_3$, 151 MHz) δ (ppm) = 153.6 (-C=O), 136.0 (C_4), 135.3 (rot. C_4), 134.1 (C_8), 133.4 (Aryl-C), 131.4 (Aryl-C), 129.1 (Aryl-C), 127.0 (Aryl-C), 124.2 (rot. C_3), 123.5 (C_3), 79.8 (-<u>C</u>(CH_3)$_3$), 56.2 (rot. C_1/C_5), 54.0 (C_2), 53.1 (rot. C_1/C_5), 52.4 (C_1/C_5), 33.4 (C_6/C_7), 29.1 (C_6/C_7), 28.5 (-C(<u>C</u>H_3)$_3$), 28.5 (rot. -C(<u>C</u>H_3)$_3$).

HRMS (APCI): m/z calculated for $C_{18}H_{23}O_2SNNa^+$ [M+Na]$^+$ 340.1342, found 340.1341.

Melting Point: 76.3 – 77.8 °C

IR: 2974, 2929, 2870, 2360, 1693, 1584, 1478, 1420, 1391, 1366, 1338, 1298, 1250, 1225, 1168, 1102, 1065, 1042, 1026, 1003, 966, 892, 872, 835, 785, 742, 693, 650, 627 cm^{-1}

SFC Chiralpak ® IA; 1500 psi, 30 °C; flow: 1.5 ml/min; from 1% to 30% MeOH in 5 min (major enantiomer t_R = 2.59 min; minor enantiomer t_R = 2.47 min)

[α]^{25}$_D$ = −57.9 (c = 1.0, $CHCl_3$).

Synthesis of (−)-8

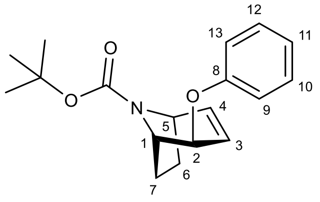

(−)-**8**

According to a previous procedure by the group with modified conditions,[7] (+)-**1a** (32.3 mg, 0.13 mmol), K_2CO_3 (72.3 mg, 0.52 mmol) and phenol (21.8 mg, 0.30 mmol) was dissolved in 1,4-dioxane (2 mL) and the reaction mixture was heated to reflux for 72 h before cooling to r.t. Acetyl chloride (0.04 mL) was added dropwise and the reaction was wash with brine followed by extraction with EtOAc (10 mL × 3). The crude product was loaded onto silica and purification by automated medium-pressure chromatography (hexane/EtOAc = 100/0 to 90/10) afforded the product (−)-**8** as a white solid (38 mg, quantitative yield). SFC analysis showed an enantiomeric excess of >99%.

^{1}H NMR ($CDCl_3$, 600 MHz) δ (ppm) = 7.32 – 7.26 (m, 2H, Aryl-H), 7.05 – 6.87 (m, 3H, Aryl-H), 6.33 (s, 1H, C_4-H), 5.84 – 5.68 (m, 1H, C_3-H), 4.68 (d, J = 7.8 Hz, 1H, C_1/C_5-H), 4.64 – 4.51 (m, 1H, C_1/C_5-H), 4.30 (d, J = 4.4 Hz, 1H, C_2), 2.23 – 2.07 (m, 1H, C_6/C_7-H), 1.87 (s, 1H, C_6/C_7-H), 1.71 (ddd, J = 11.5, 8.9, 2.4 Hz, 1H, C_6/C_7-H), 1.52 – 1.24 (m, 10H, -C(CH_3)$_3$, C_6/C_7-H).

^{13}C NMR ($CDCl_3$, 151 MHz) δ (ppm) = 157.7 (-C=O), 153.4 (C_8), 137.1 (C_4), 129.6 (Aryl-C), 122.5 (Aryl-C), 121.1 (Aryl-C), 117.0 (rot. C_3), 115.7 (C_3), 79.5 (-$\underline{C}$(CH_3)$_3$), 54.3 (C_2), 53.6 (C_1/C_5), 51.9 (C_1/C_5), 30.8 (C_6/C_7), 29.8 (rot. C_6/C_7), 28.5 (rot. -C($\underline{C}H_3$)$_3$), 28.3 (-C($\underline{C}H_3$)$_3$), 24.82 (C_6/C_7).

HRMS (APCI): m/z calculated for $C_{18}H_{23}O_3NNa^+$ $[M+Na]^+$ 324.1570, found 324.1570.

Melting Point: 72.5 – 73.4 °C

IR: 2975, 2360, 1693, 1596, 1493, 1420, 1390, 1365, 1347, 1319, 1292, 1229, 1174, 1105, 1072, 1050, 1027, 1010, 990, 911, 872, 840, 821, 753, 718, 693, 608 cm^{-1}

SFC Chiralpak ® IG; 1500 psi, 30 °C; flow: 1.5 ml/min; from 1% to 30% MeOH in 5 min (major enantiomer t_R = 2.85 min; minor enantiomer t_R = 3.02 min)
$[\alpha]^{25}_D$ = −26.4 (c = 1.0, $CHCl_3$).

1.5 Calculation of Apparent *S*-factors

The ee of the remaining allyl chloride (+)-**1a** and product **3a** – **3t** are determined by Super Fluid Chromatography (SFC).

Two different conversion values of **1a** were used for calculation of apparent *s*-factors: Conversion 1 (C1) was determined by (1− isolated yield of **1a** after reaction), Conversion 2 (C2) was determined by (1− isolated yield of product after reaction) taking into account the competitive hydrolysis of **1a**.

The apparent *s*-factors were calculated using formula (1) below (C1/C2: Conversion; ee: ee of **1a**).[9]

$$s = \frac{\ln[(1-C)(1-ee)]}{\ln[(1-C)(1+ee)]} \qquad (1)$$

The calculated apparent *s*-factors are shown below in Table S3.

Some of the apparent s-factor values give what might be unexpected values, likely due to one or both of the following reasons:

1. Competitive hydrolysis resulted in an inaccurate conversion during calculation, as consumption of **1a** did not all result in product formation;

2. The Rh-catalyzed cross-coupling reaction does not proceed like classical kinetic resolutions, where one enantiomer of the starting material is converted to product much faster than the other enantiomer so that the less reactive enantiomer of the substrate and the product are both highly enantioenriched. In our case, there is potentially equilibration of intermediates arising from both enantiomers of **1a**, and a partial DYKAT-like mechanism. Therefore, the S-factor cannot be used to meaningfully measure the efficiency of the observed kinetic resolution, and so the values given here are the 'apparent s-factor'.

Table S3. Yields and ee of products and calculated conversion and *s*-factor.

Entry	Product	Product yield/%	Product ee/%	% of SM recovered	**1a** ee/%	C1/%	C2/%	Apparent s-factor 1	Apparent s-factor 2
1	**3a**	50	95	39	>99	61	50	21.88	1057.01
2	**3b**	44	96	30	>99	70	56	11.26	40.85
3	**3c**	32	94	37	>99	63	68	18.29	12.73
4	**3d**	51	95	31	>99	69	49	11.96	-356.90
5	**3e**	44	94	21	>99	79	56	7.07	40.85
6	**3f**	49	98	38	>99	62	51	19.94	210.92
7	**3g**	47	96	25	>99	75	53	8.58	80.14
8	**3h**	39	96	35	>99	65	61	15.64	21.88
9	**3i**	22	96	66	59	34	78	-27.11	2.29
10	**3j**	23	97	46	39	54	77	2.84	1.72
11	**3k**	19	98	65	31	35	81	4.99	1.46
12	**3l**	59	96	27	>99	73	41	9.52	-31.98
13	**3m**	38	96	12	>99	88	62	4.70	19.94
14	**3n**	49	97	46	>99	54	51	60.88	210.92
15	**3o**	46	97	36	>99	64	54	16.87	60.88
16	**3p**	56	97	28	>99	72	44	10.05	-47.87
17	**3q**	42	97	26	>99	74	58	9.03	30.51
18	**3r**	15	98	65	26	35	85	3.67	1.32
19	**3s**	44	88	40	>99	60	56	24.20	40.85
20	**3t**	21	88	56	38	44	79	4.10	1.65

2. References

1. Friese, F. W.; Studer, A., Deoxygenative Borylation of Secondary and Tertiary Alcohols. *Angew. Chem. Int. Ed.* **2019,** *58*, 9561-9564.

2. Nagase, T.; Takahashi, T.; Sasaki, T.; Nagumo, A.; Shimamura, K.; Miyamoto, Y.; Kitazawa, H.; Kanesaka, M.; Yoshimoto, R.; Aragane, K., Synthesis and biological evaluation of a novel 3-sulfonyl-8-azabicyclo [3.2. 1] octane class of long chain fatty acid elongase 6 (ELOVL6) inhibitors. *J. Med. Chem.* **2009,** *52*, 4111-4114.

3. Dallanoce, C.; Frigerio, F.; Martelli, G.; Grazioso, G.; Matera, C.; Pomè, D. Y.; Pucci, L.; Clementi, F.; Gotti, C.; De Amici, M., Novel tricyclic Δ2-isoxazoline and 3-oxo-2-methyl-isoxazolidine derivatives: Synthesis and binding affinity at neuronal nicotinic acetylcholine receptor subtypes. *Bioorg. Med. Chem.* **2010,** *18*, 4498-4508.

4. Aggarwal, V. K.; Astle, C. J.; Rogers-Evans, M., A concise asymmetric route to the bridged bicyclic tropane alkaloid ferruginine using enyne ring-closing metathesis. *Org. Lett.* **2004,** *6*, 1469-1471.

5. Chavan, S. P.; Dumare, N. B.; Pawar, K. P., A novel, concise and efficient protocol for non-natural piperidine compounds. *RSC Adv.* **2014,** *4*, 32594-32598.

6. Schäfer, P.; Palacin, T.; Sidera, M.; Fletcher, S. P., Asymmetric Suzuki-Miyaura coupling of heterocycles via Rhodium-catalysed allylic arylation of racemates. *Nat. Comm.* **2017,** *8*, 15762.

7. Karabiyikoglu, S.; Brethomé, A. V.; Palacin, T.; Paton, R. S.; Fletcher, S. P., Enantiomerically enriched tetrahydropyridine allyl chlorides. *Chem. Sci.* **2020,** *11*, 4125-4130.

8. González, J.; van Dijk, L.; Goetzke, F. W.; Fletcher, S. P., Highly enantioselective rhodium-catalyzed cross-coupling of boronic acids and racemic allyl halides. *Nat. Protoc.* **2019,** *14*, 2972-2985.

9. Vedejs, E.; Jure, M., Efficiency in Nonenzymatic Kinetic Resolution. *Angew. Chem. Int. Ed.* **2005,** *44*, 3974-4001.

10. Palatinus, L.; Chapuis, G., SUPERFLIP–a computer program for the solution of crystal structures by charge flipping in arbitrary dimensions. *J. Appl. Crystallogr.* **2007,** *40*, 786-790.

11. Parois, P.; Cooper, R. I.; Thompson, A. L., Crystal structures of increasingly large molecules: meeting the challenges with CRYSTALS software. *Chem. Cent. J.* **2015,** *9*, 30-30.

12. Cooper, R. I.; Thompson, A. L.; Watkin, D. J., CRYSTALS enhancements: dealing with hydrogen atoms in refinement. *J. Appl. Crystallogr.* **2010,** *43*, 1100-1107.

3. NMR Spectra

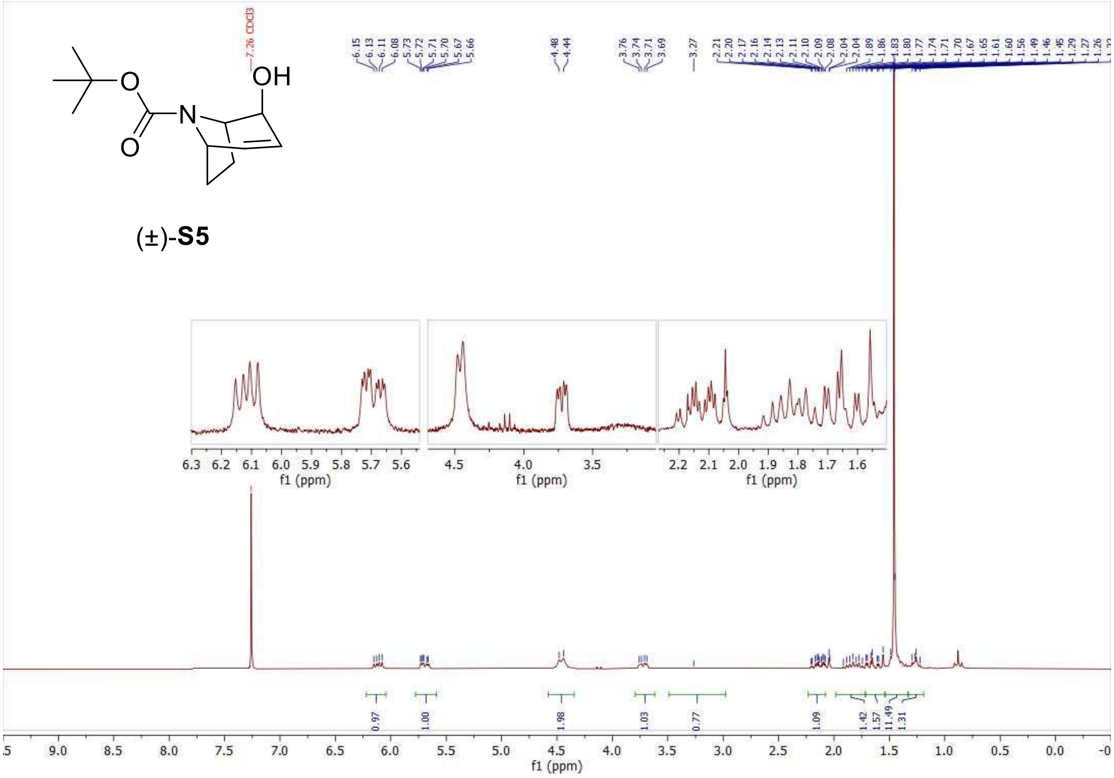

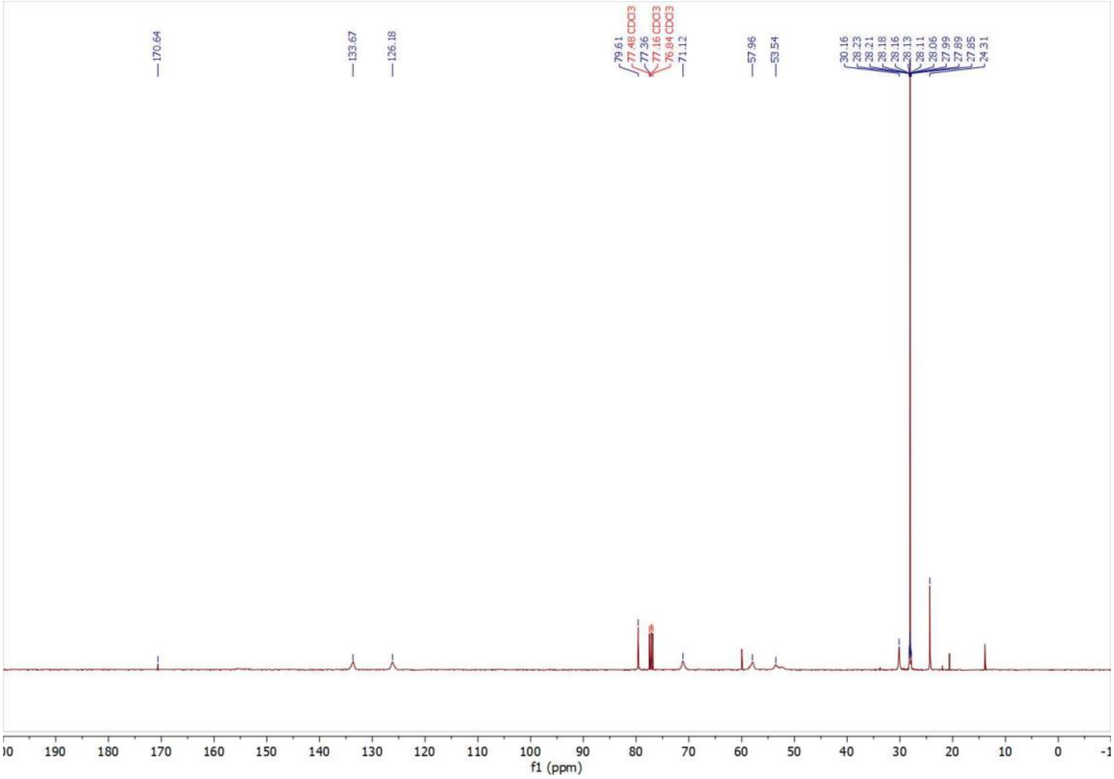

Figure S2. ^{1}H NMR (400 MHz, $CDCl_3$, top) and ^{13}C NMR (101 MHz, $CDCl_3$, bottom) spectra of **(±)-S5** recorded at 298 K.

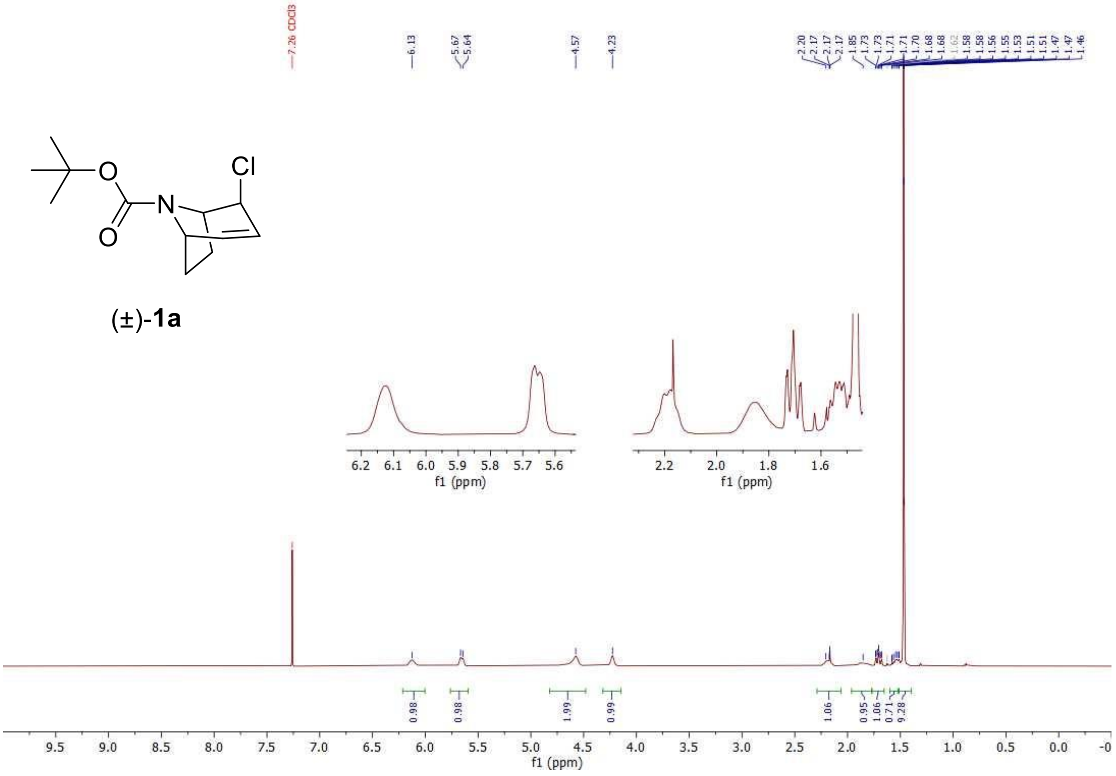

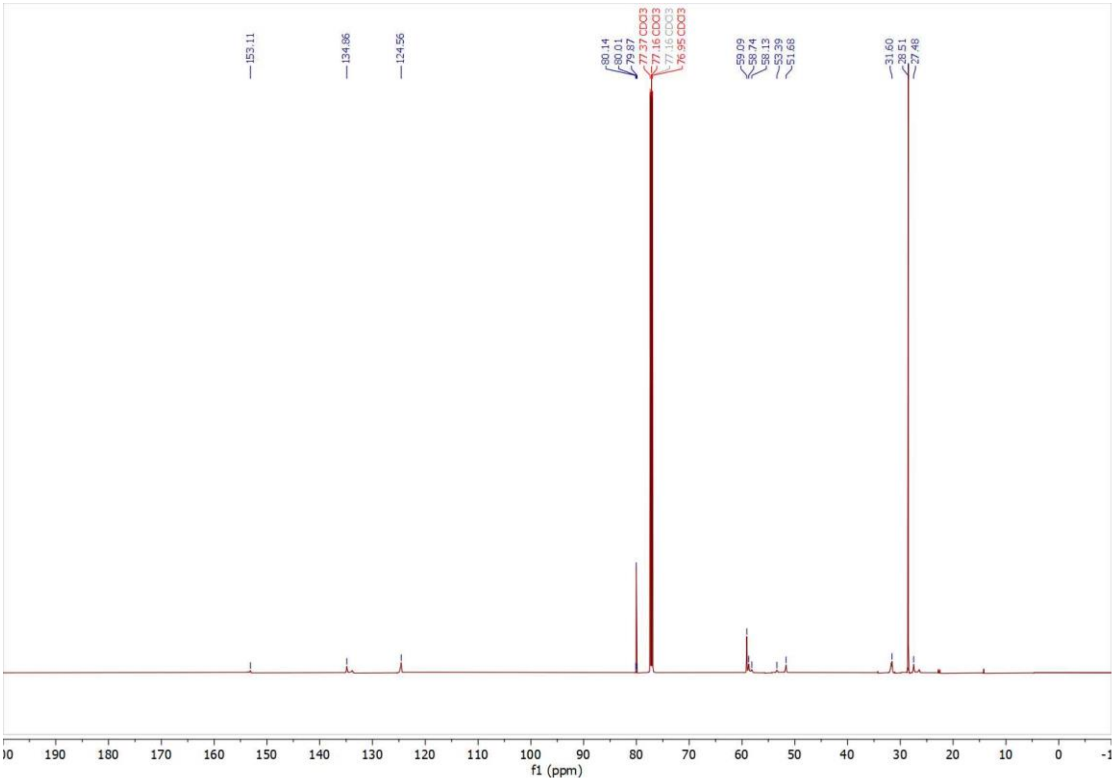

Figure S3. ^{1}H NMR (400 MHz, $CDCl_3$, top) and ^{13}C NMR (151 MHz, $CDCl_3$, bottom) spectra of **(±)-1a** recorded at 298 K.

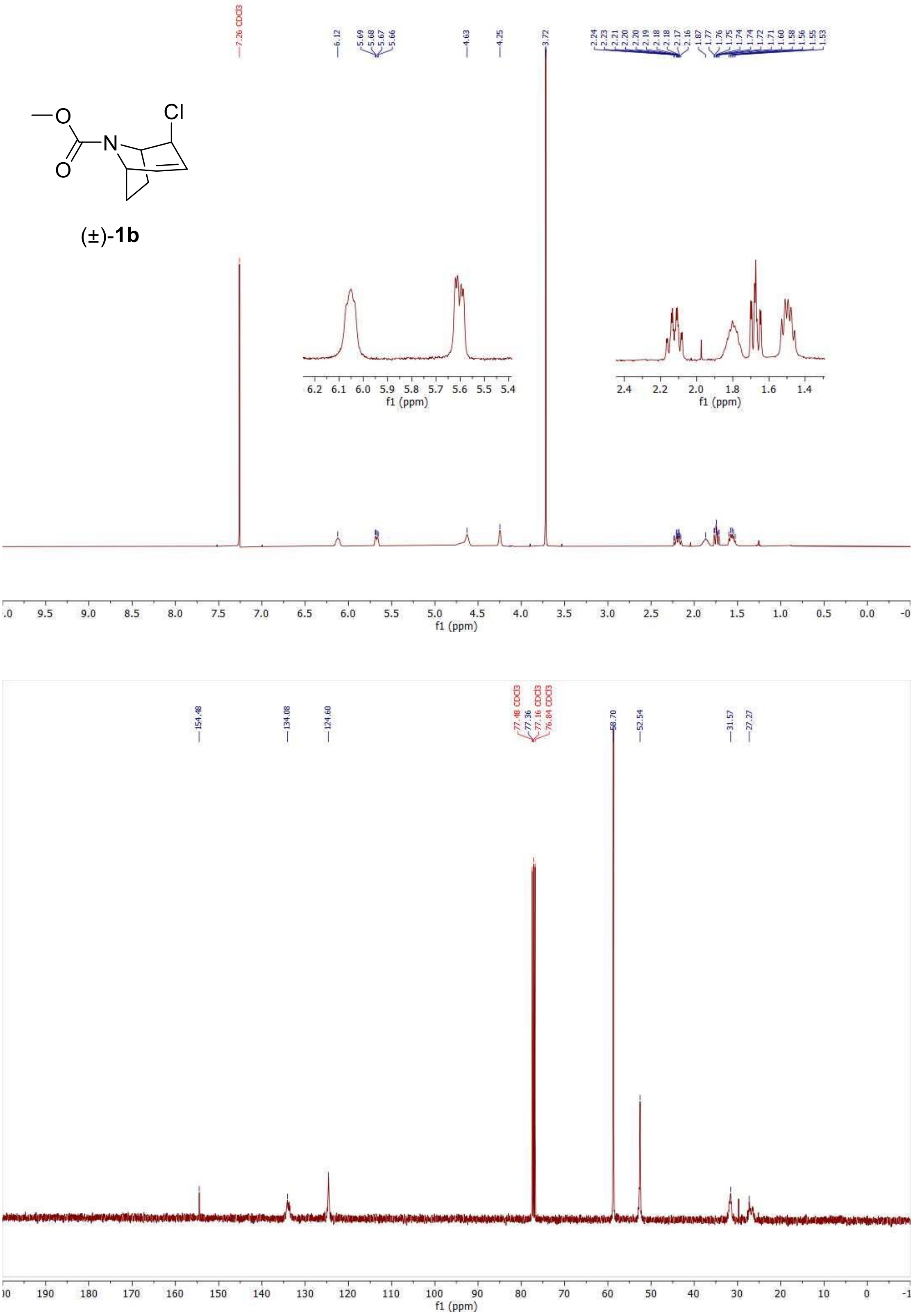

Figure S4. ^{1}H NMR (400 MHz, $CDCl_3$, top) and ^{13}C NMR (101 MHz, $CDCl_3$, bottom) spectra of **(±)-1b** recorded at 298 K.

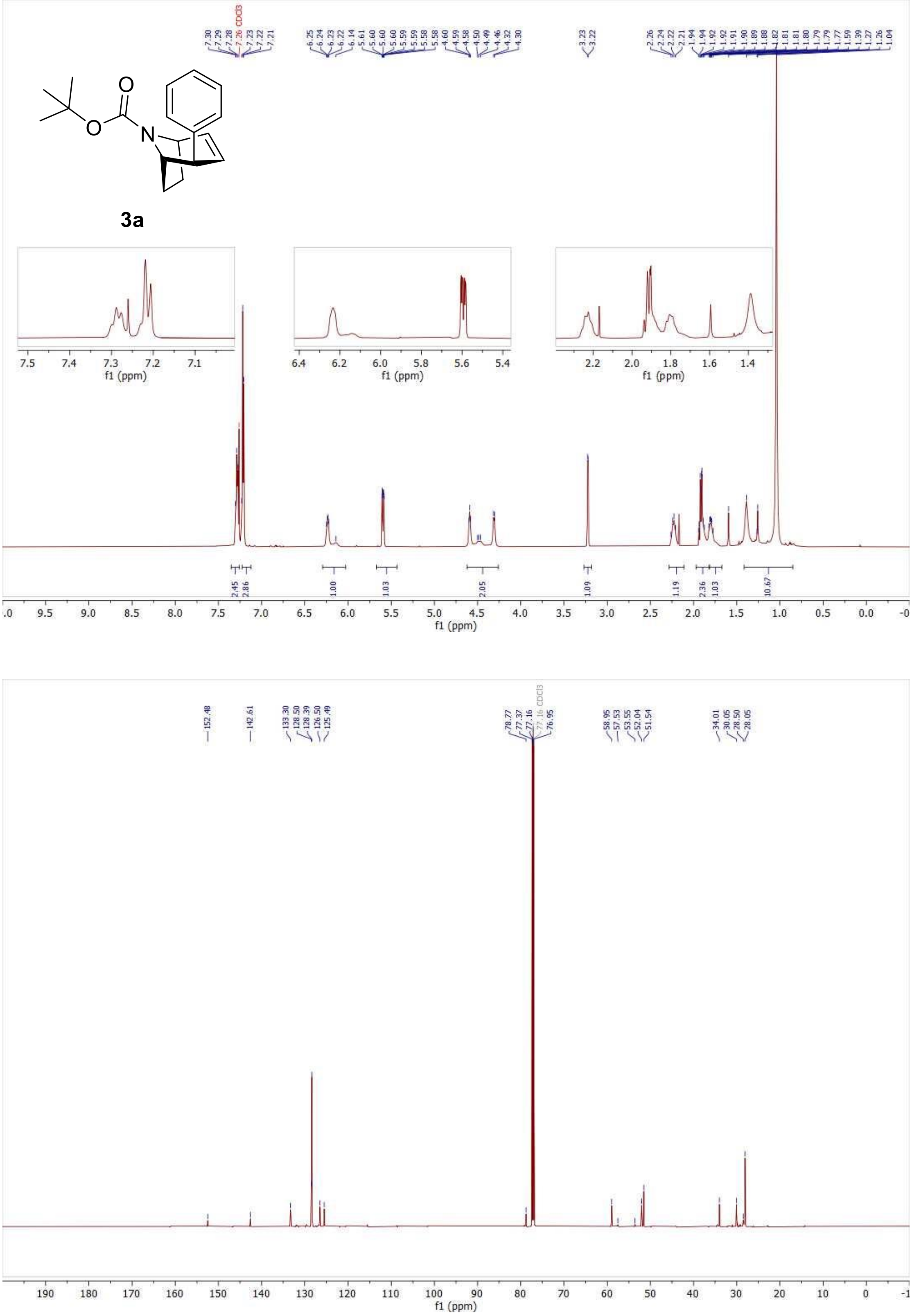

Figure S5. ^{1}H NMR (600 MHz, $CDCl_3$, top) and ^{13}C NMR (151 MHz, $CDCl_3$, bottom) spectra of **3a** recorded at 298 K.

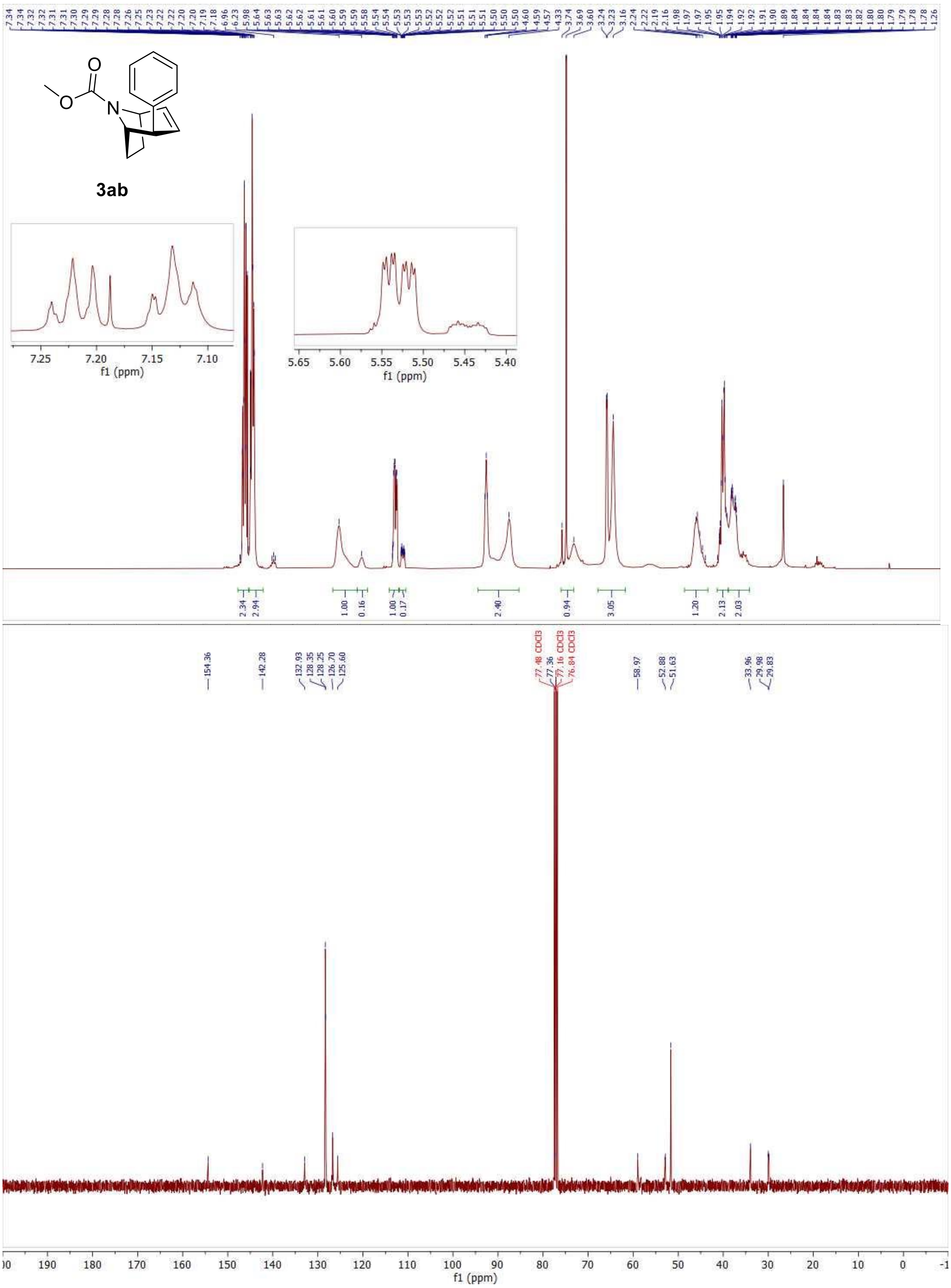

Figure S6. ^{1}H NMR (400 MHz, $CDCl_3$, top) and ^{13}C NMR (101 MHz, $CDCl_3$, bottom) spectra of **3ab** recorded at 298 K.

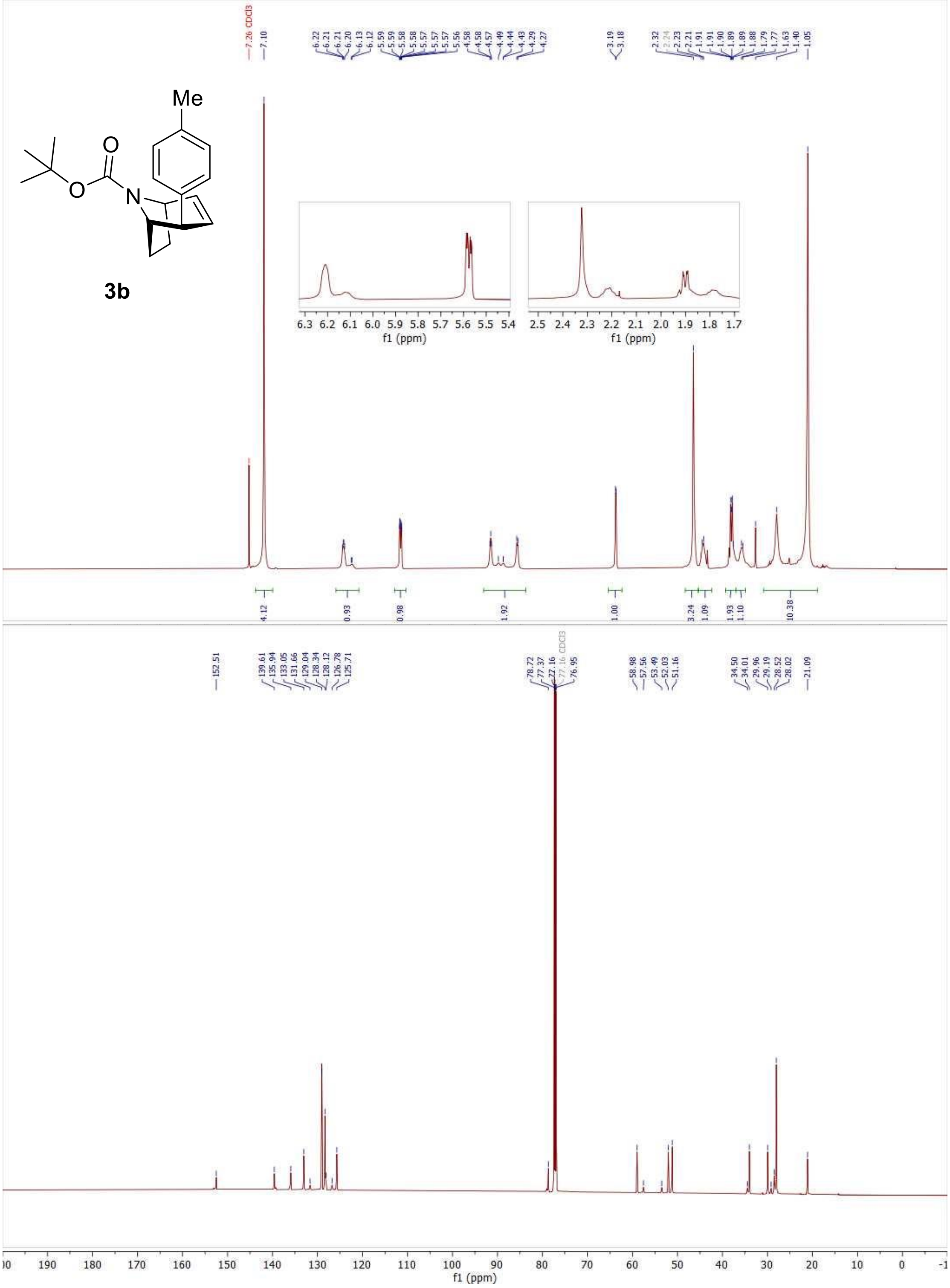

Figure S7. ^{1}H NMR (600 MHz, $CDCl_3$, top) and ^{13}C NMR (151 MHz, $CDCl_3$, bottom) spectra of **3b** recorded at 298 K.

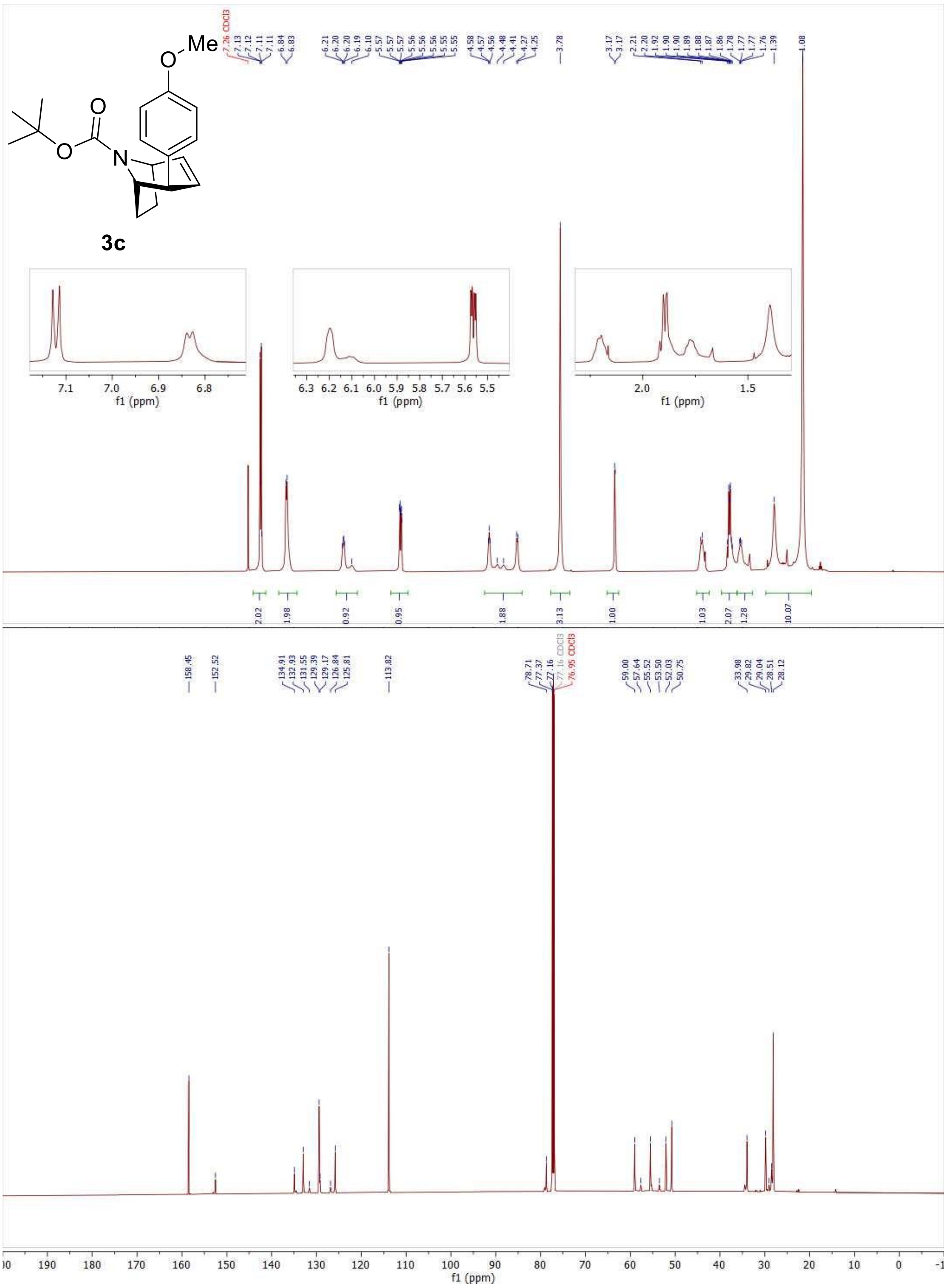

Figure S8. ^{1}H NMR (600 MHz, $CDCl_3$, top) and ^{13}C NMR (151 MHz, $CDCl_3$, bottom) spectra of **3c** recorded at 298 K.

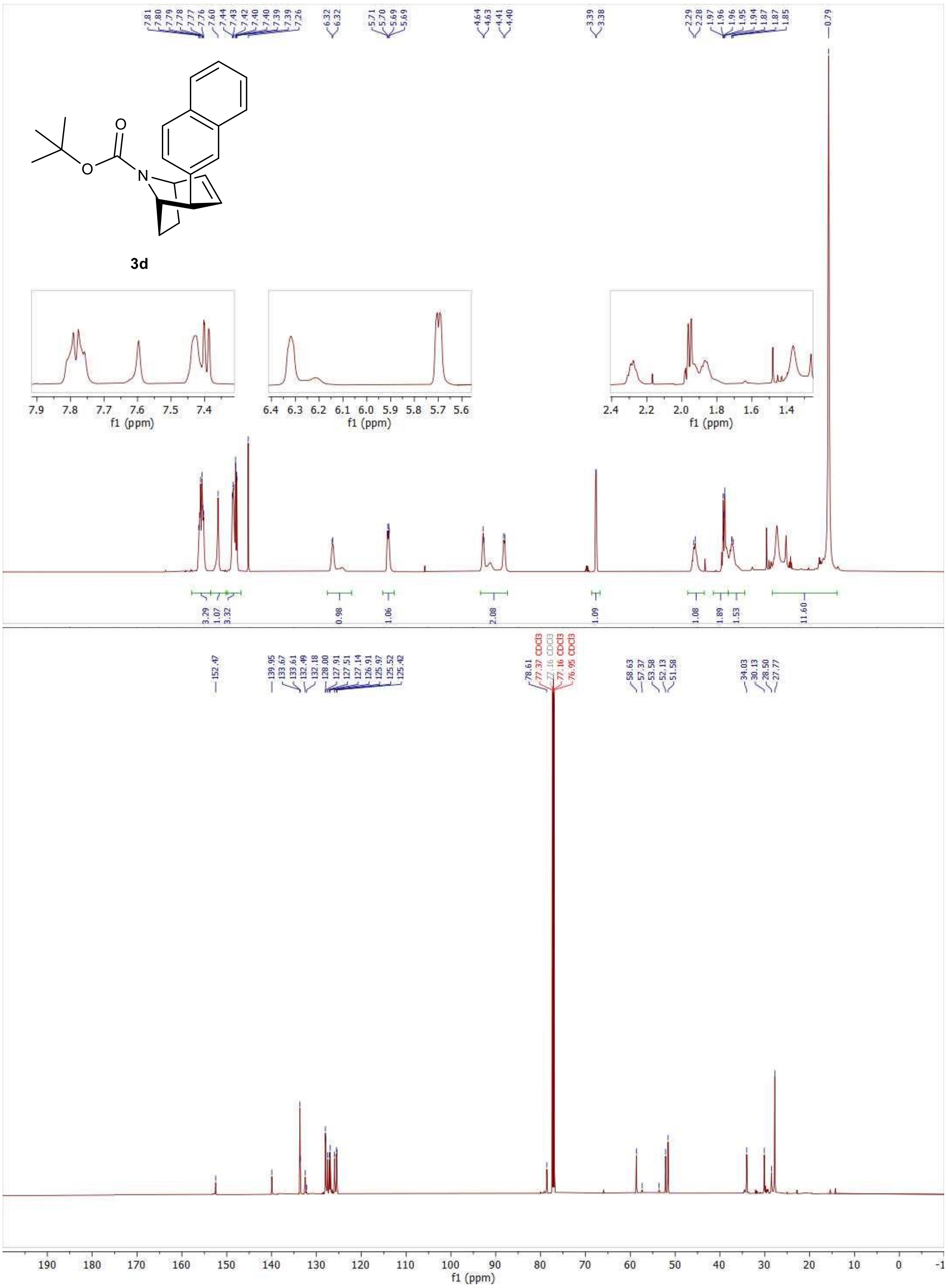

Figure S9. ^{1}H NMR (600 MHz, $CDCl_3$, top) and ^{13}C NMR (151 MHz, $CDCl_3$, bottom) spectra of **3d** recorded at 298 K.

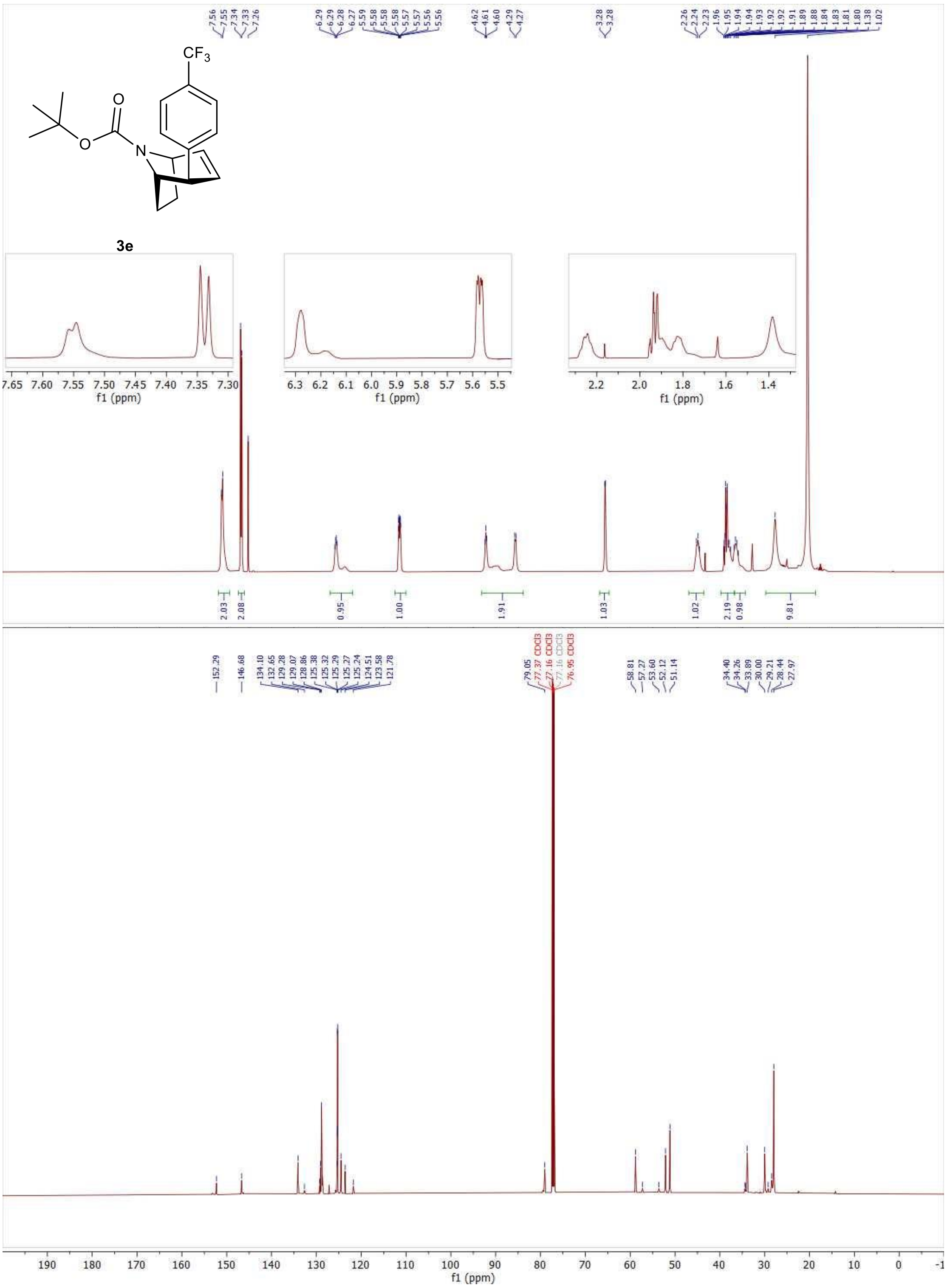

Figure S10. ^{1}H NMR (600 MHz, $CDCl_3$, top) and ^{13}C NMR (151 MHz, $CDCl_3$, bottom) spectra of **3e** recorded at 298 K.

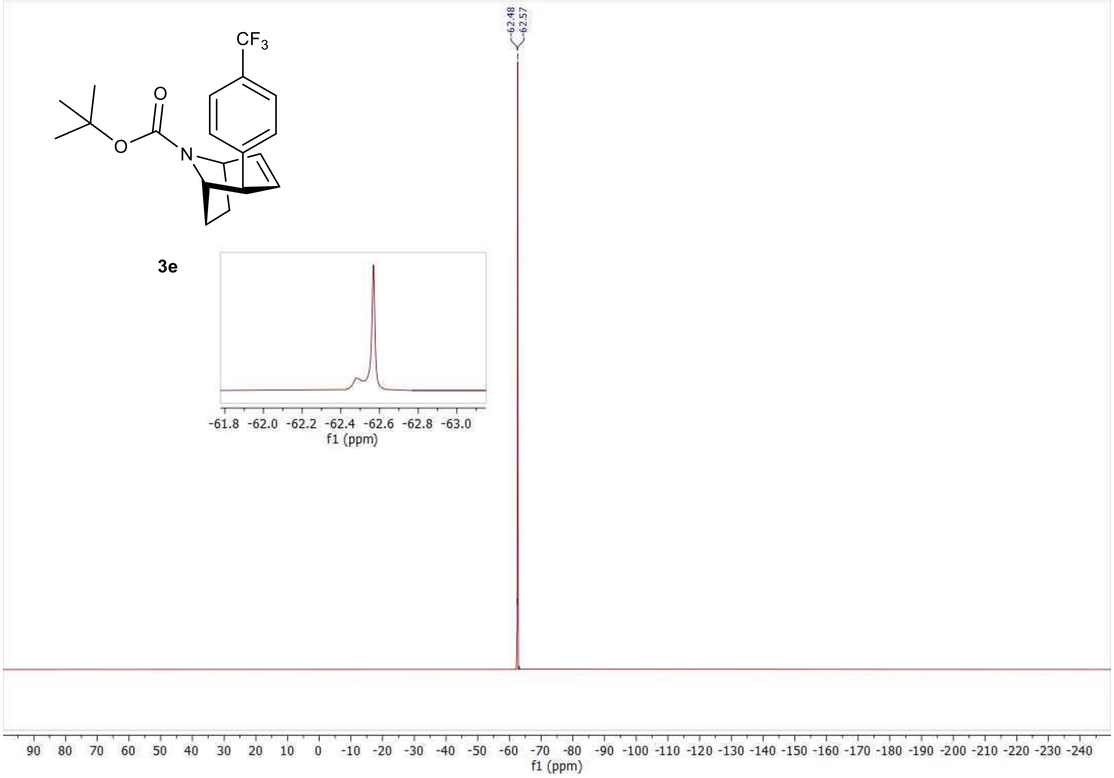

Figure S10-1. ^{19}F NMR (376 MHz, $CDCl_3$) of **3e** recorded at 298 K.

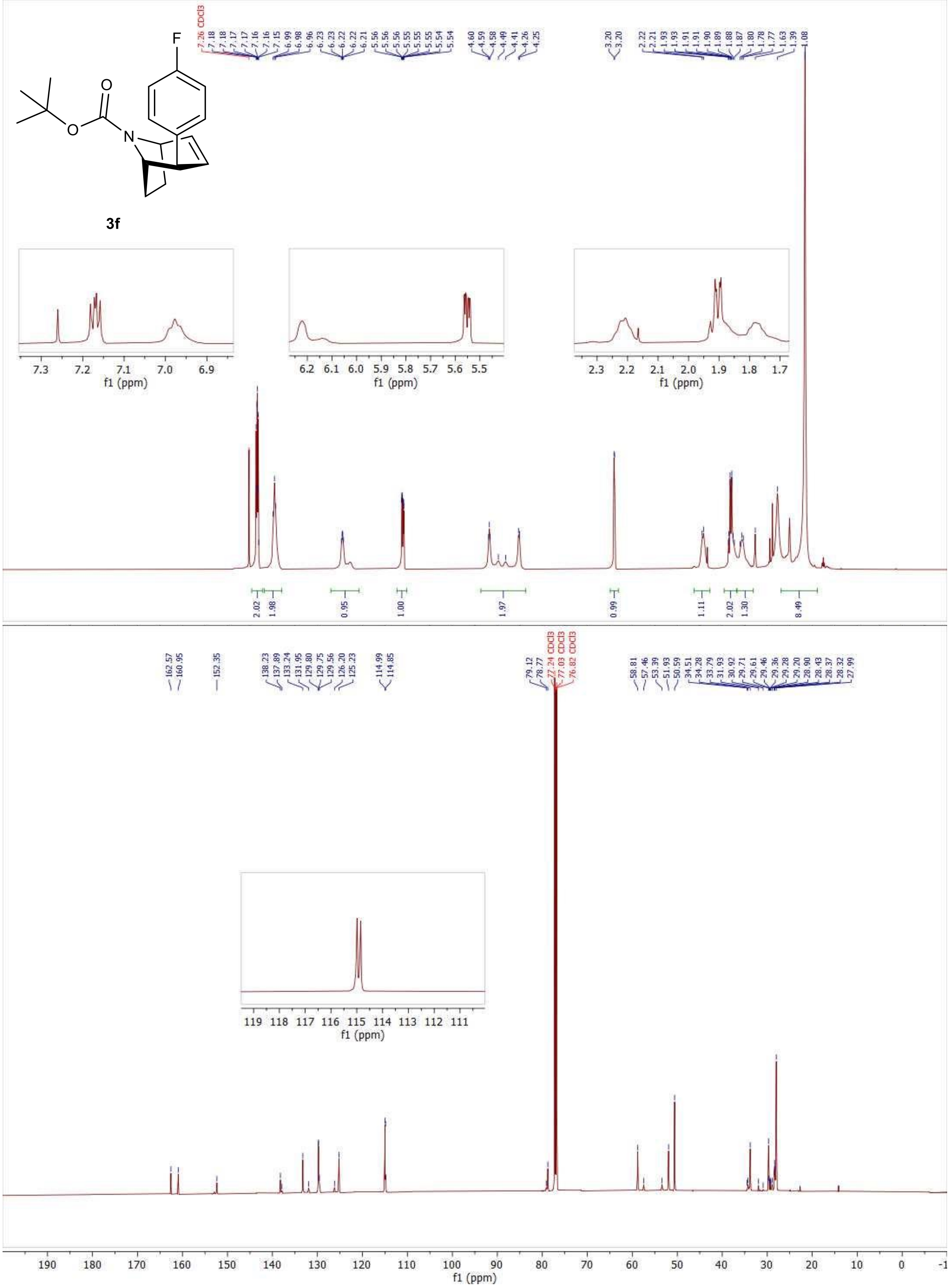

Figure S11. ^{1}H NMR (600 MHz, $CDCl_3$, top) and ^{13}C NMR (151 MHz, $CDCl_3$, bottom) spectra of **3f** recorded at 298 K.

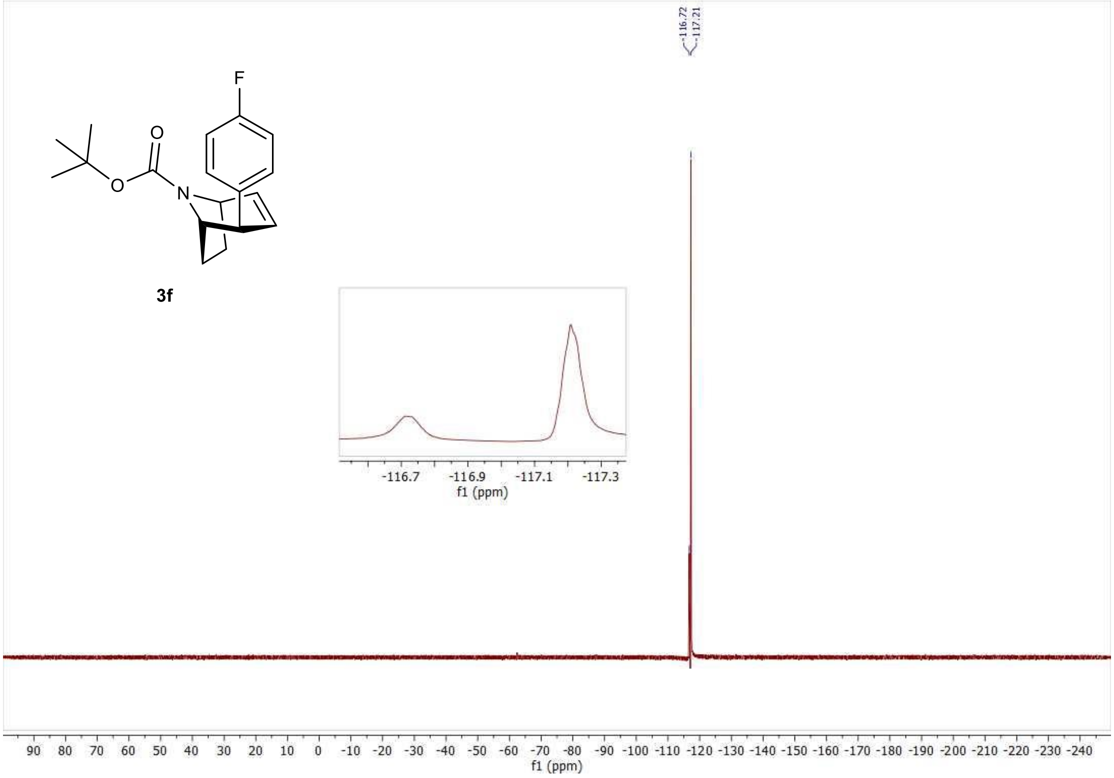

Figure S11-1. ^{19}F NMR (376 MHz, $CDCl_3$) spectra of **3f** recorded at 298 K.

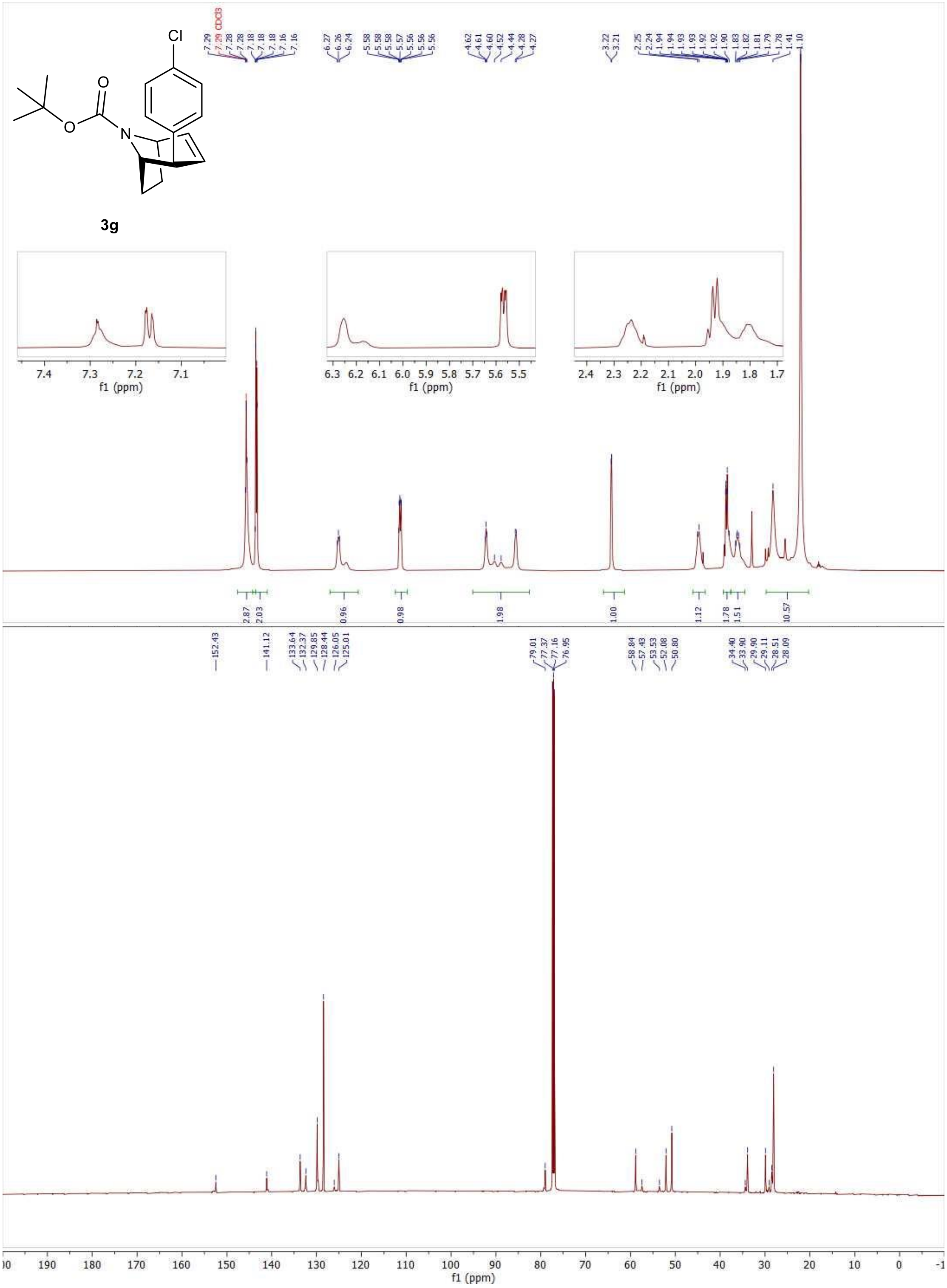

Figure S12. ^{1}H NMR (600 MHz, $CDCl_3$, top) and ^{13}C NMR (151 MHz, $CDCl_3$, bottom) spectra of **3g** recorded at 298 K.

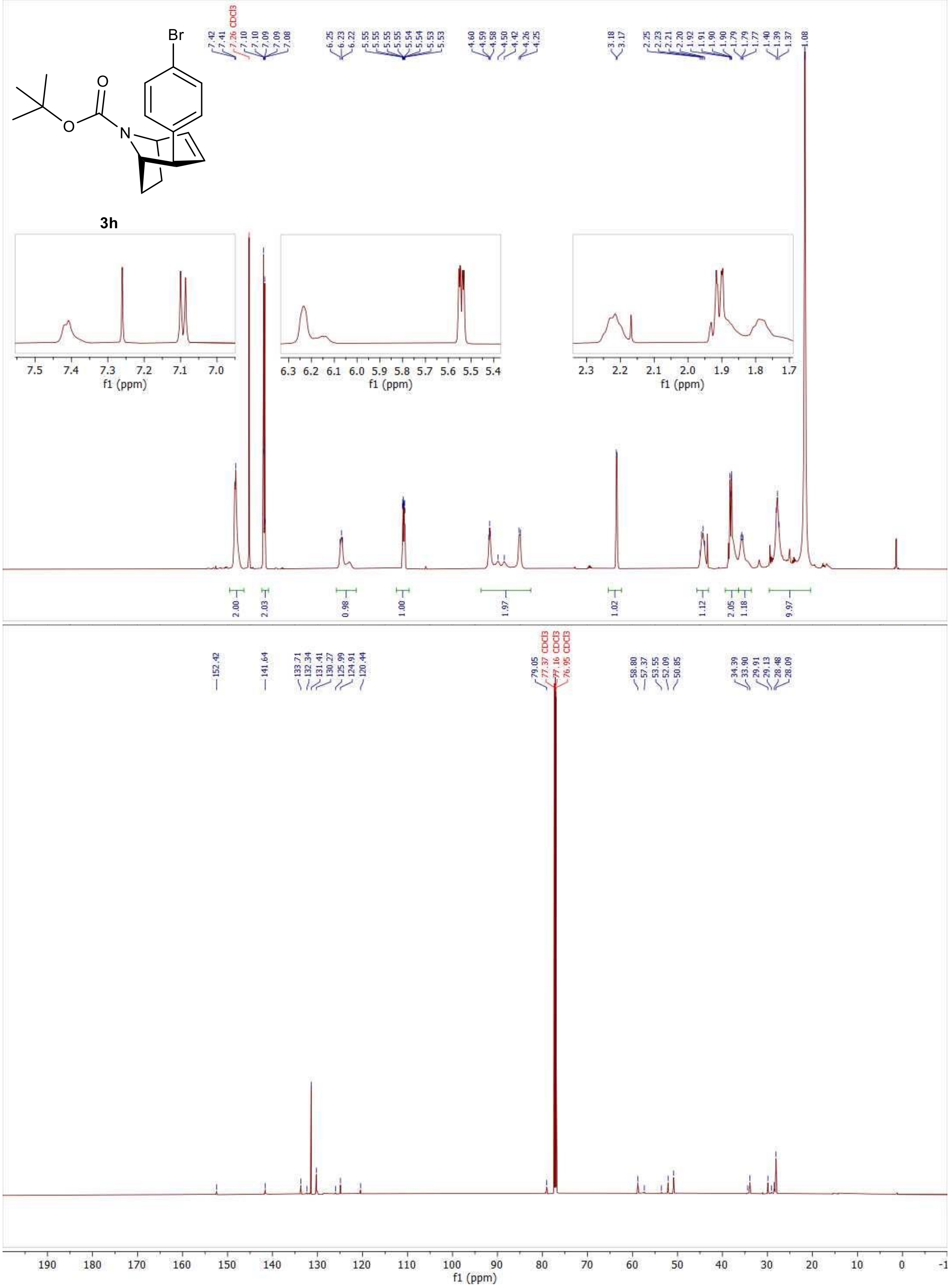

Figure S13. ^{1}H NMR (600 MHz, $CDCl_3$, top) and ^{13}C NMR (151 MHz, $CDCl_3$, bottom) spectra of **3h** recorded at 298 K.

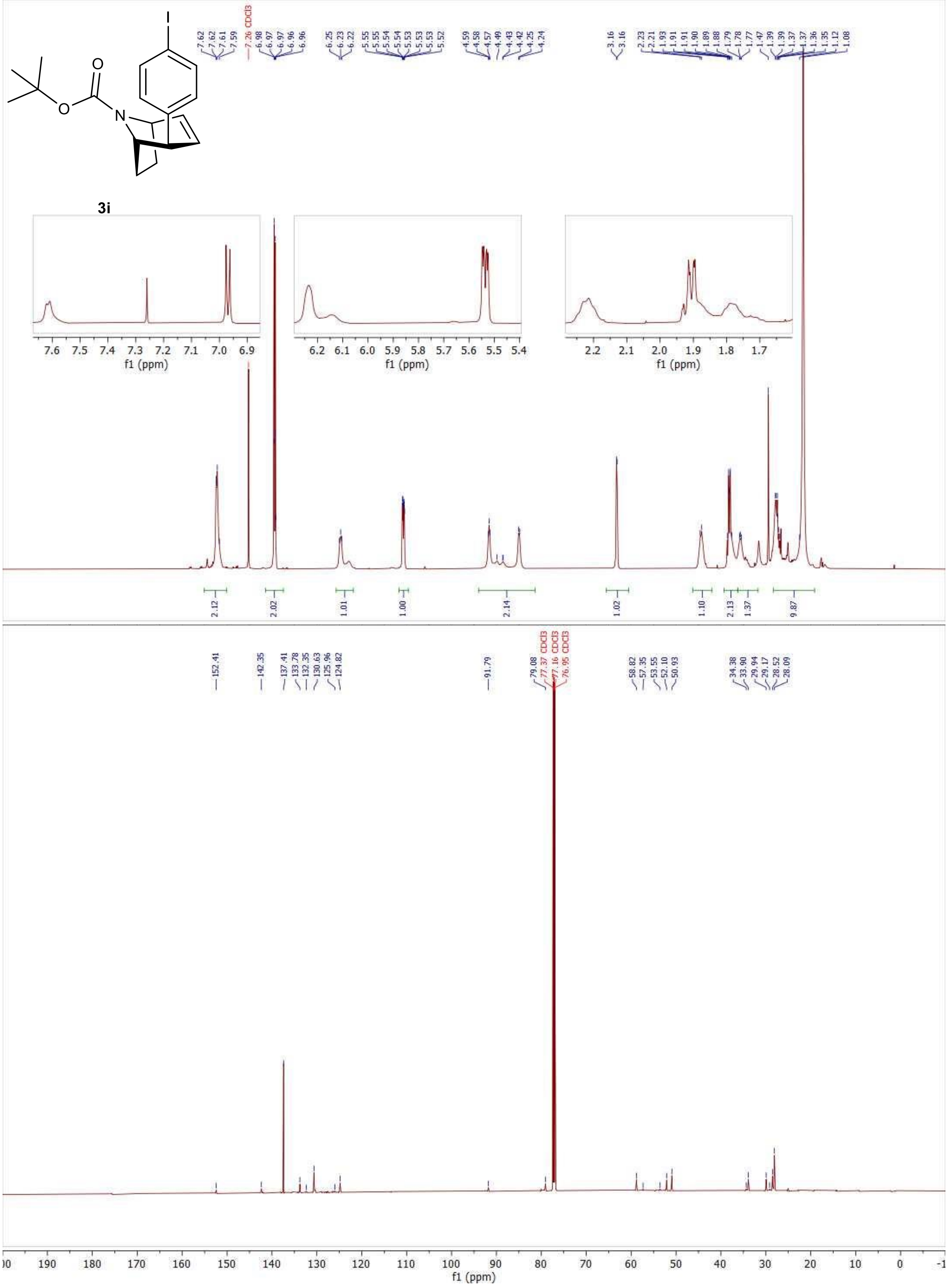

Figure S14. ^{1}H NMR (600 MHz, CDCl$_3$, top) and ^{13}C NMR (151 MHz, CDCl$_3$, bottom) spectra of **3i** recorded at 298 K.

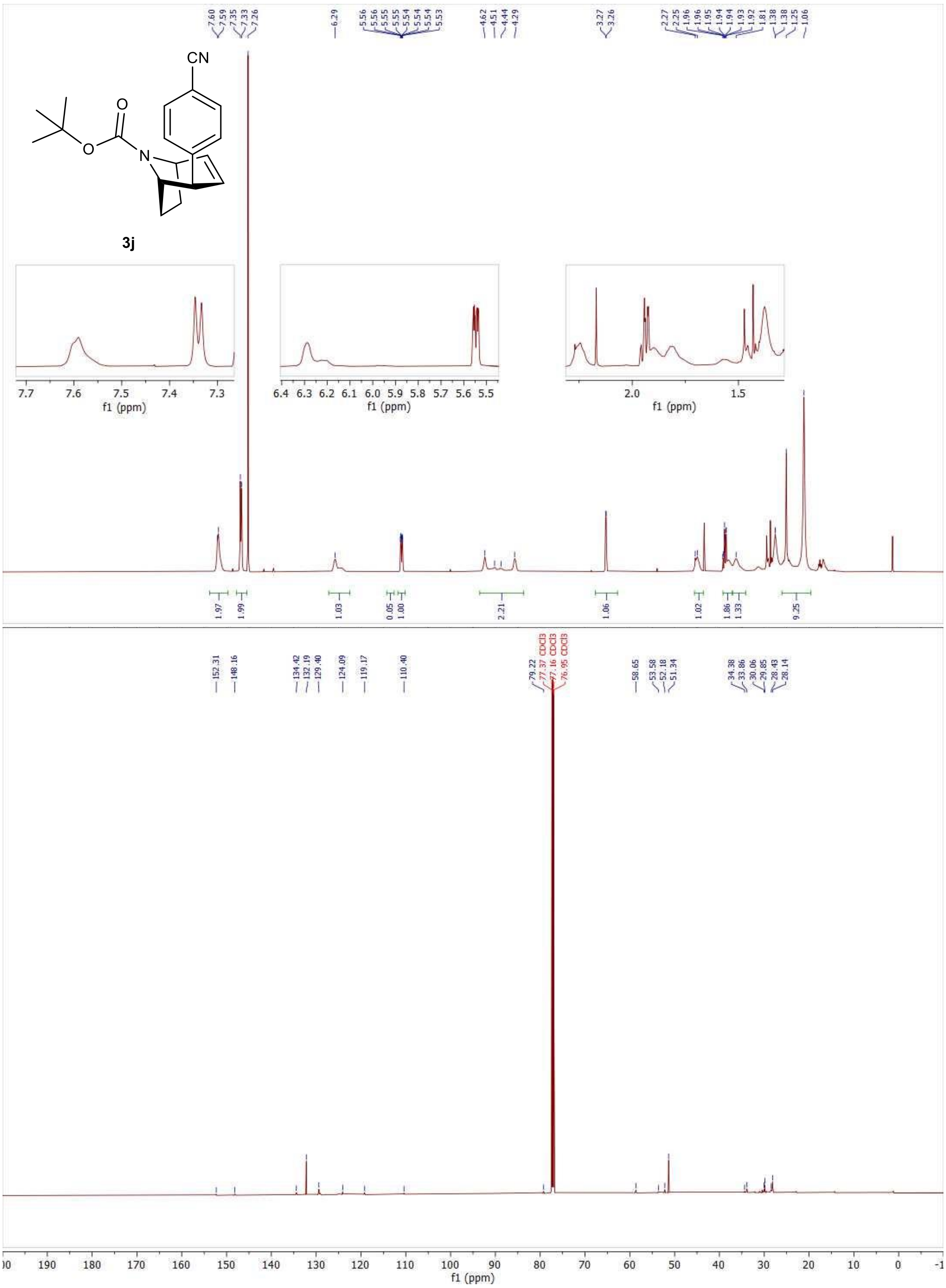

Figure S15. ^{1}H NMR (600 MHz, $CDCl_3$, top) and ^{13}C NMR (151 MHz, $CDCl_3$, bottom) spectra of **3j** recorded at 298 K.

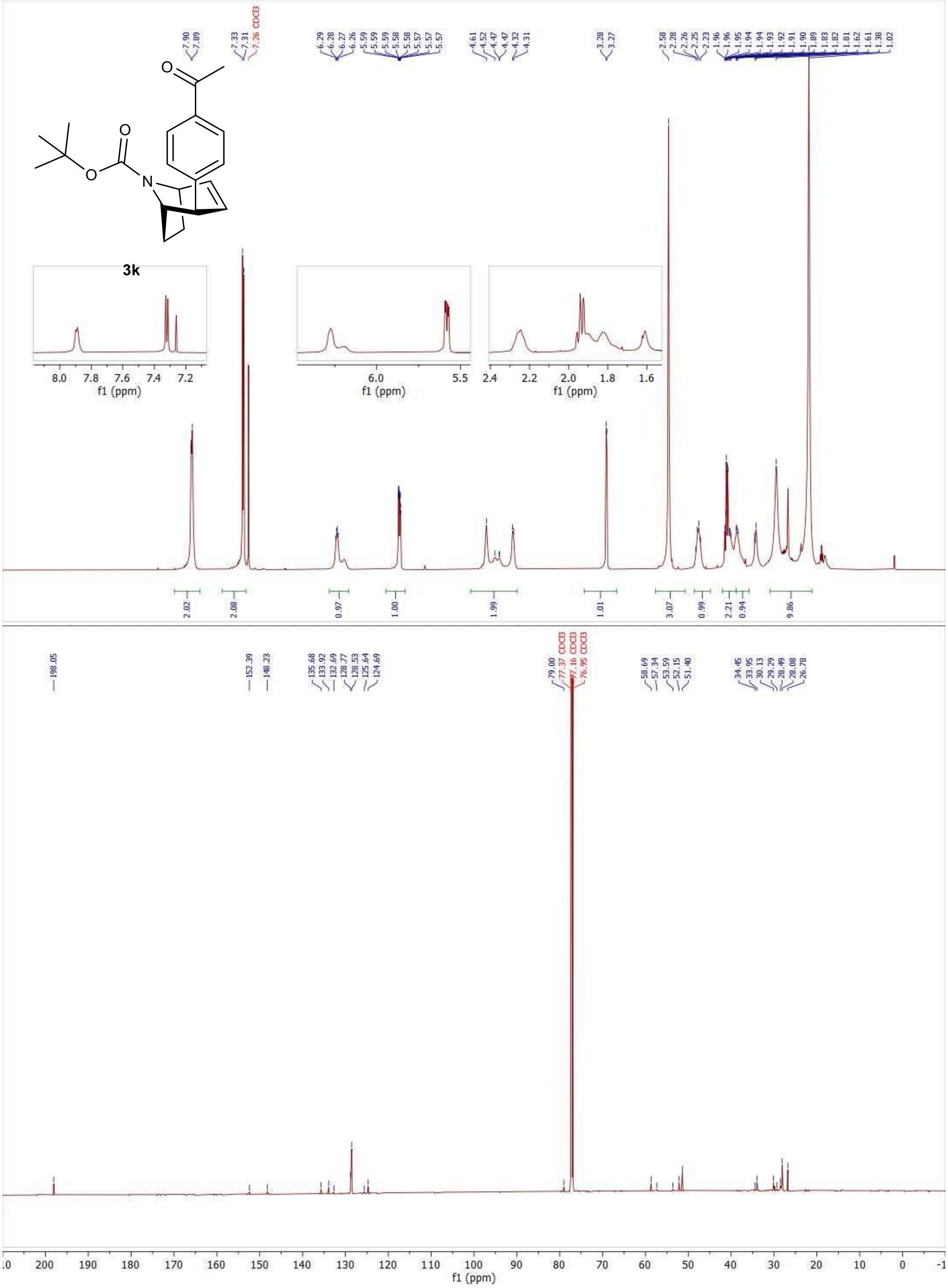

Figure S16. ^{1}H NMR (600 MHz, $CDCl_3$, top) and ^{13}C NMR (151 MHz, $CDCl_3$, bottom) spectra of **3k** recorded at 298 K.

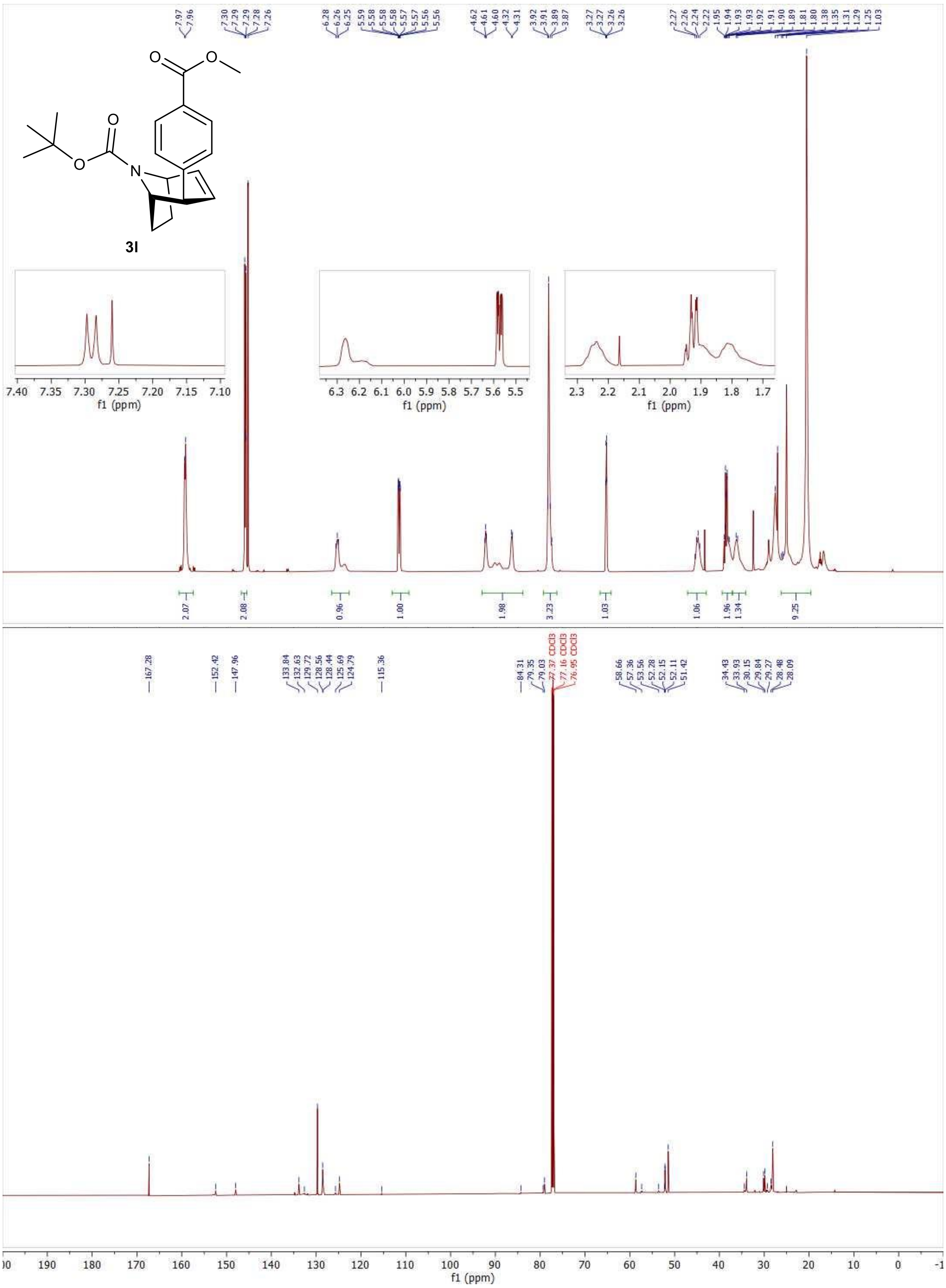

Figure S17. ^{1}H NMR (600 MHz, $CDCl_3$, top) and ^{13}C NMR (151 MHz, $CDCl_3$, bottom) spectra of **3l** recorded at 298 K.

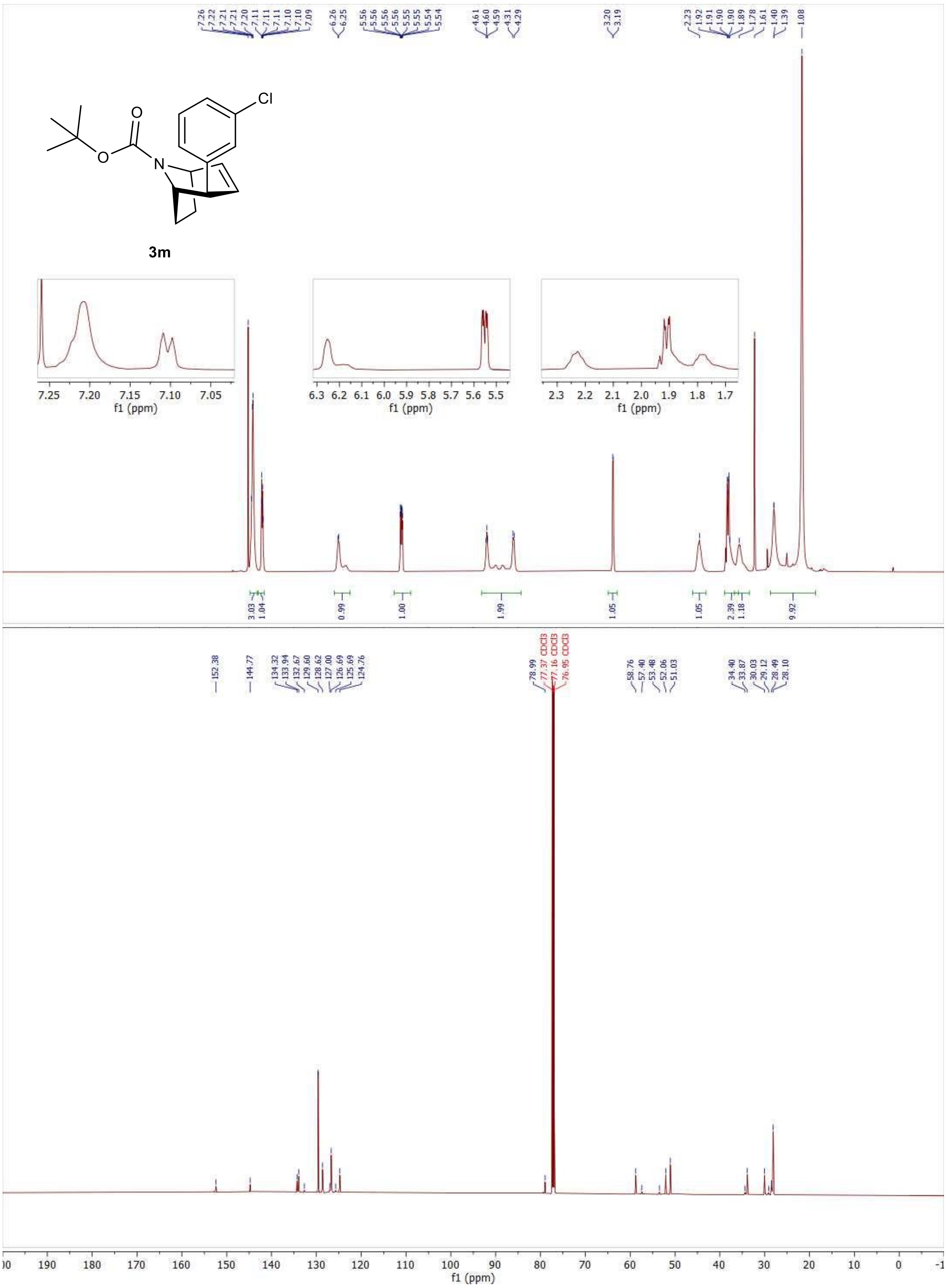

Figure S18. ^{1}H NMR (600 MHz, $CDCl_3$, top) and ^{13}C NMR (151 MHz, $CDCl_3$, bottom) spectra of **3m** recorded at 298 K.

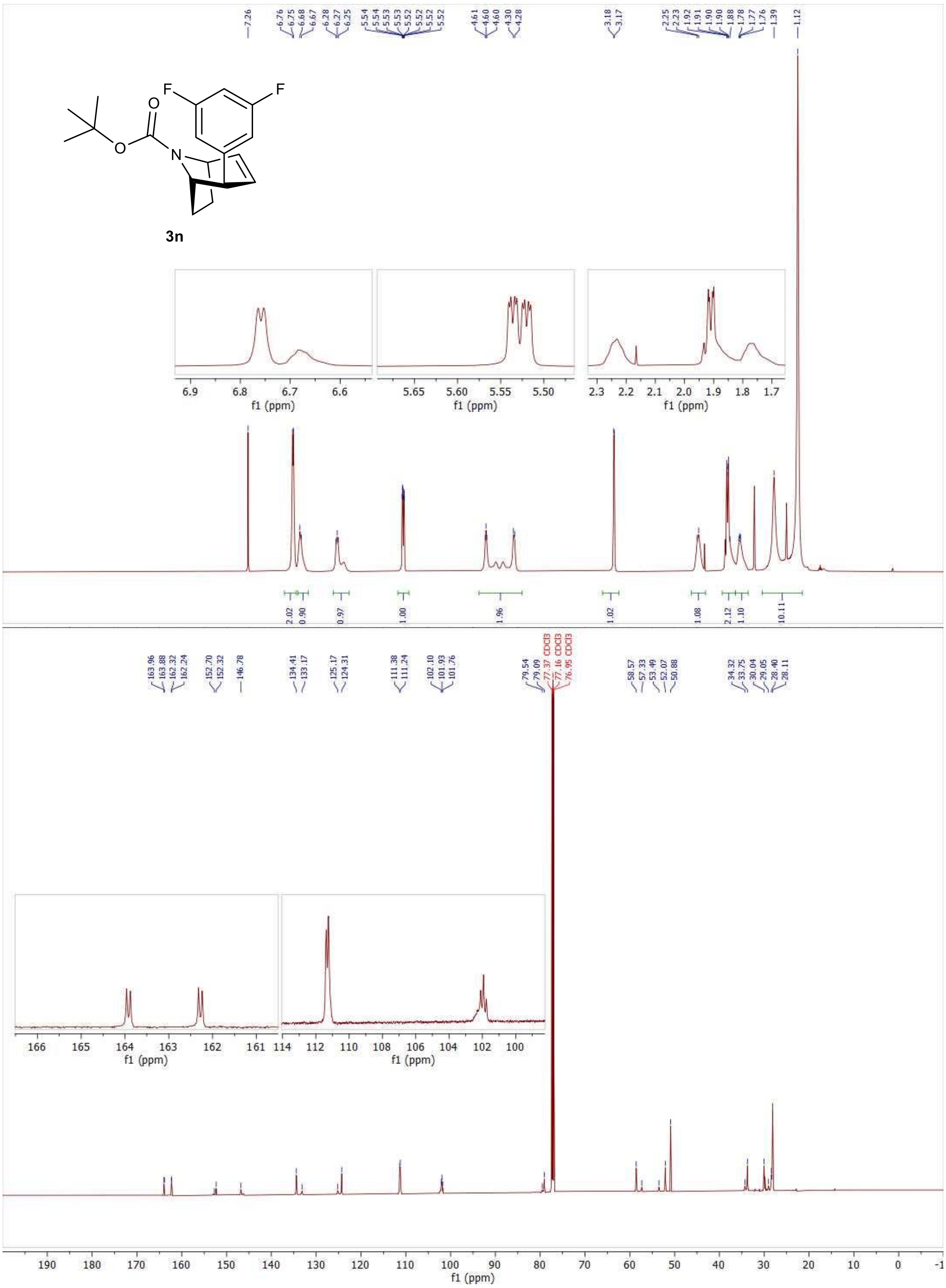

Figure S19. ^{1}H NMR (600 MHz, $CDCl_3$, top) and ^{13}C NMR (151 MHz, $CDCl_3$, bottom) spectra of **3n** recorded at 298 K.

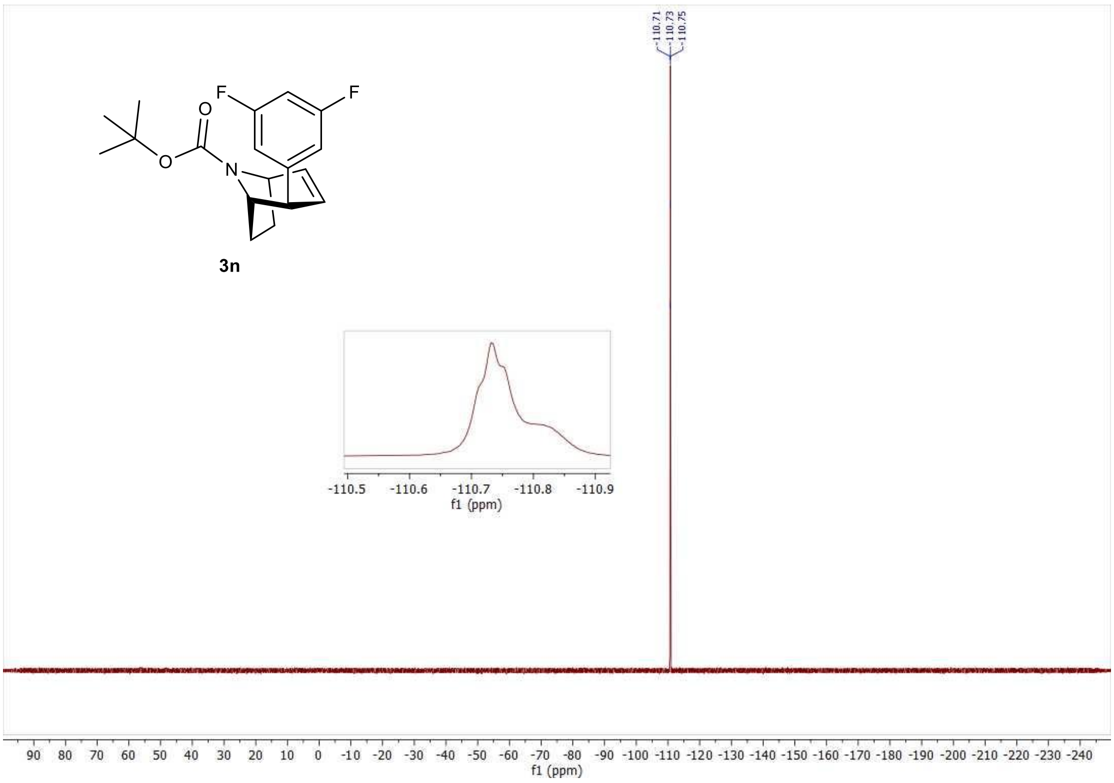

Figure S19-1. ^{19}F NMR (377 MHz, $CDCl_3$) spectra of **3n** recorded at 298 K.

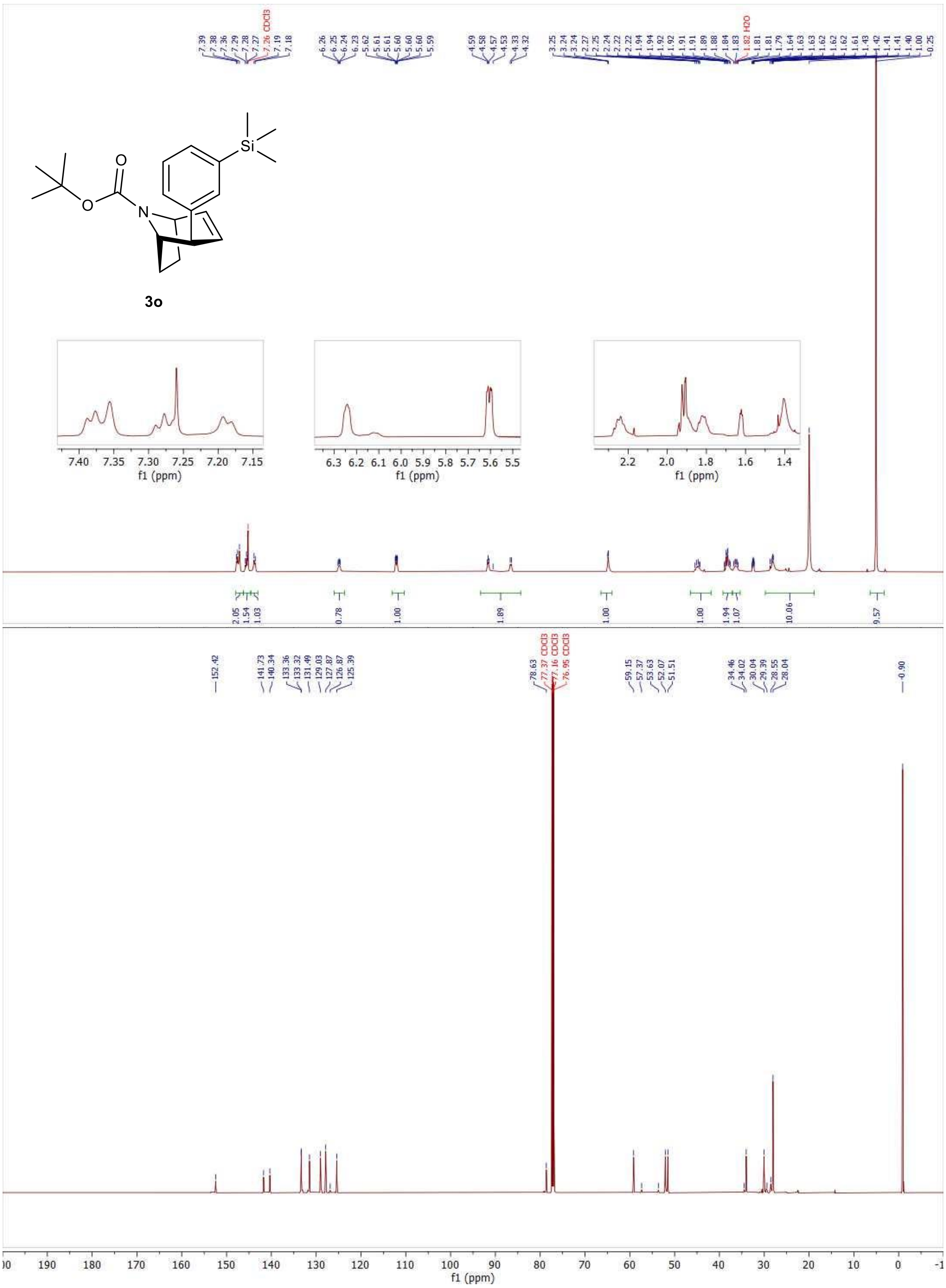

Figure S20. ^{1}H NMR (600 MHz, $CDCl_3$, top) and ^{13}C NMR (151 MHz, $CDCl_3$, bottom) spectra of **3o** recorded at 298 K.

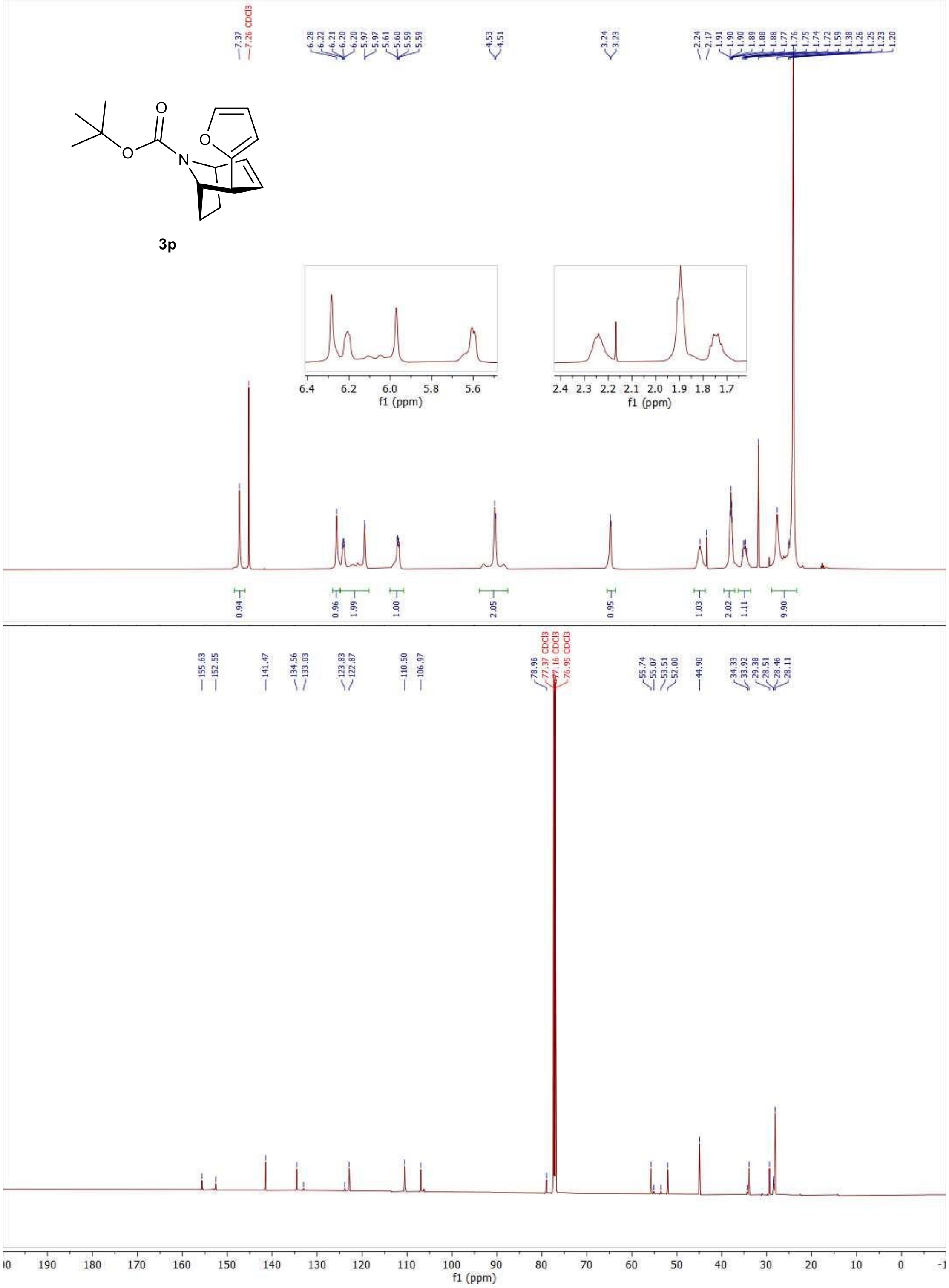

Figure S21. ^{1}H NMR (600 MHz, $CDCl_3$, top) and ^{13}C NMR (151 MHz, $CDCl_3$, bottom) spectra of **3p** recorded at 298 K.

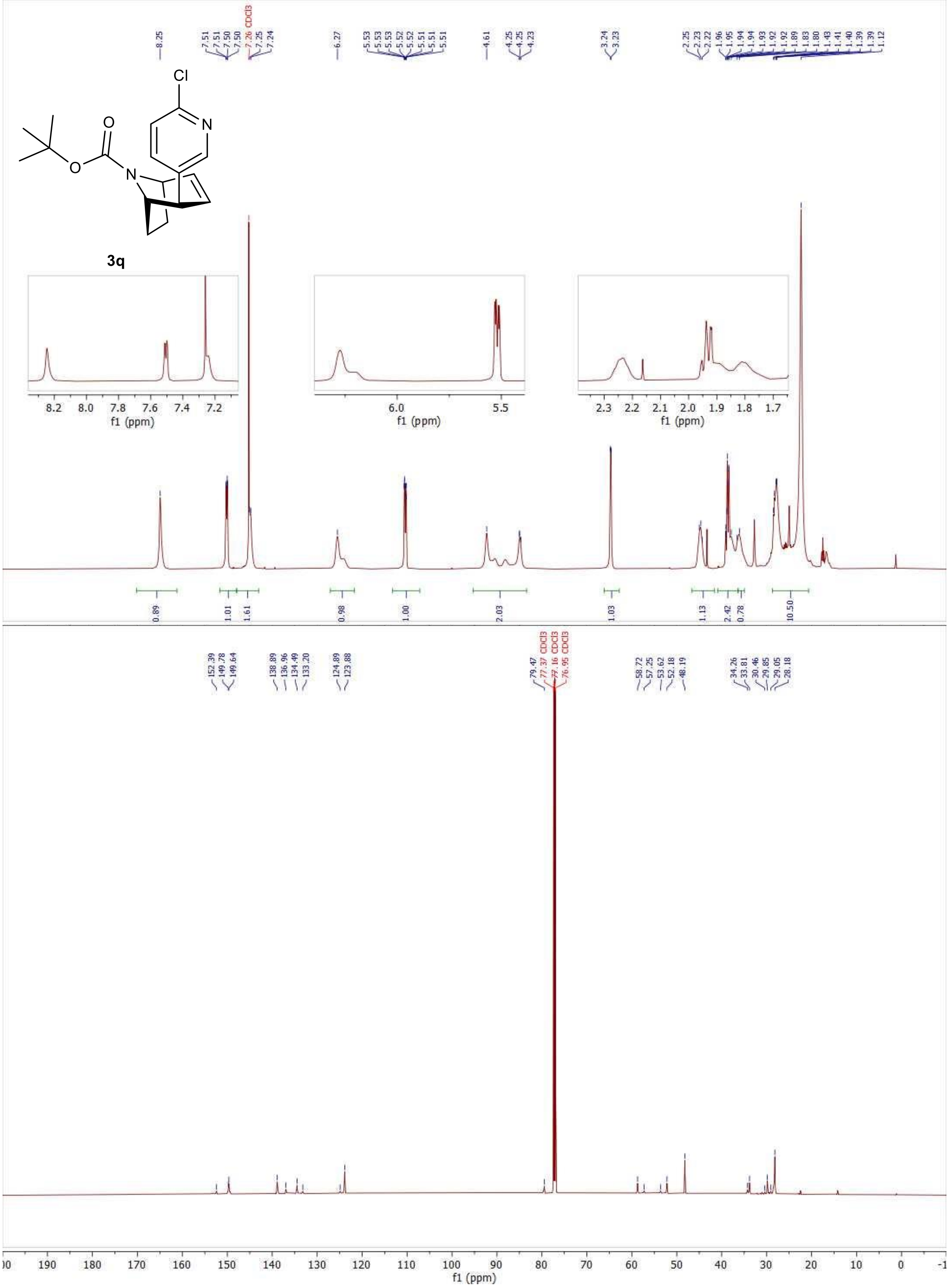

Figure S22. ^{1}H NMR (600 MHz, $CDCl_3$, top) and ^{13}C NMR (151 MHz, $CDCl_3$, bottom) spectra of **3q** recorded at 298 K.

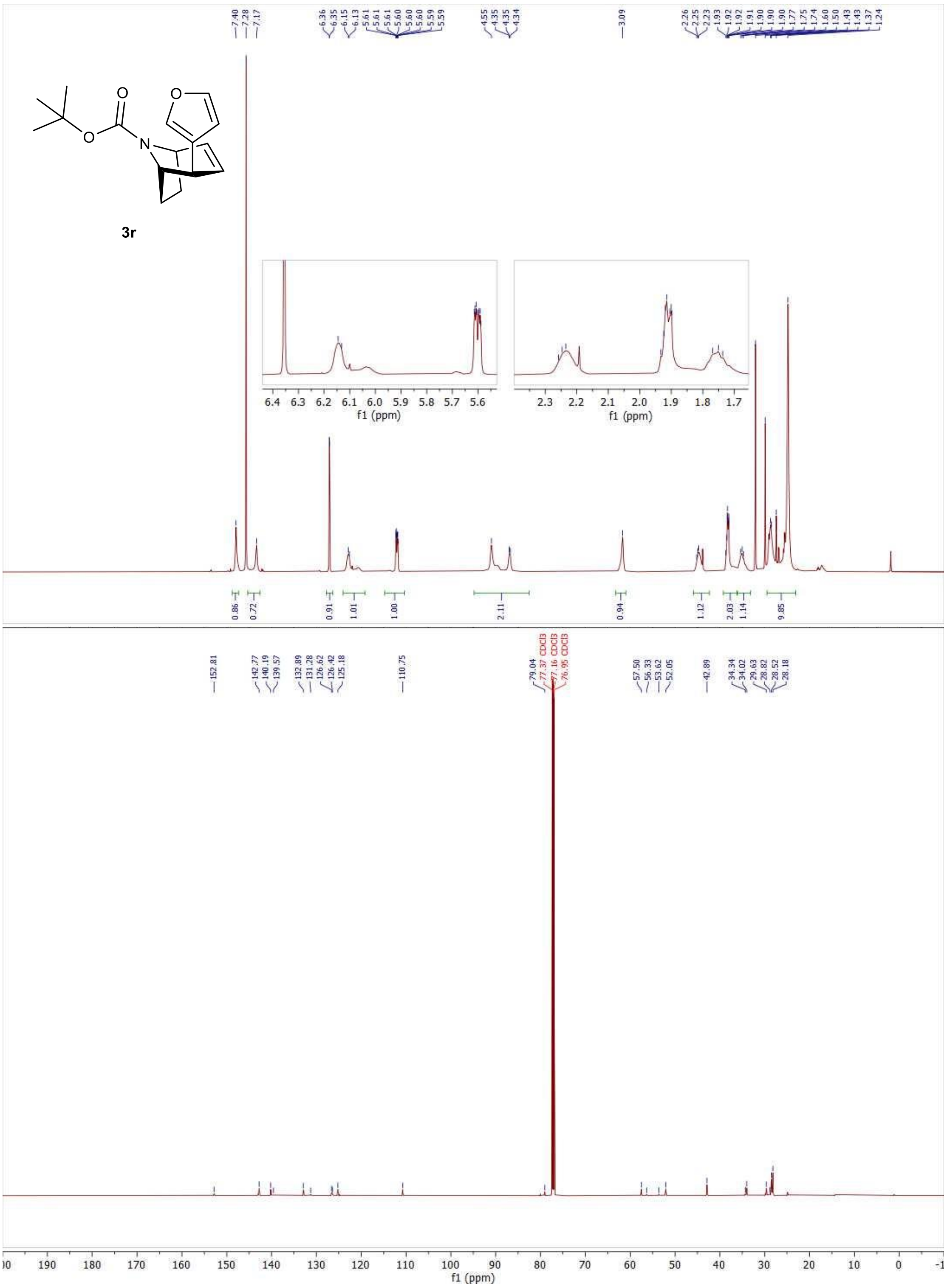

Figure S23. ^{1}H NMR (600 MHz, $CDCl_3$, top) and ^{13}C NMR (151 MHz, $CDCl_3$, bottom) spectra of **3r** recorded at 298 K.

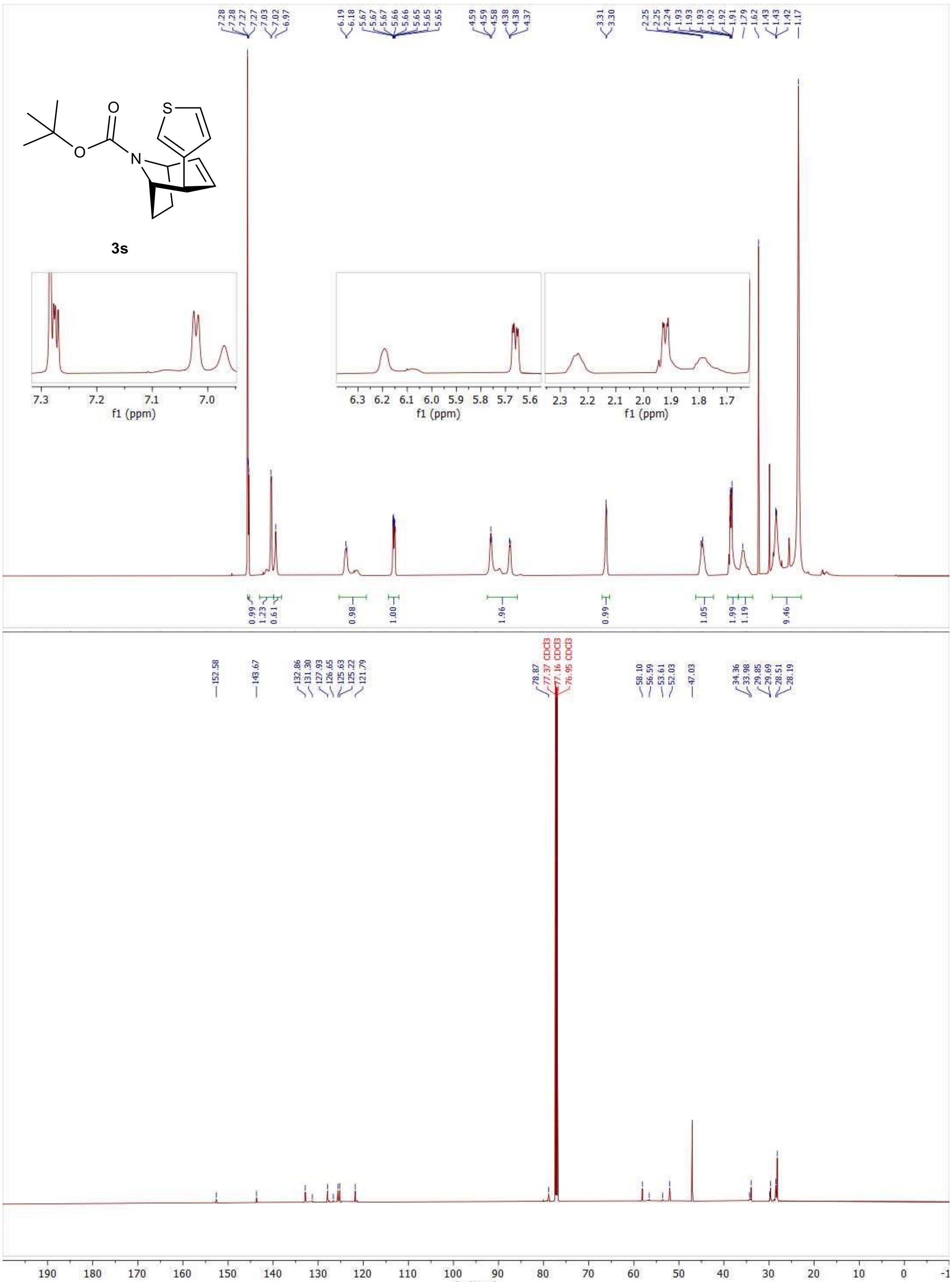

Figure S24. ^{1}H NMR (600 MHz, $CDCl_3$, top) and ^{13}C NMR (151 MHz, $CDCl_3$, bottom) spectra of **3s** recorded at 298 K.

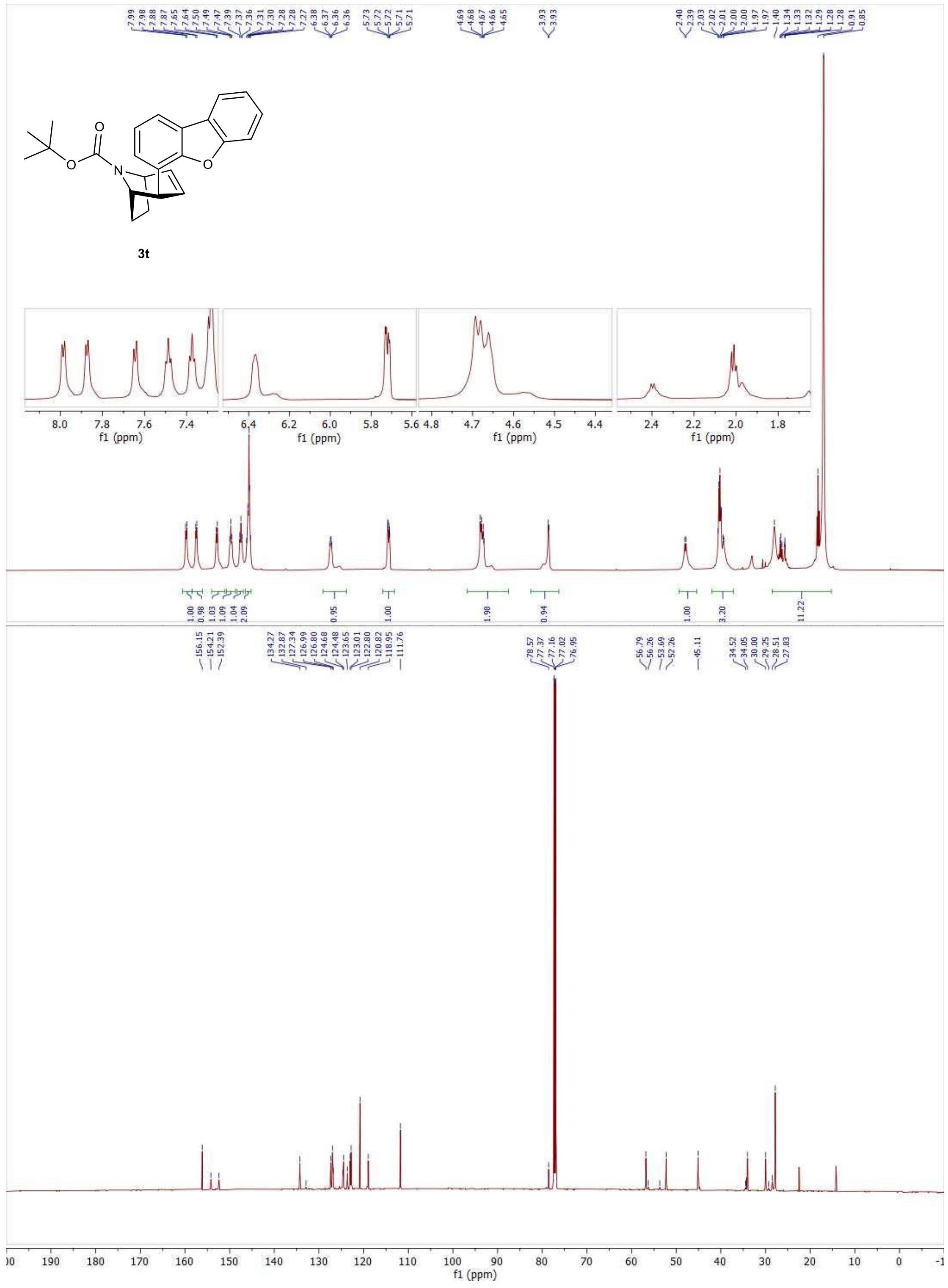

Figure S25. ^{1}H NMR (600 MHz, $CDCl_3$, top) and ^{13}C NMR (151 MHz, $CDCl_3$, bottom) spectra of **3t** recorded at 298 K.

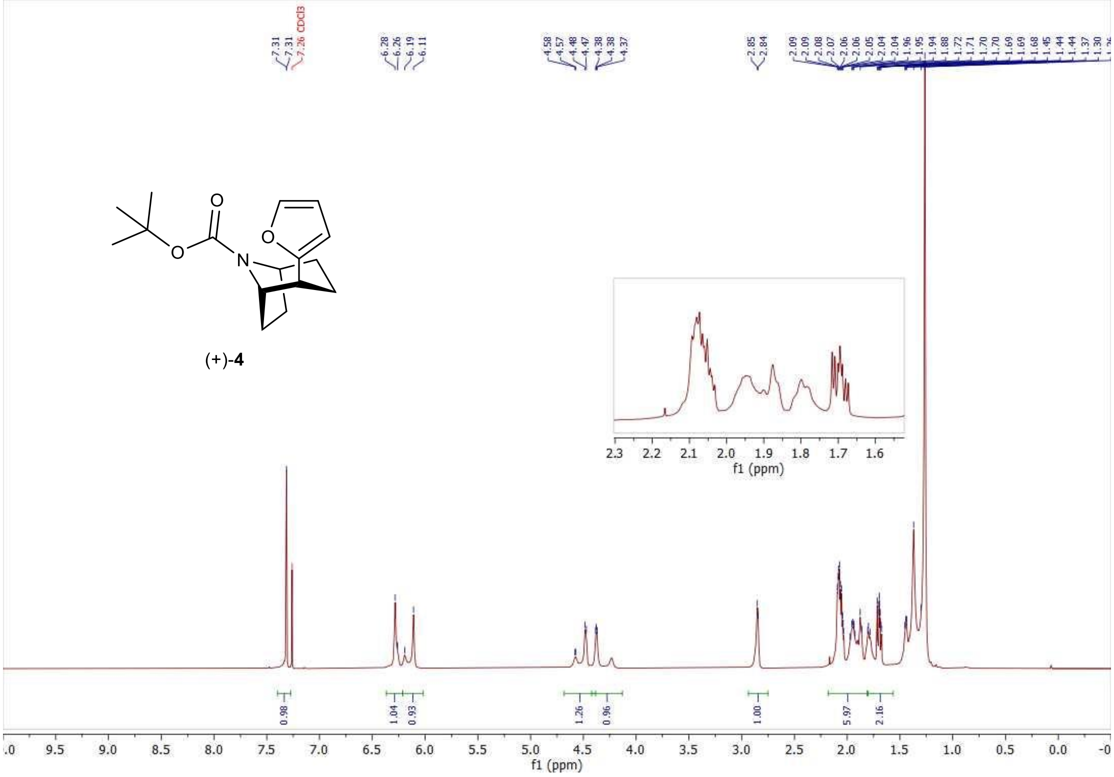

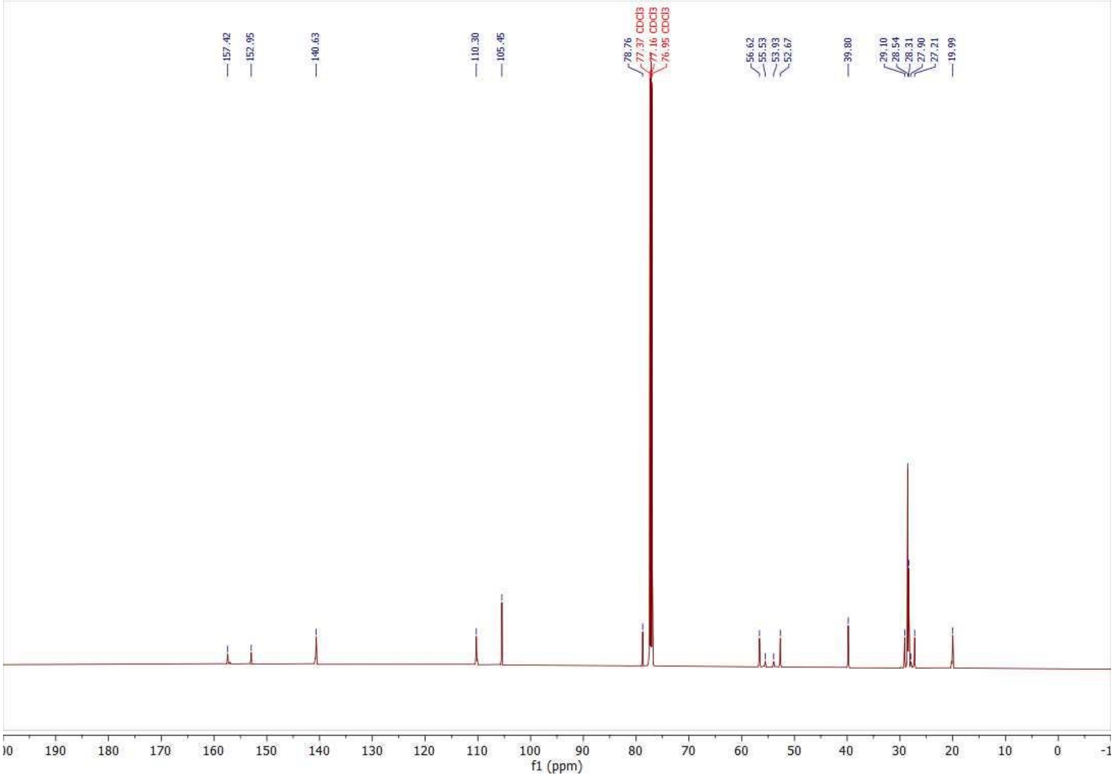

Figure S26. ^{1}H NMR (600 MHz, $CDCl_3$, top) and ^{13}C NMR (151 MHz, $CDCl_3$, bottom) spectra of (+)-**4** recorded at 298 K.

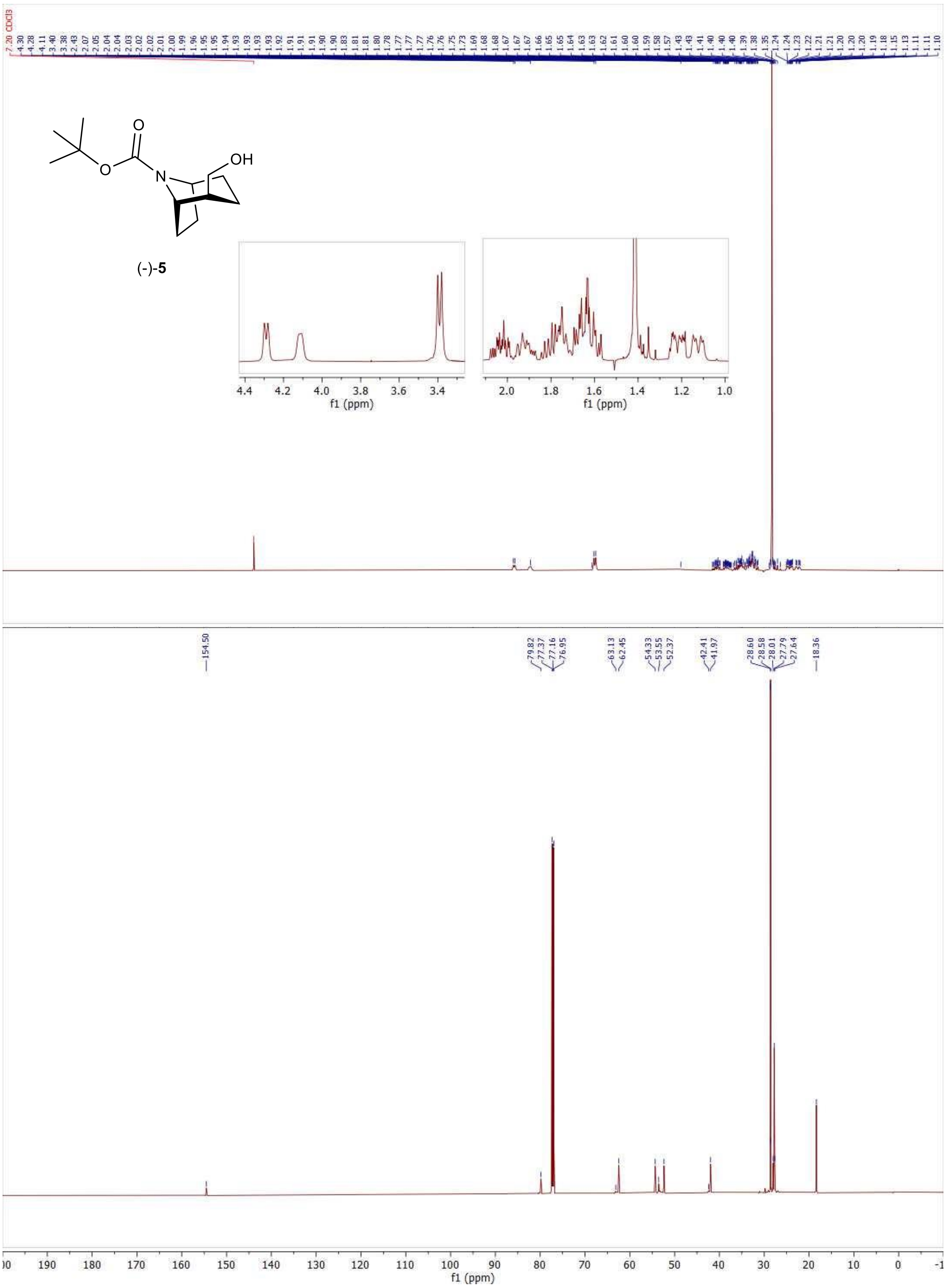

Figure S27. ^{1}H NMR (600 MHz, $CDCl_3$, top) and ^{13}C NMR (151 MHz, $CDCl_3$, bottom) spectra of **(−)-5** recorded at 298 K.

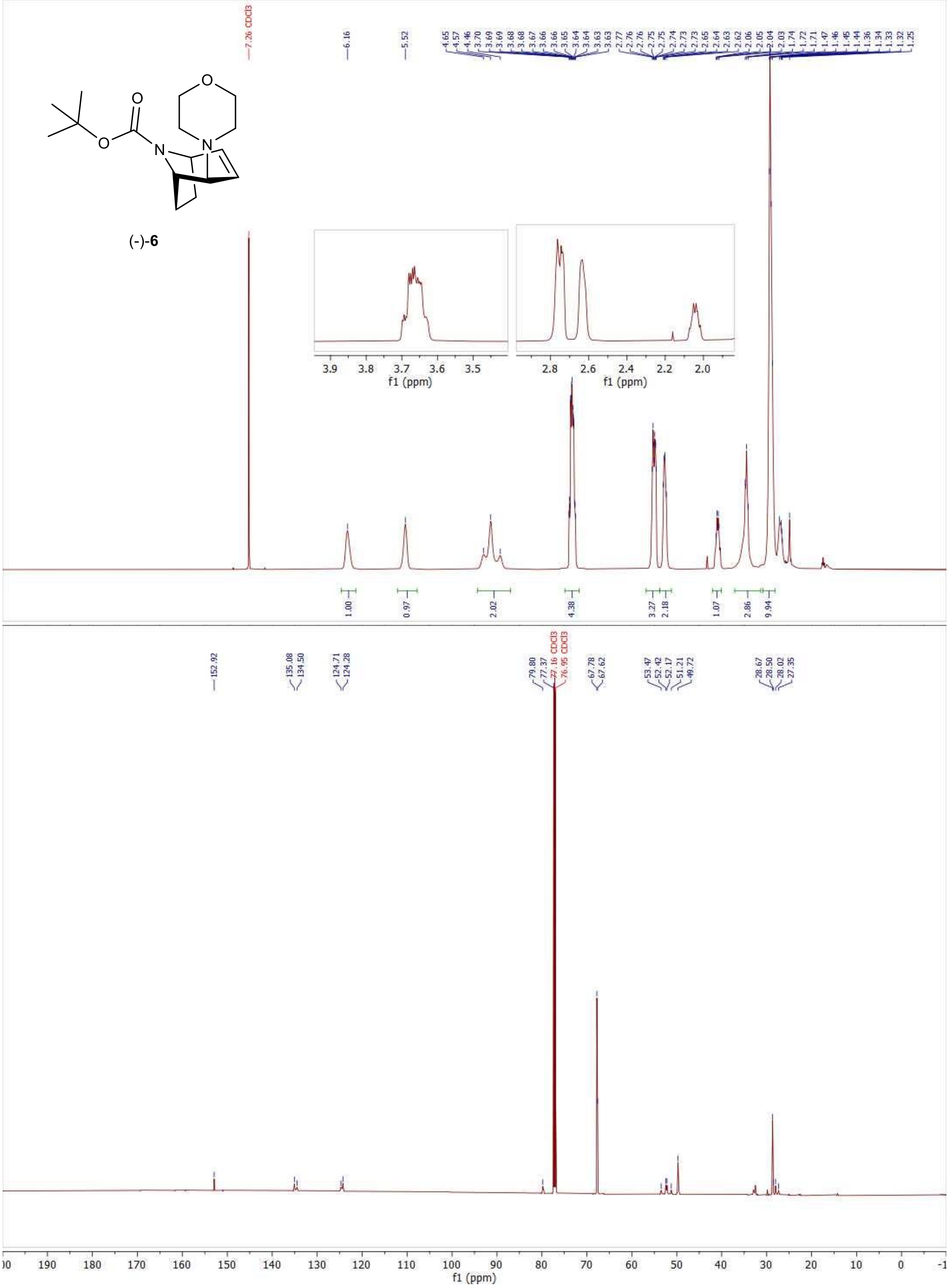

Figure S28. ^{1}H NMR (600 MHz, $CDCl_3$, top) and ^{13}C NMR (151 MHz, $CDCl_3$, bottom) spectra of **(−)-6** recorded at 298 K.

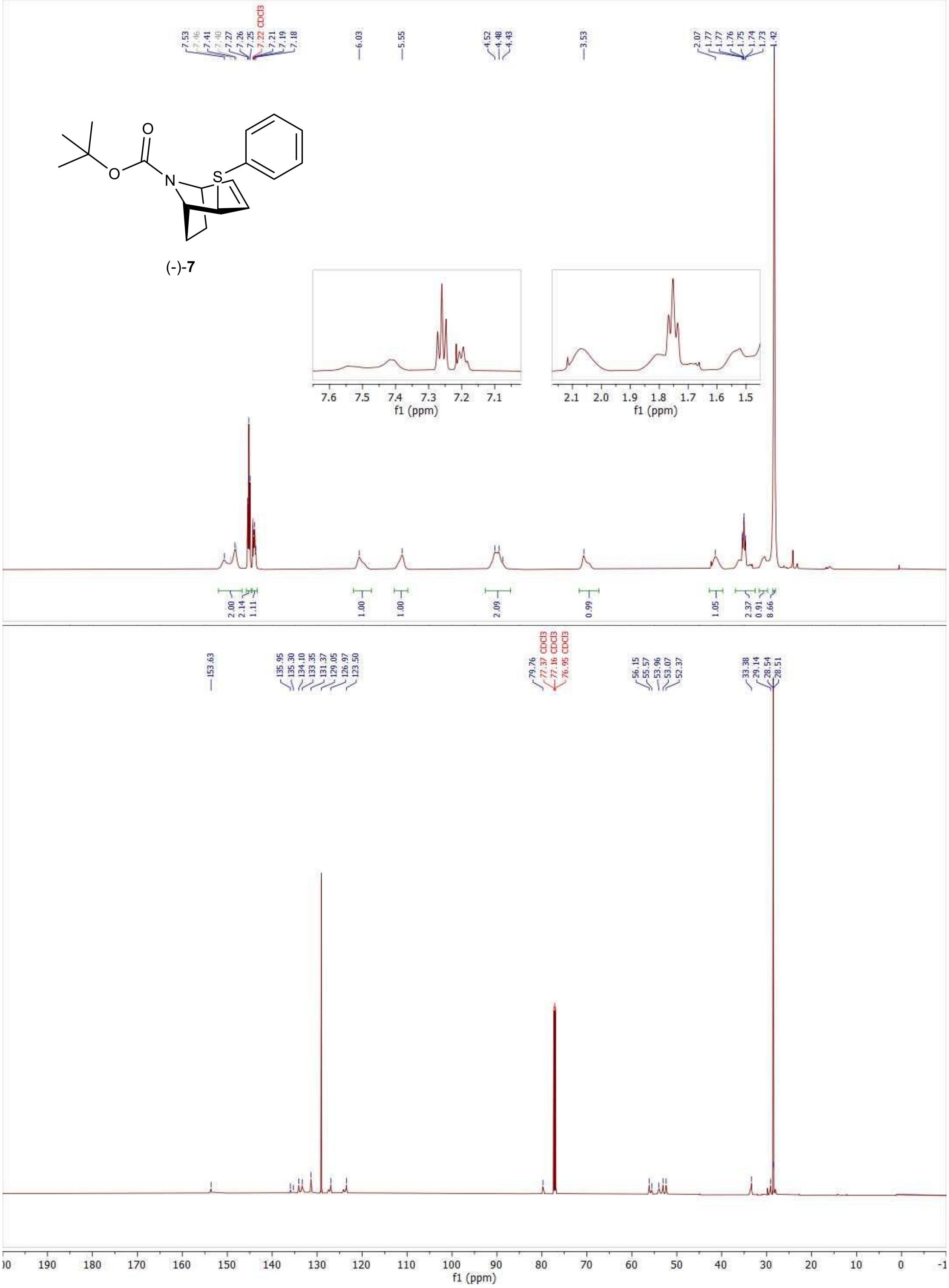

Figure S29. ^{1}H NMR (600 MHz, $CDCl_3$, top) and ^{13}C NMR (151 MHz, $CDCl_3$, bottom) spectra of **(−)-7** recorded at 298 K.

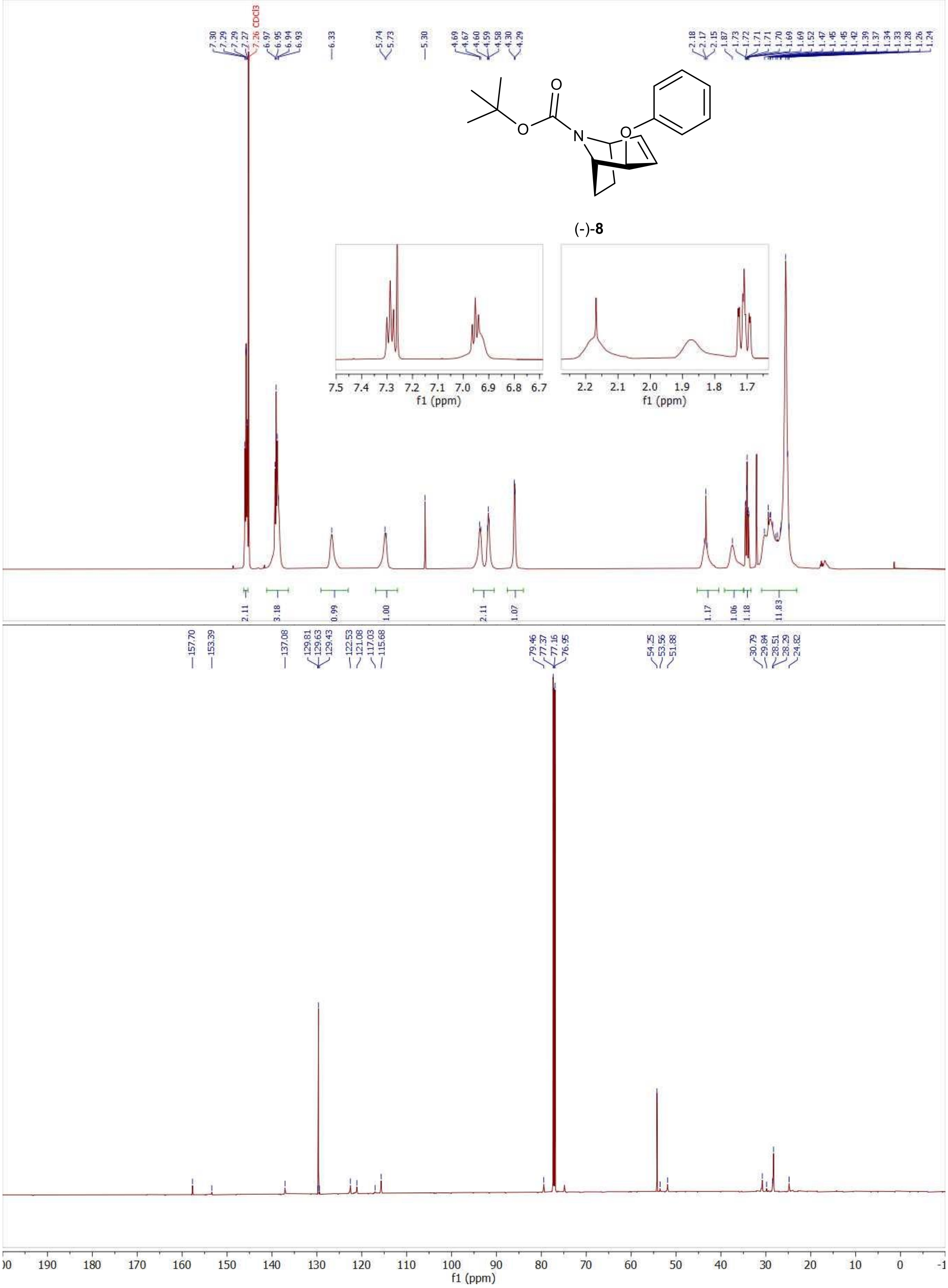

Figure S30. ^{1}H NMR (600 MHz, $CDCl_3$, top) and ^{13}C NMR (151 MHz, $CDCl_3$, bottom) spectra of **(−)-8** recorded at 298 K.

4. SFC Traces

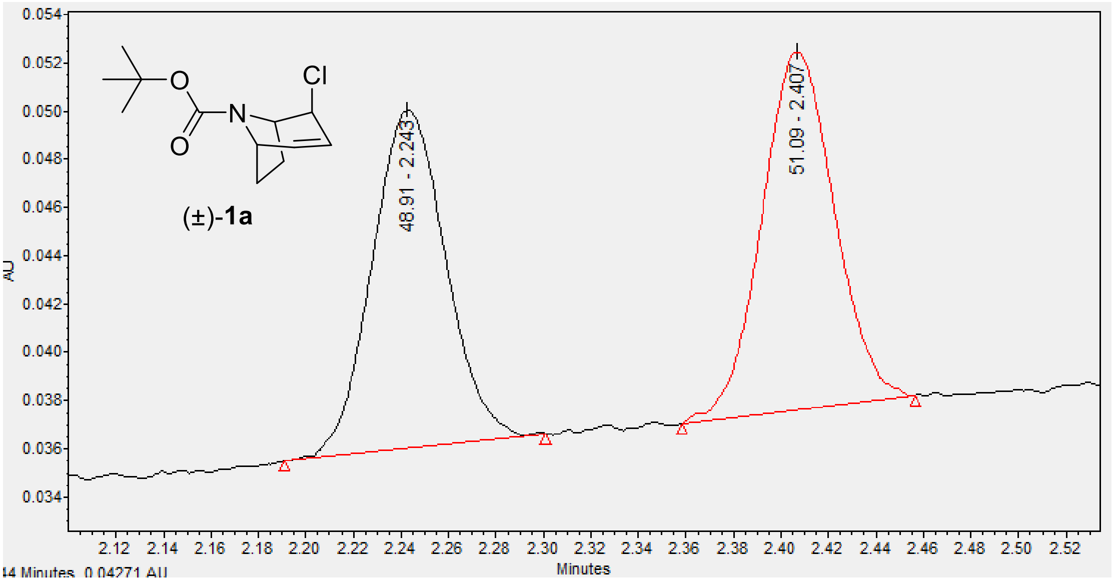

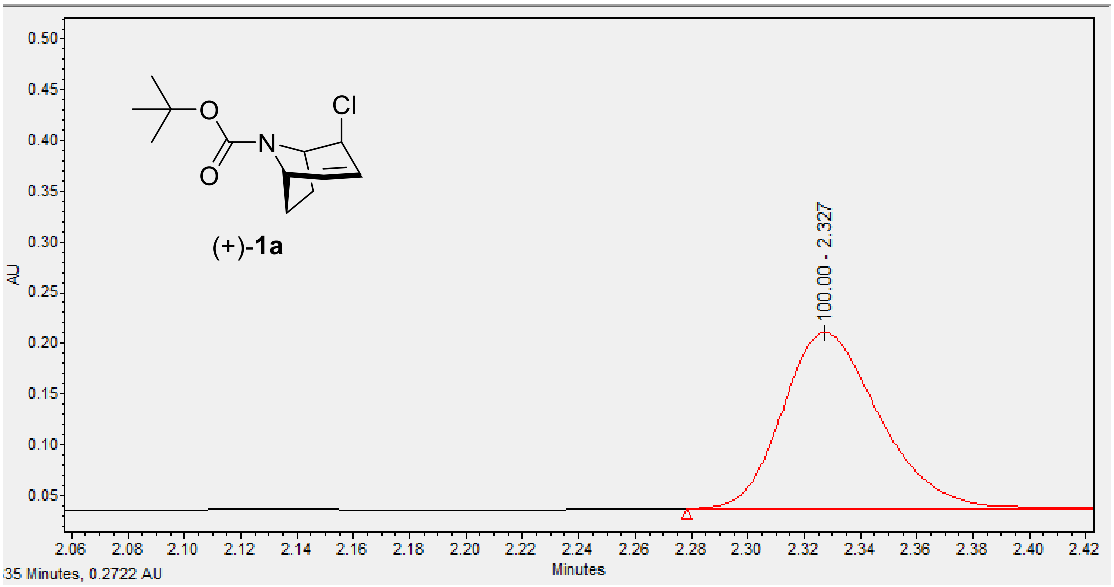

Figure S31. SFC trace of racemic (±)-**1a** and enantioenriched (+)-**1a**.

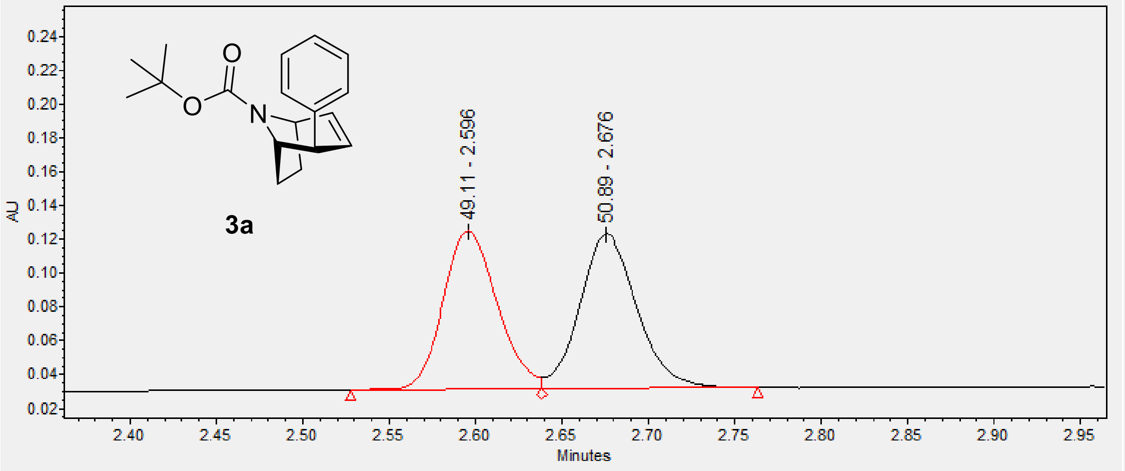

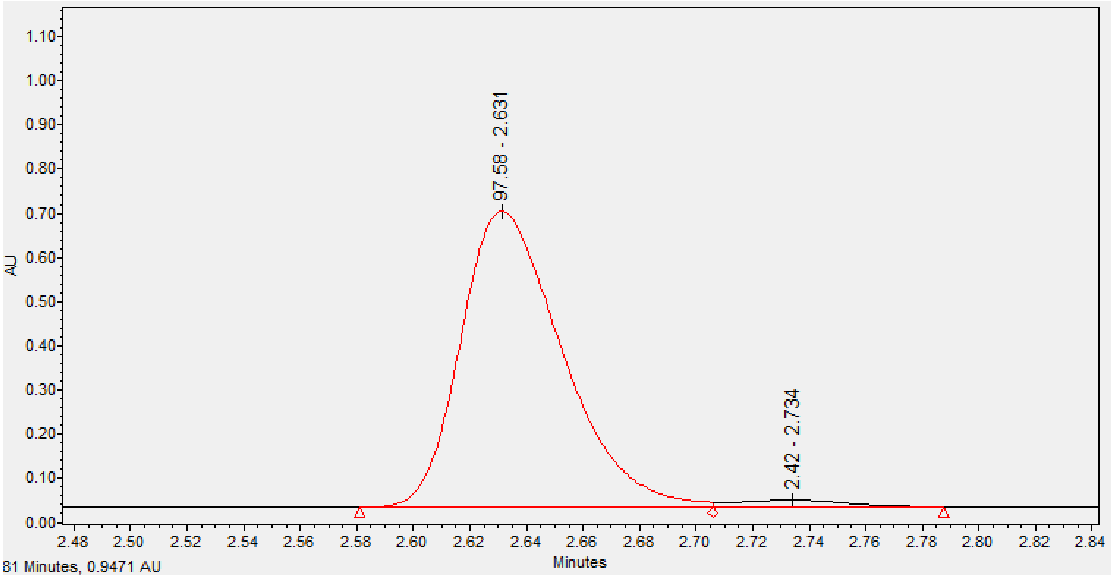

Figure S32. SFC trace of racemic (±)-**3a** and enantioenriched (–)-**3a**.

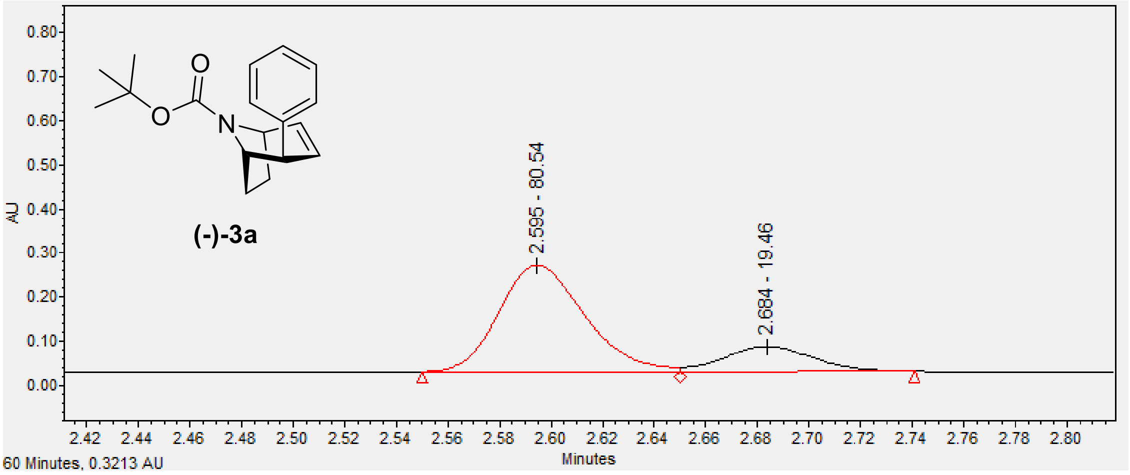

Figure S32-1. SFC trace of enantioenriched (-)-**3a** formed from reaction in Scheme **2a**.

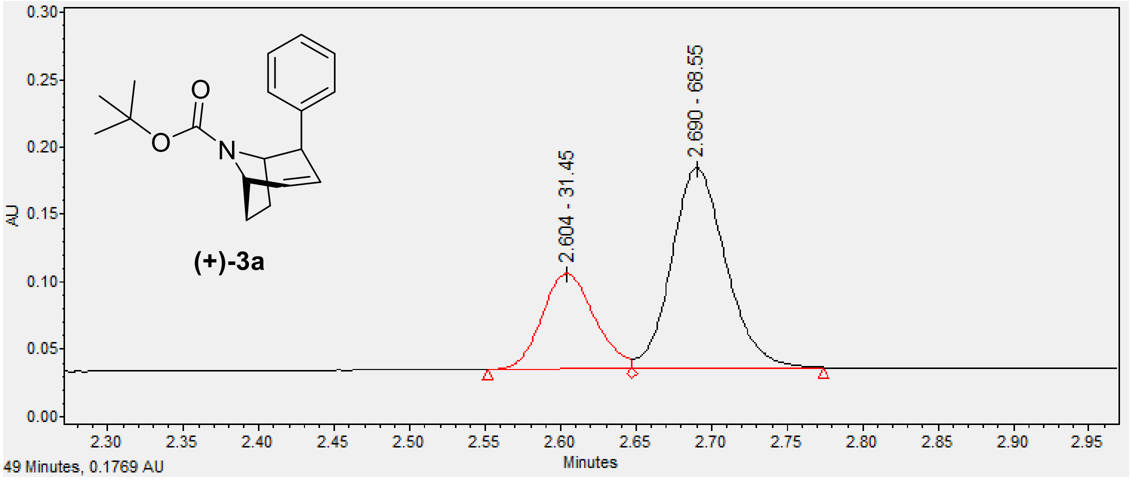

Figure S32-2. SFC trace of enantioenriched (+)-**3a** formed from reaction in Scheme **2b**.

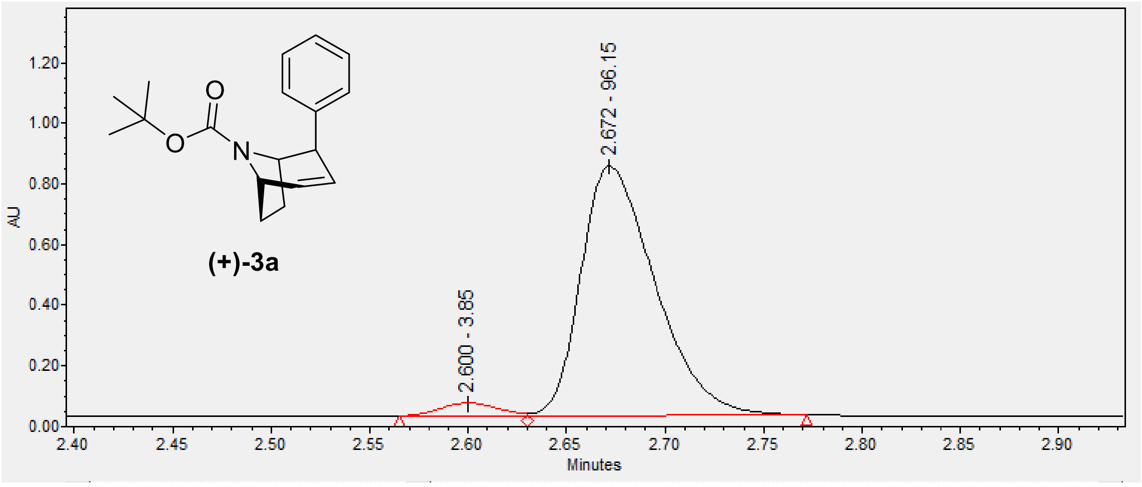

Figure S32-3. SFC trace of enantioenriched (+)-**3a** formed from reaction in Scheme **2c**.

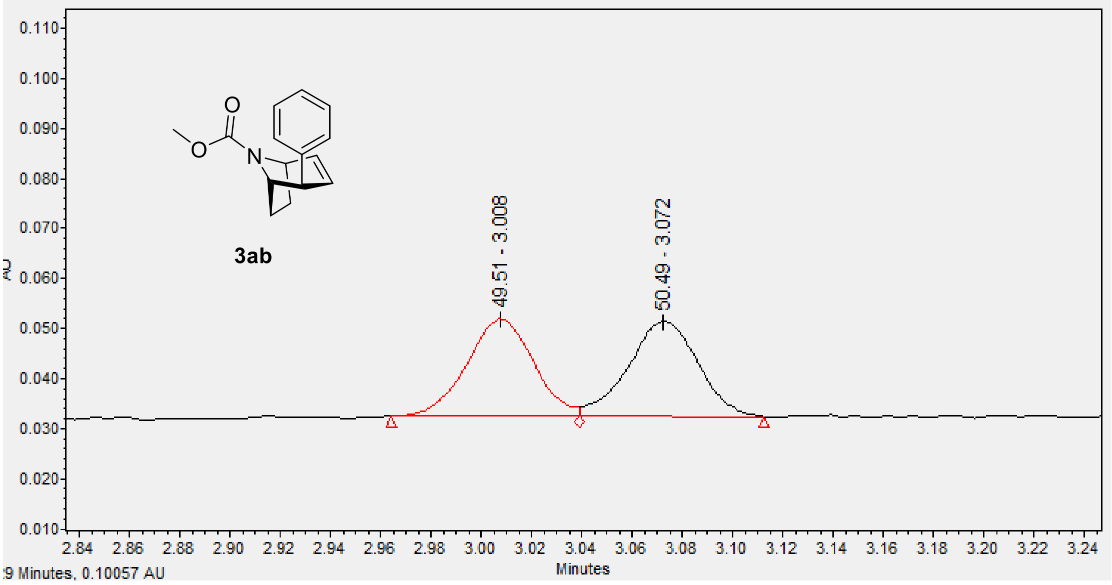

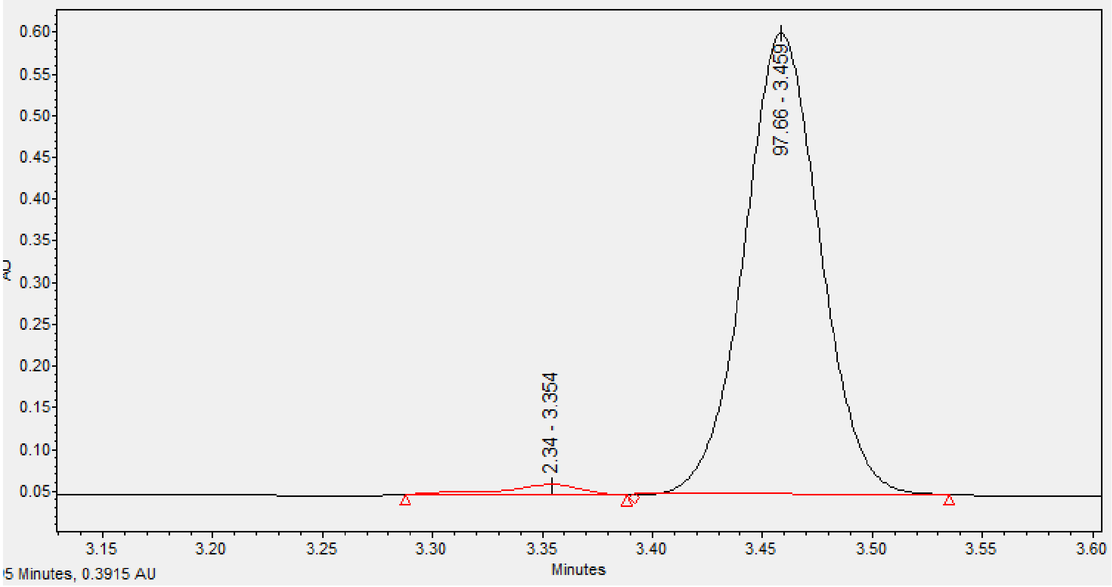

Figure S33. SFC trace of racemic (±)-**3ab** and enantioenriched (–)-**3ab**.

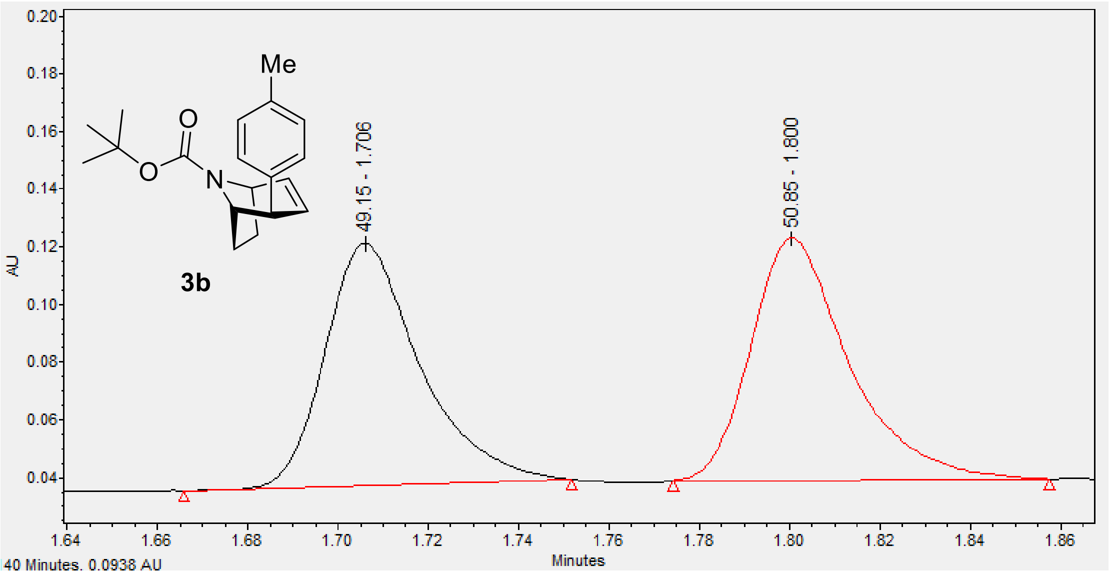

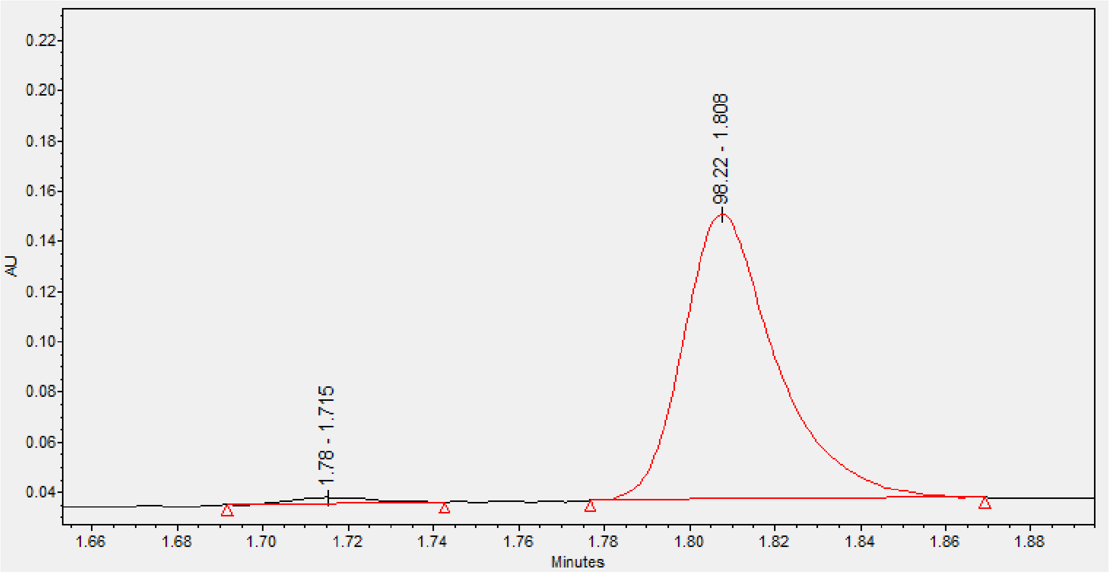

Figure S34. SFC trace of racemic (±)-**3b** and enantioenriched (–)-**3b**.

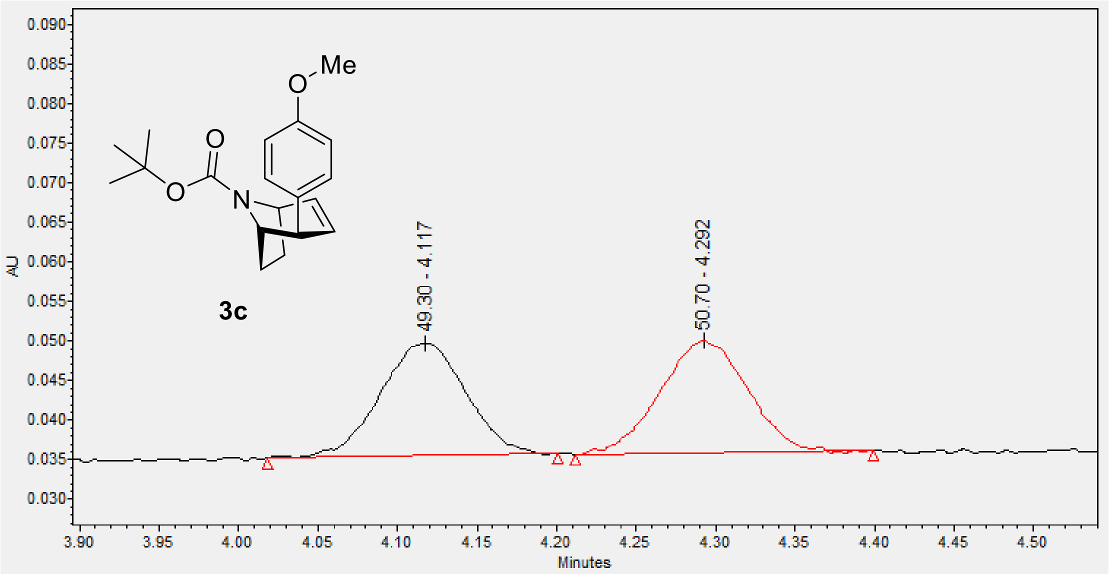

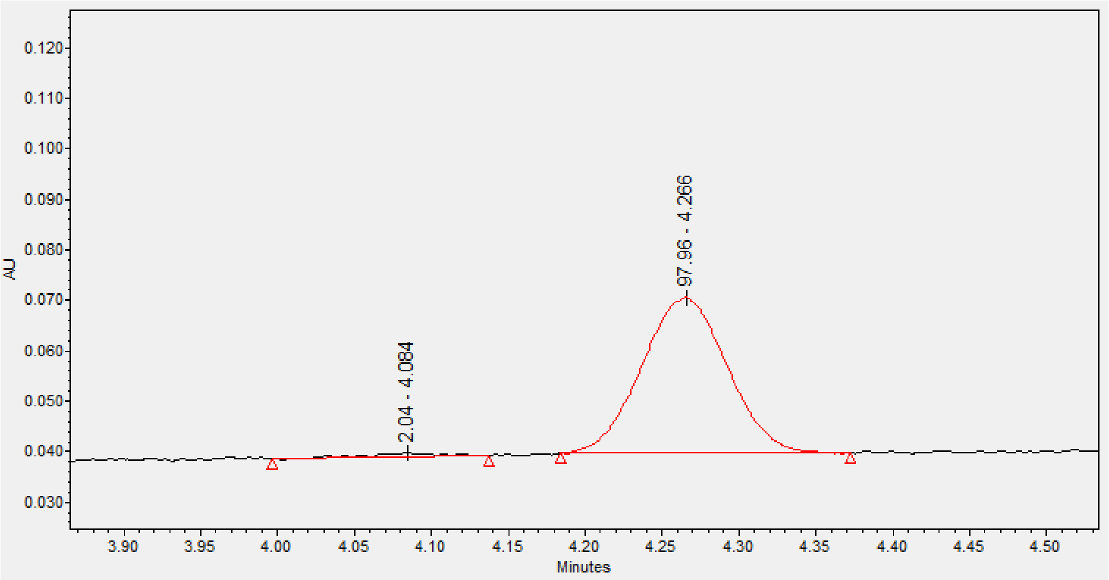

Figure S35. SFC trace of racemic (±)-**3c** and enantioenriched (–)-**3c**.

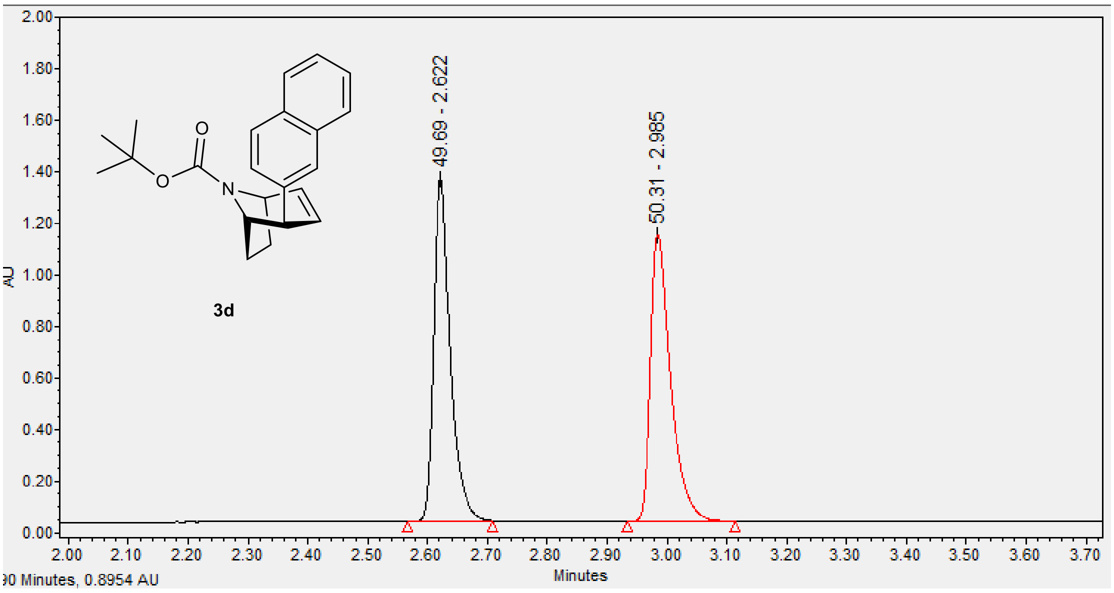

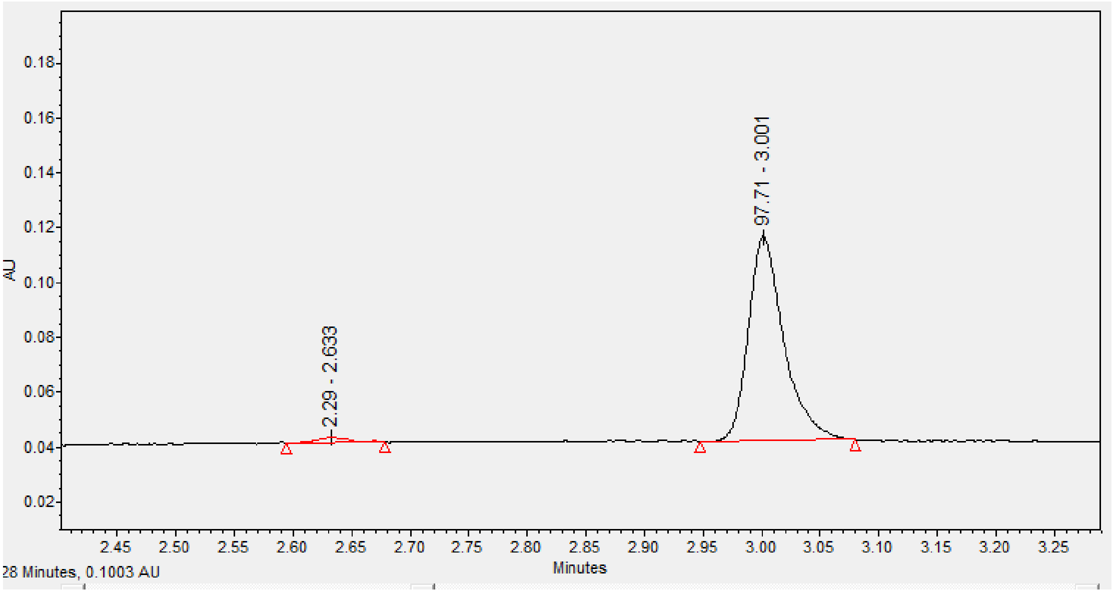

Figure S36. SFC trace of racemic (±)-**3d** and enantioenriched (–)-**3d**.

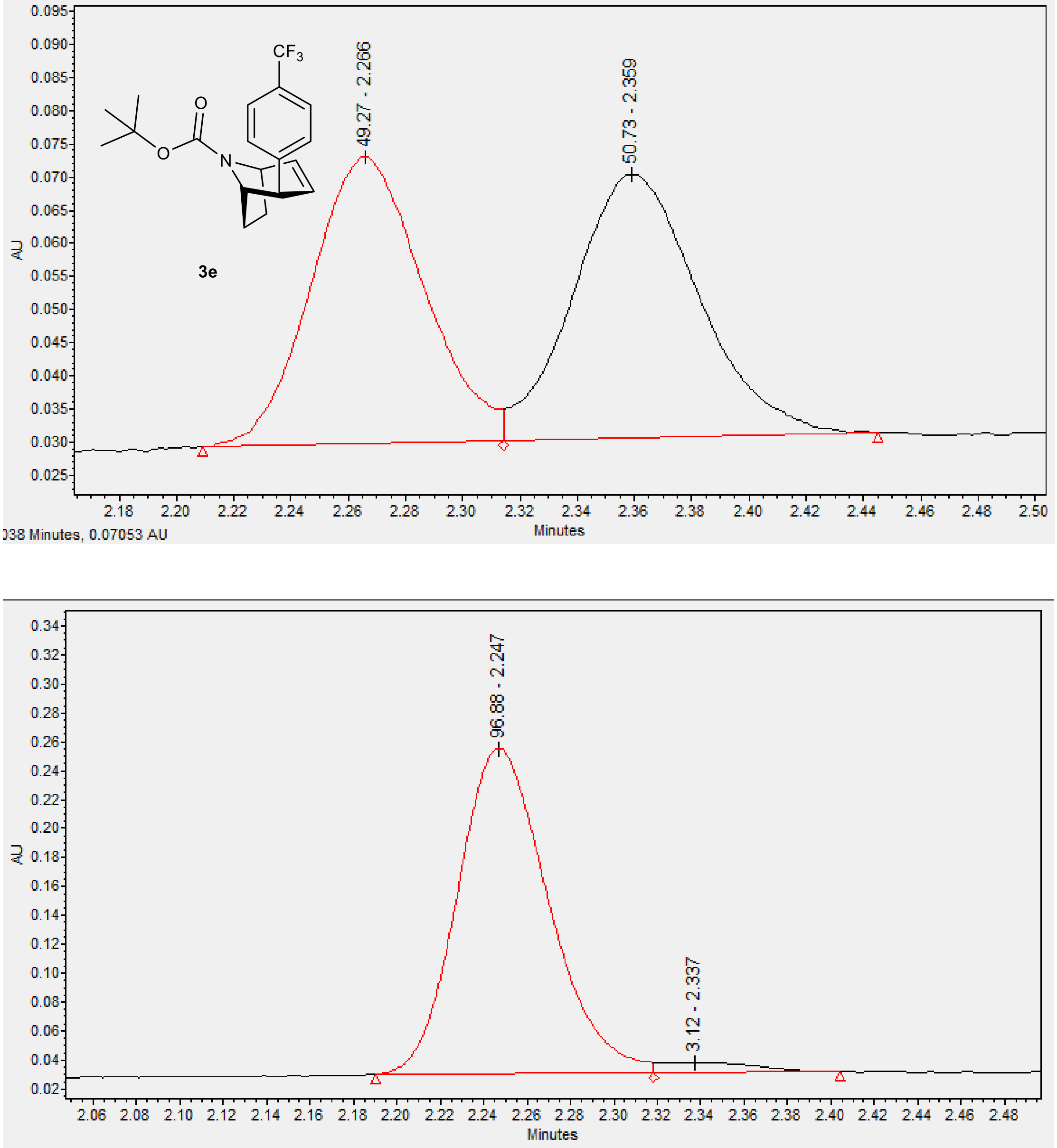

Figure S37. SFC trace of racemic (±)-**3e** and enantioenriched (–)-**3e**.

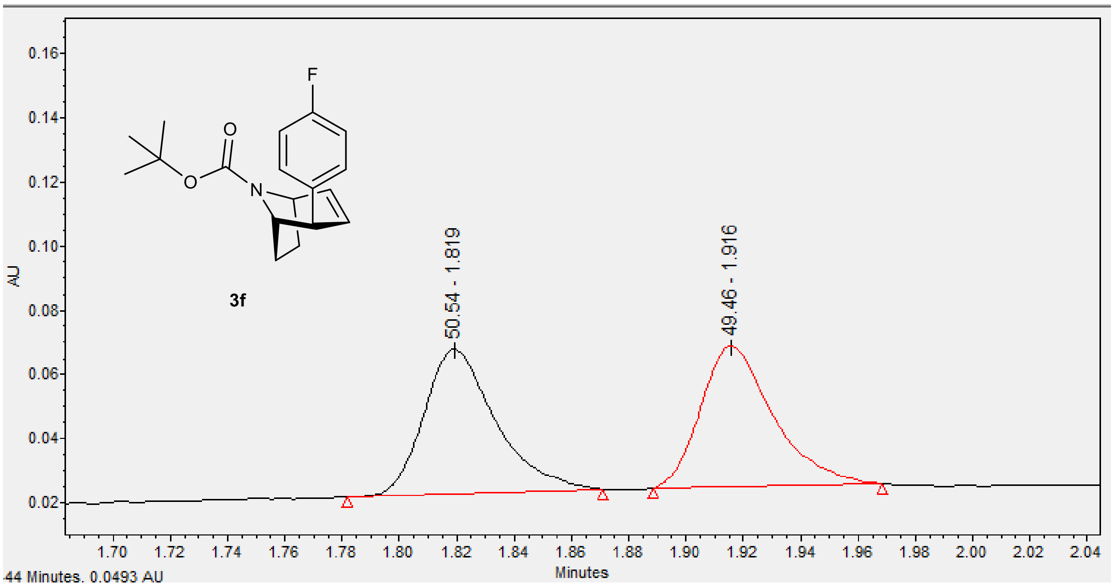

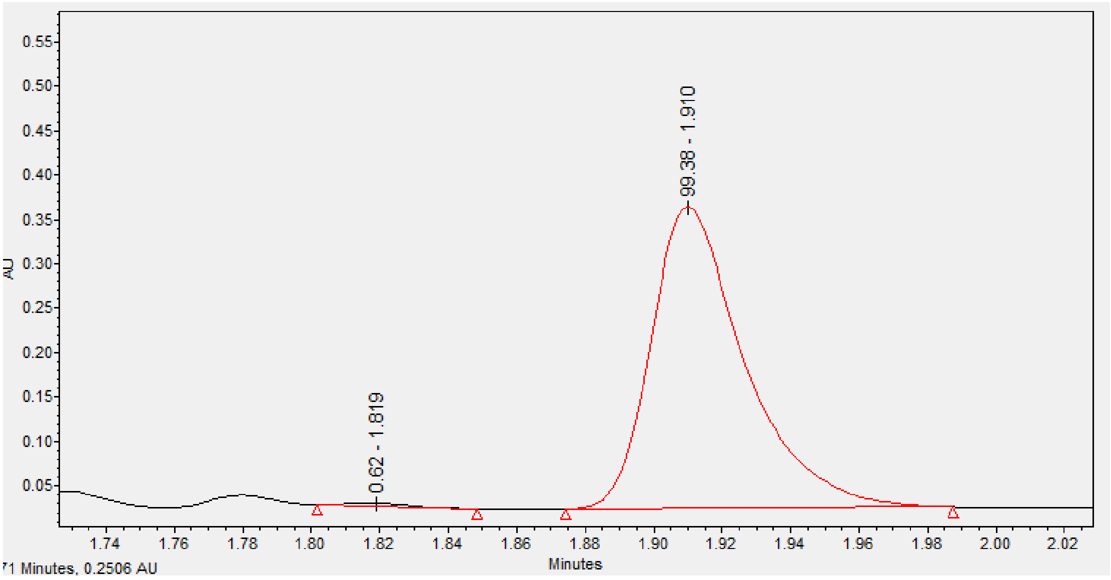

Figure S38. SFC trace of racemic (±)-**3f** and enantioenriched (–)-**3f**.

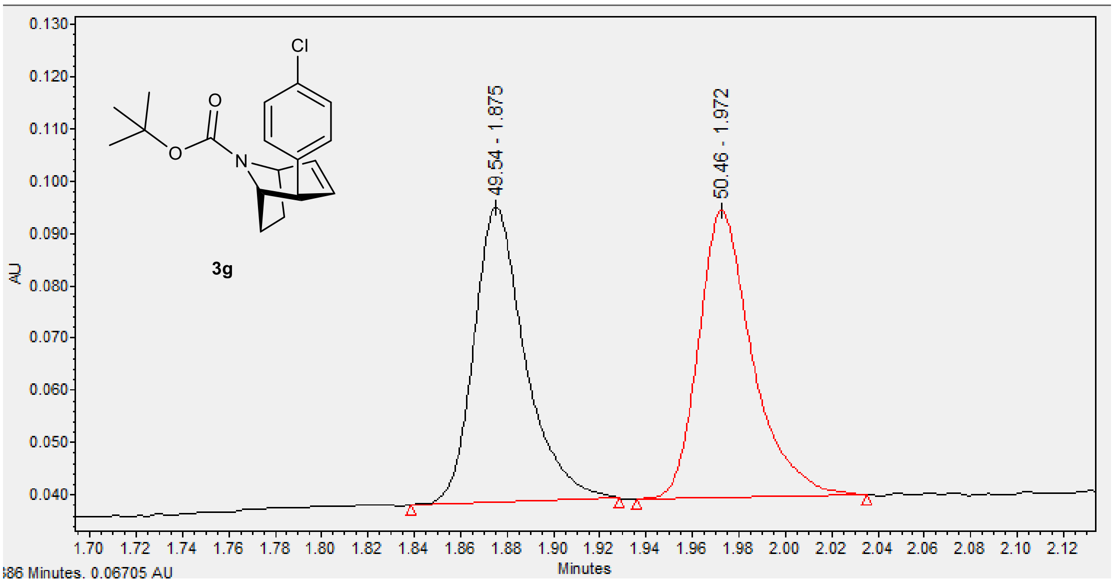

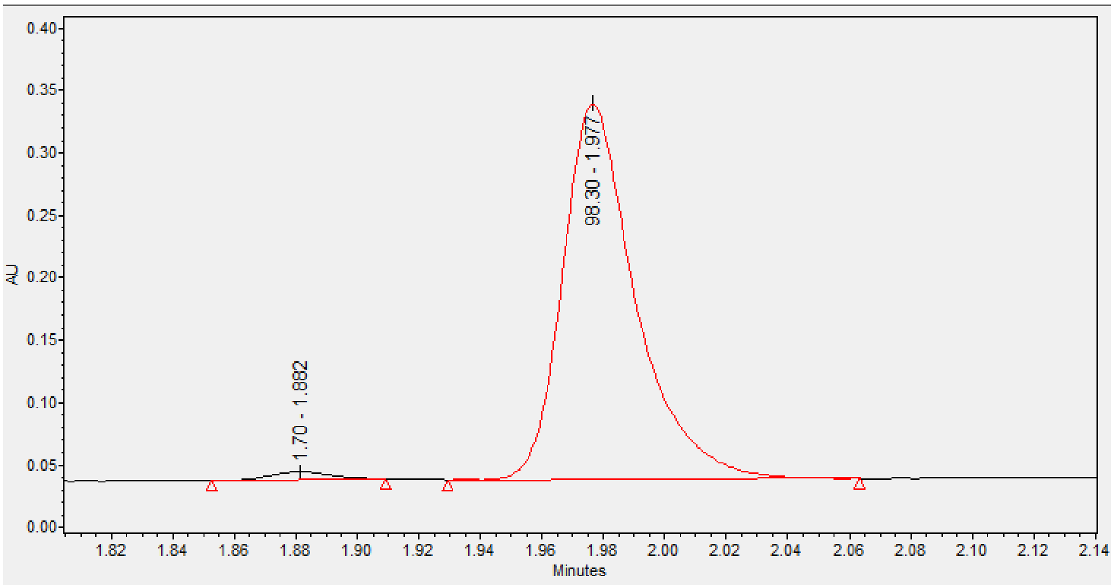

Figure S39. SFC trace of racemic (±)-**3g** and enantioenriched (–)-**3g**.

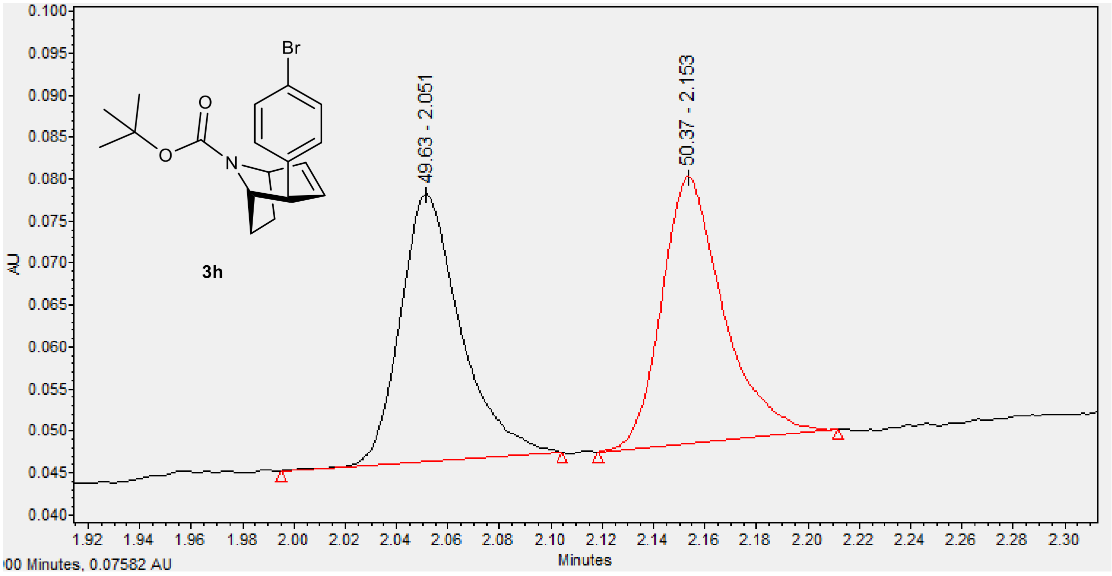

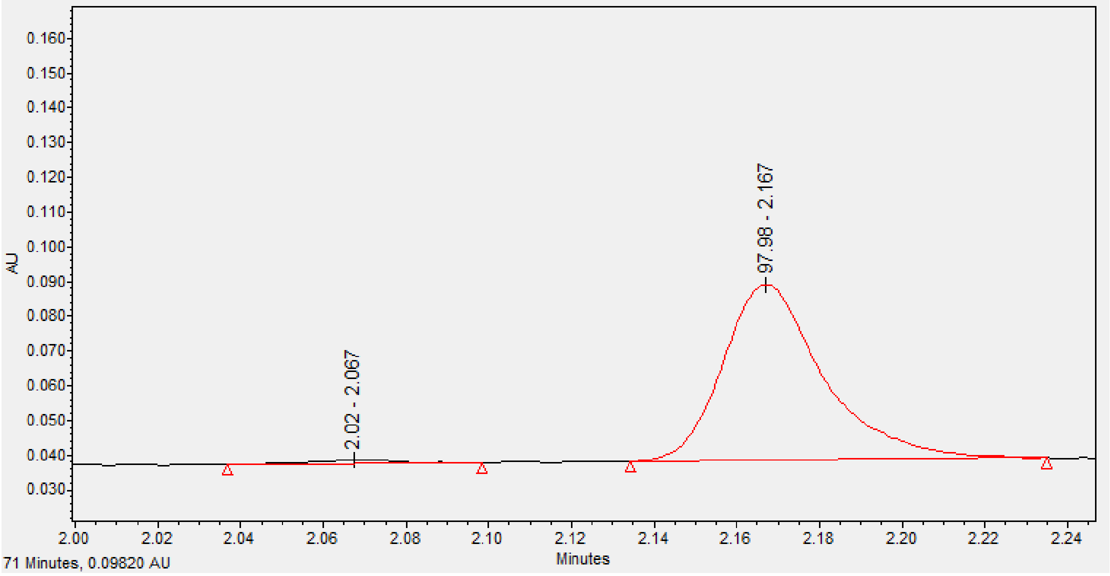

Figure S40. SFC trace of racemic (±)-**3h** and enantioenriched (–)-**3h**.

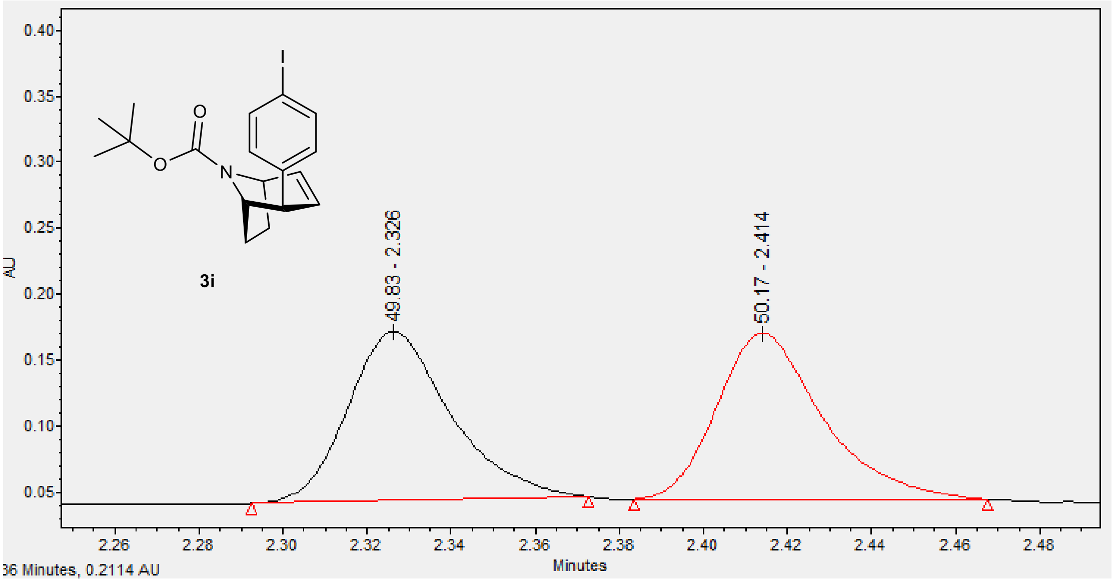

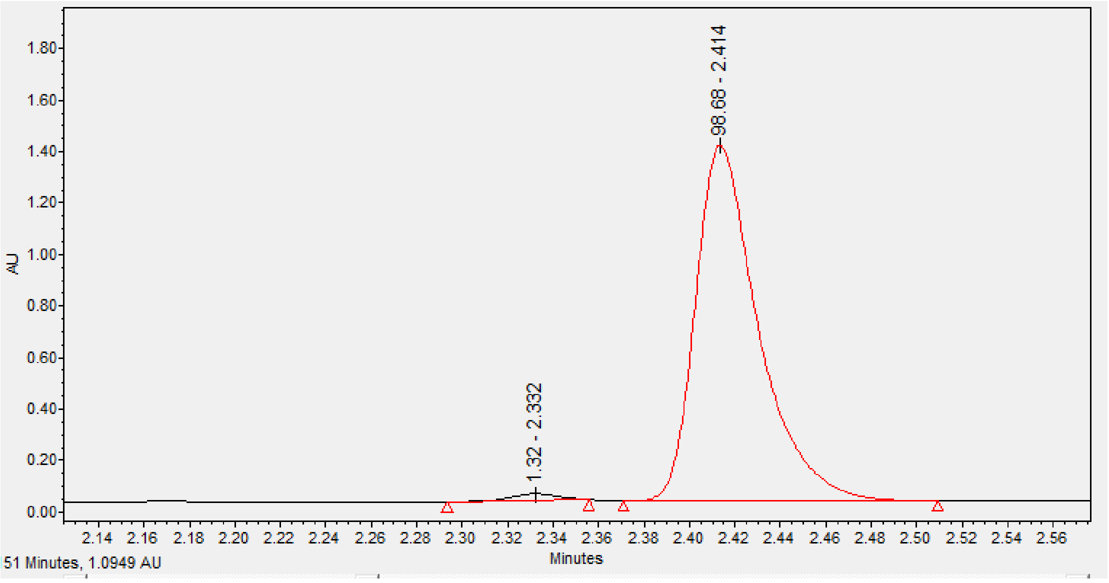

Figure S41. SFC trace of racemic (±)-**3i** and enantioenriched (–)-**3i**.

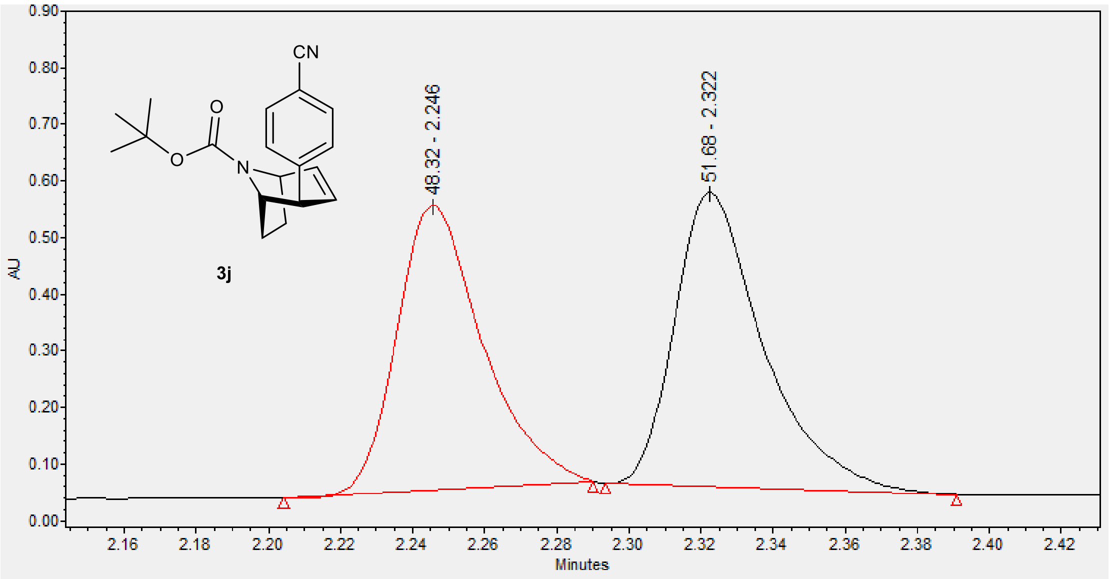

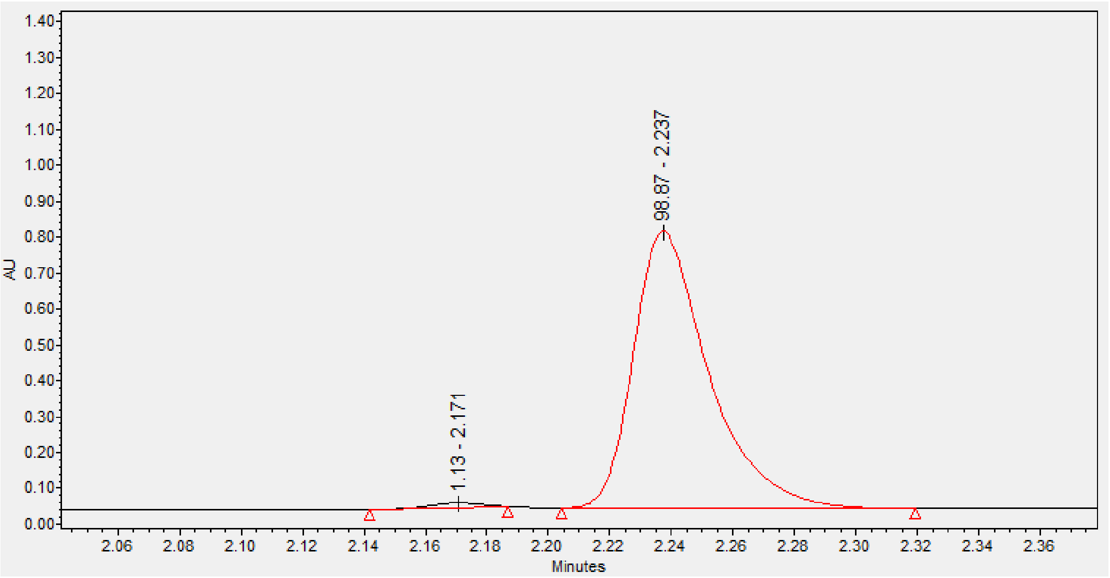

Figure S42. SFC trace of racemic (±)-**3j** and enantioenriched (–)-**3j**.

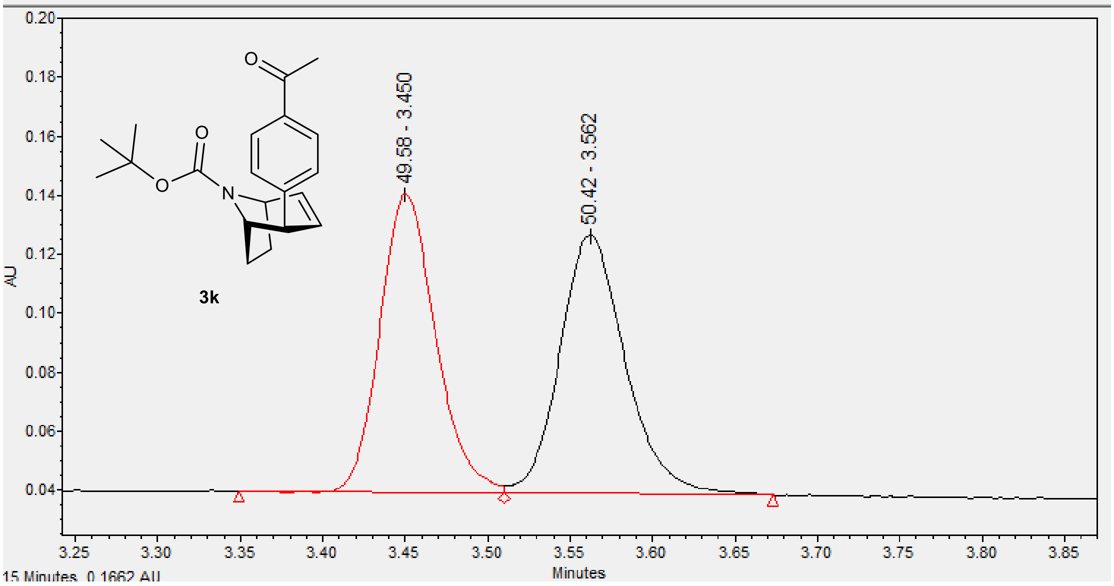

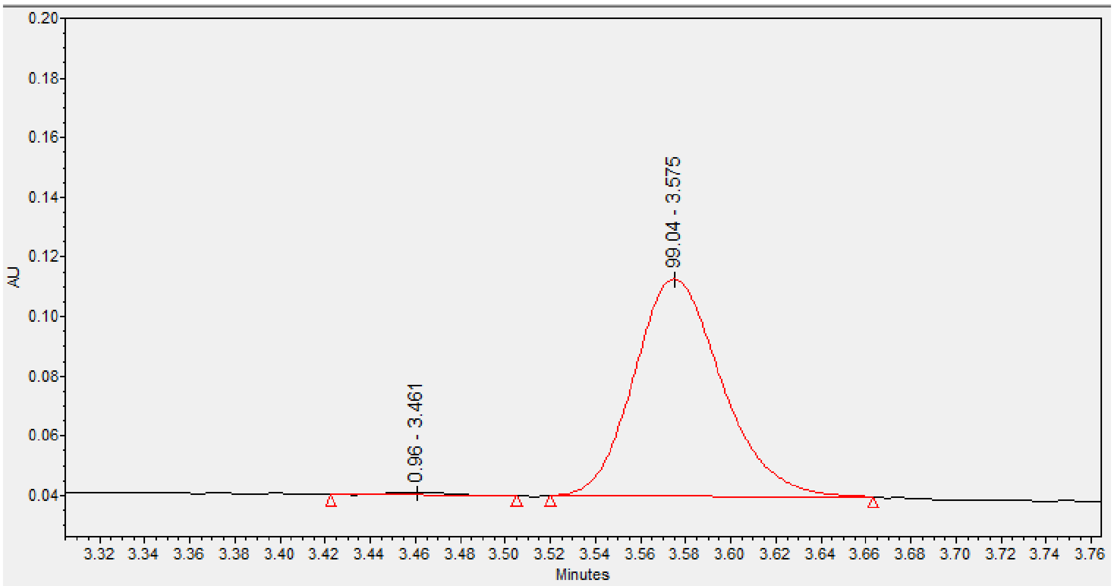

Figure S43. SFC trace of racemic (±)-**3k** and enantioenriched (–)-**3k**.

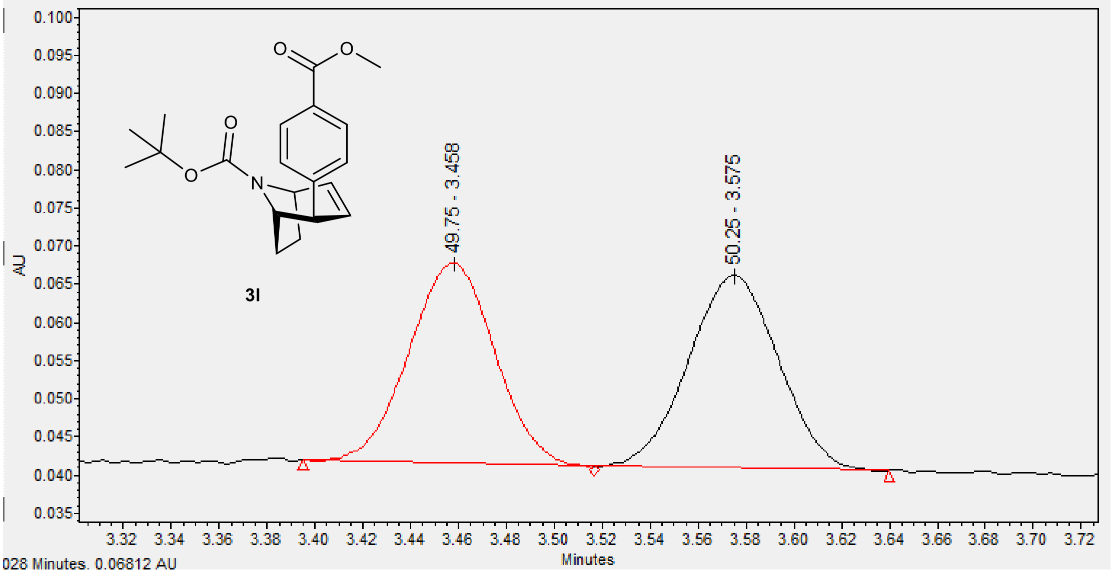

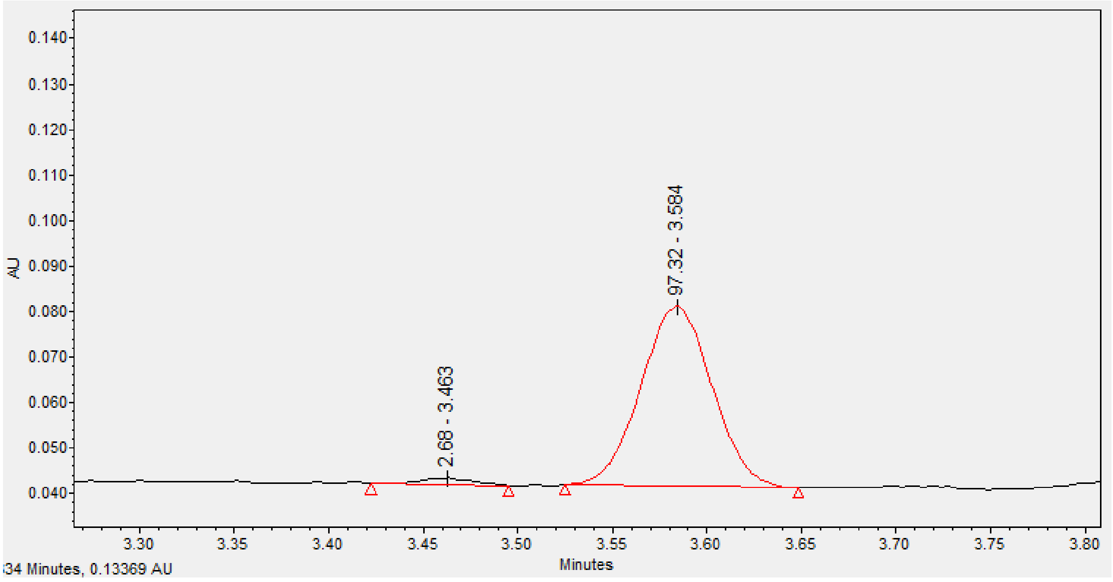

Figure S44. SFC trace of racemic (±)-**3l** and enantioenriched (–)-**3l**.

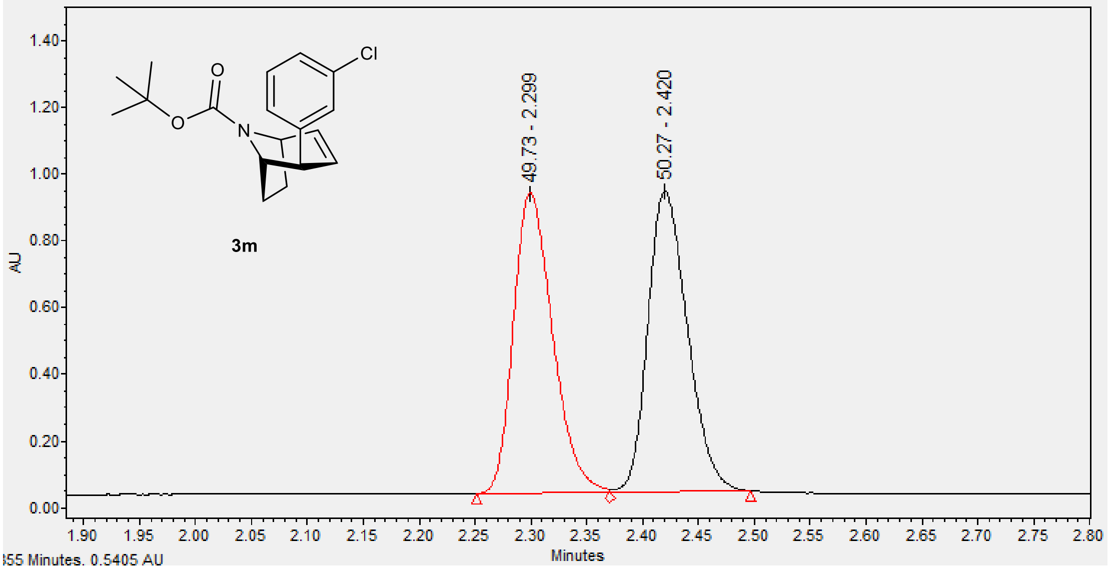

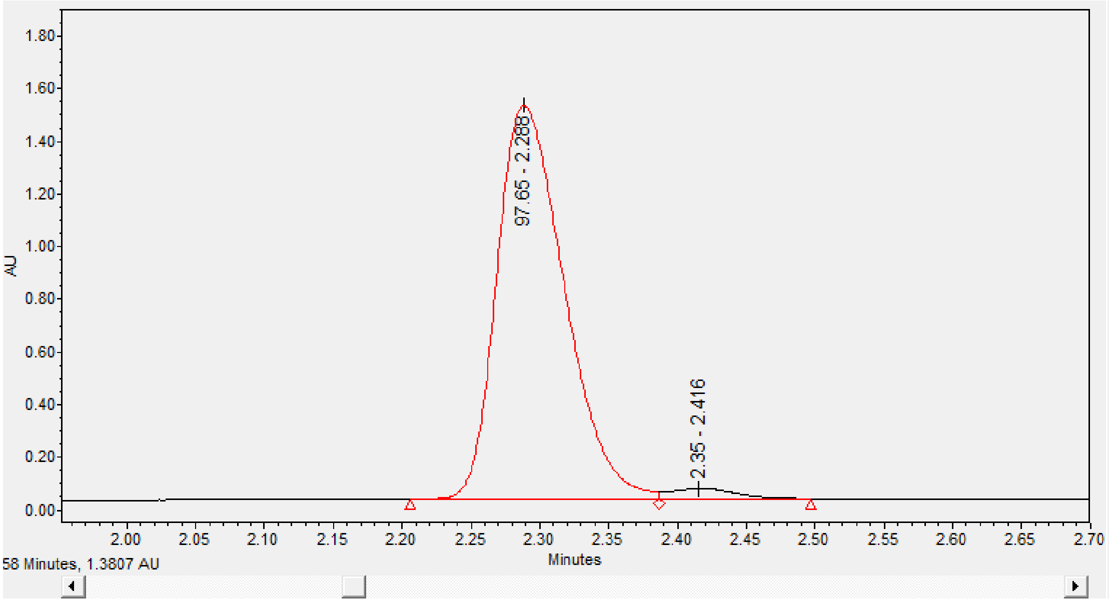

Figure S45. SFC trace of racemic (±)-**3m** and enantioenriched (–)-**3m**.

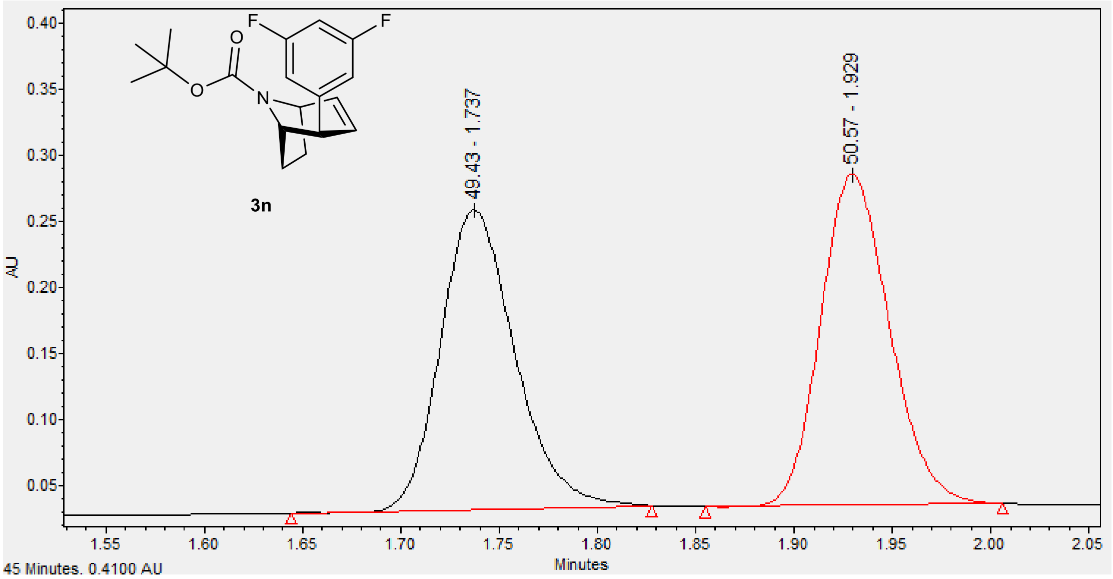

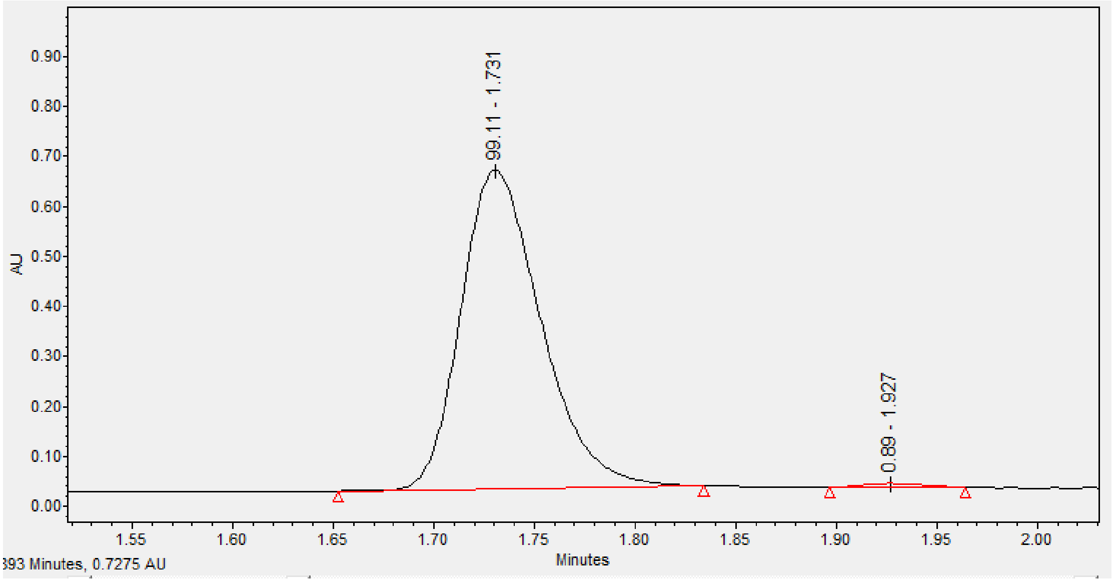

Figure S46. SFC trace of racemic (±)-**3n** and enantioenriched (–)-**3n**.

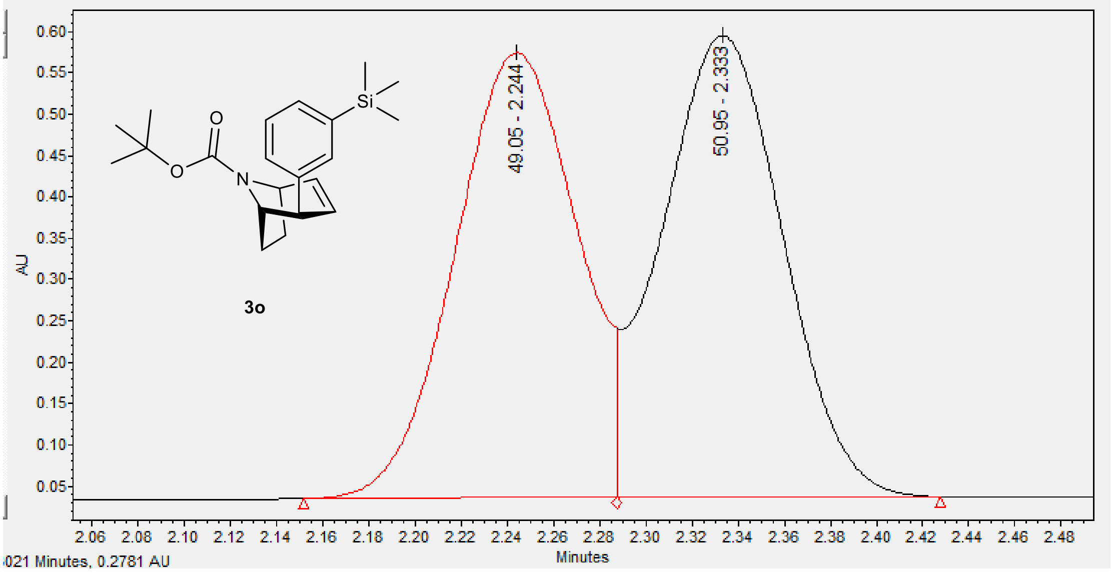

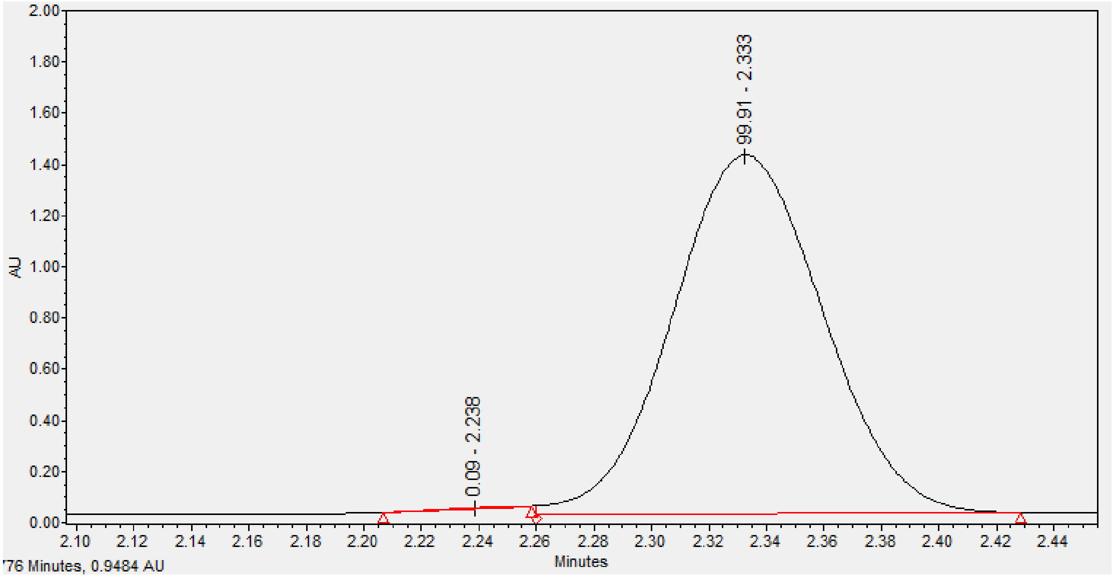

Figure S47. SFC trace of racemic (±)-**3o** and enantioenriched (–)-**3o**.

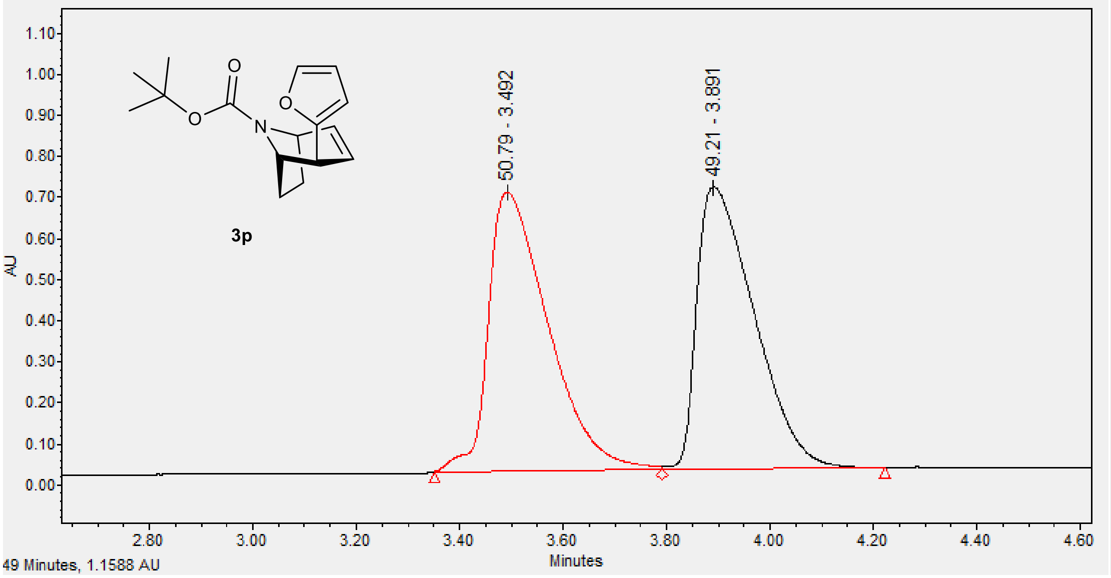

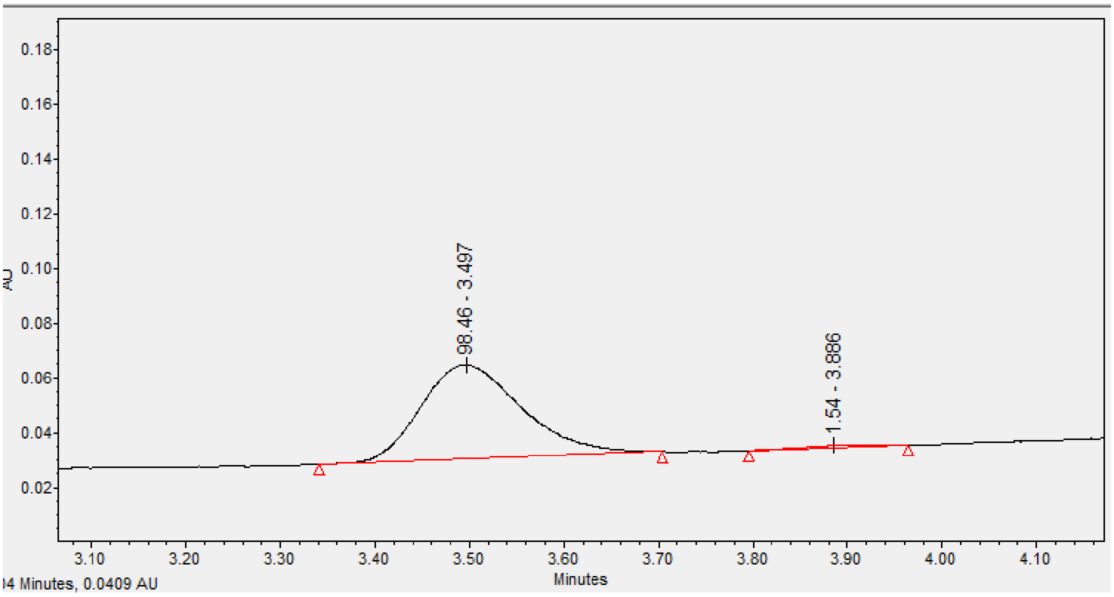

Figure S48. SFC trace of racemic (±)-**3p** and enantioenriched (–)-**3p**.

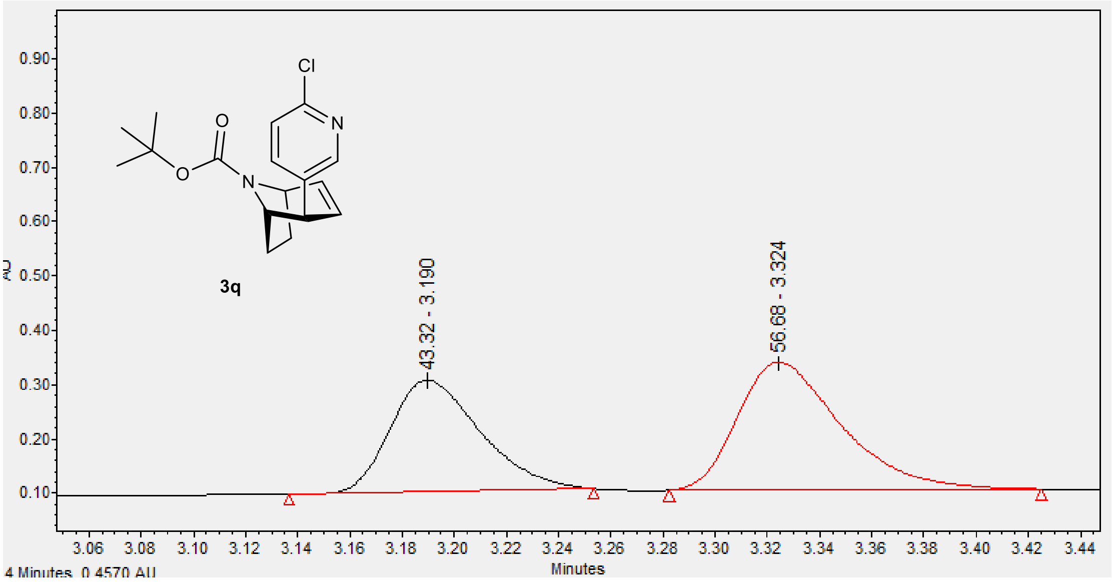

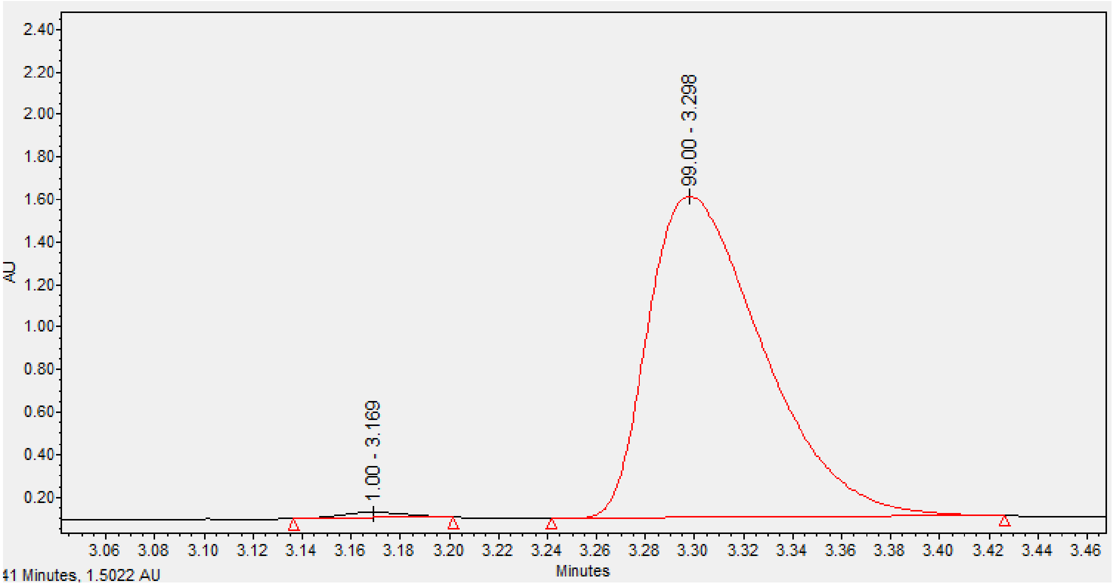

Figure S49. SFC trace of racemic (±)-**3q** and enantioenriched (–)-**3q**.

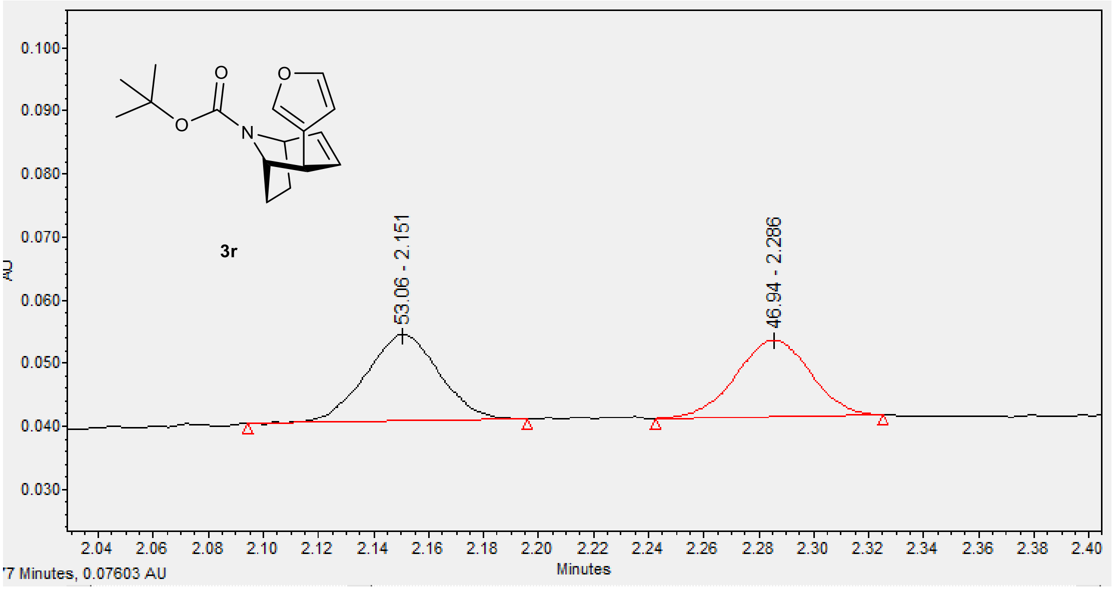

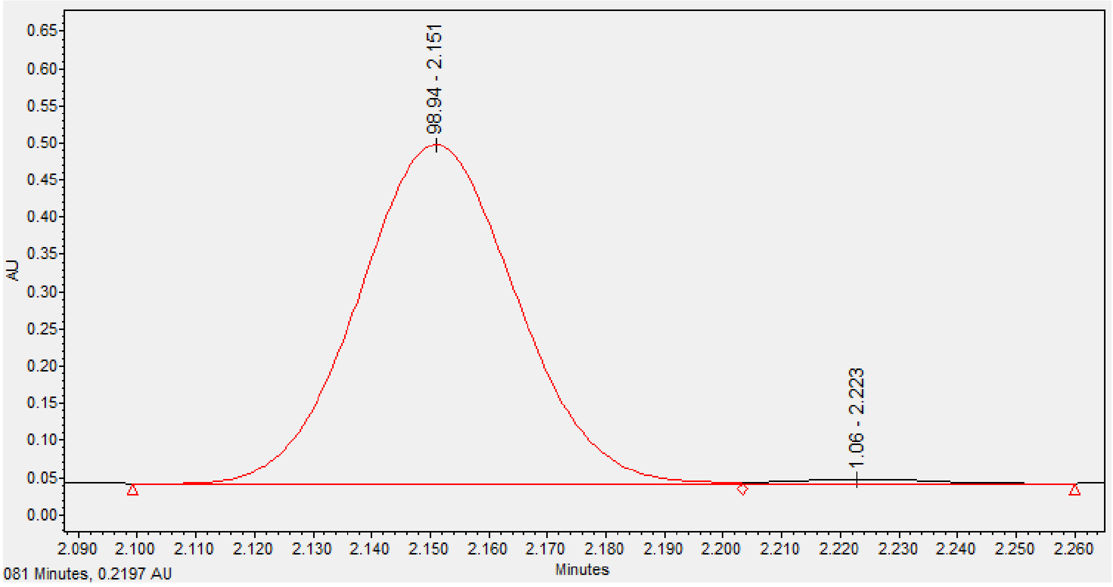

Figure S50. SFC trace of racemic (±)-**3r** and enantioenriched (–)-**3r**.

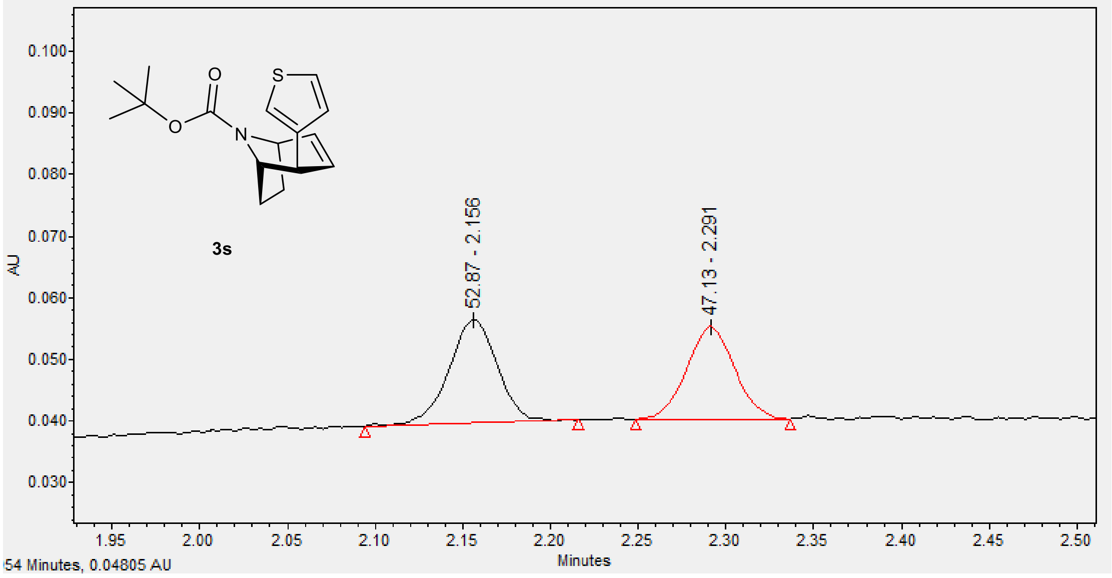

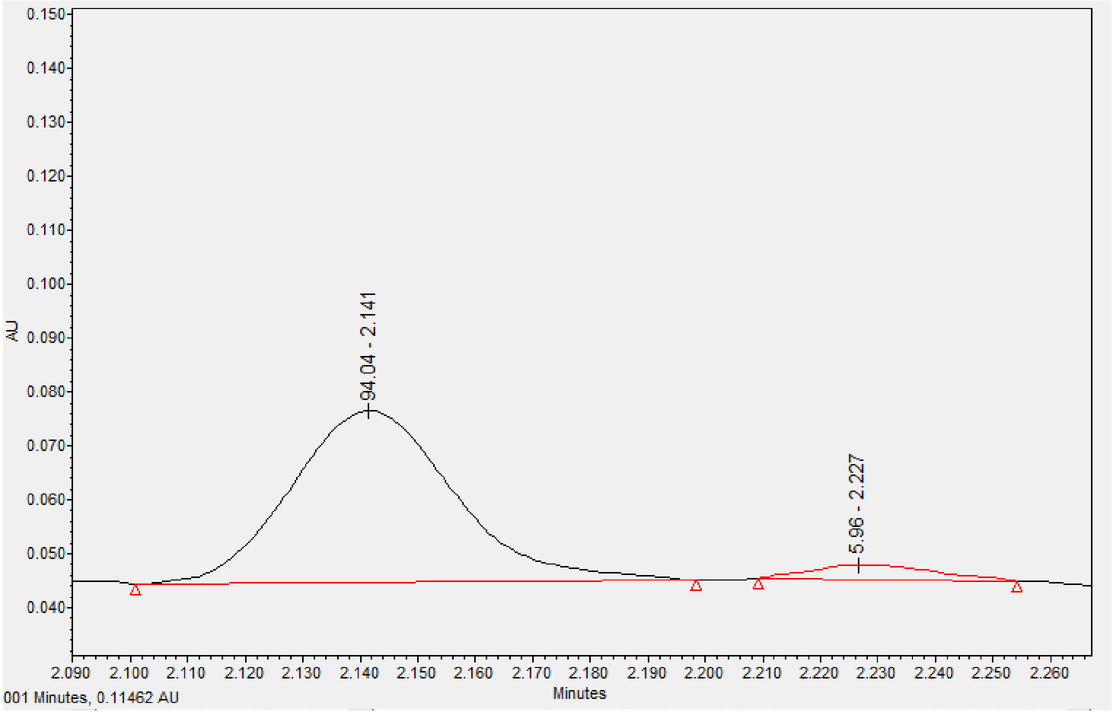

Figure S51. SFC trace of racemic (±)-**3s** and enantioenriched (–)-**3s**.

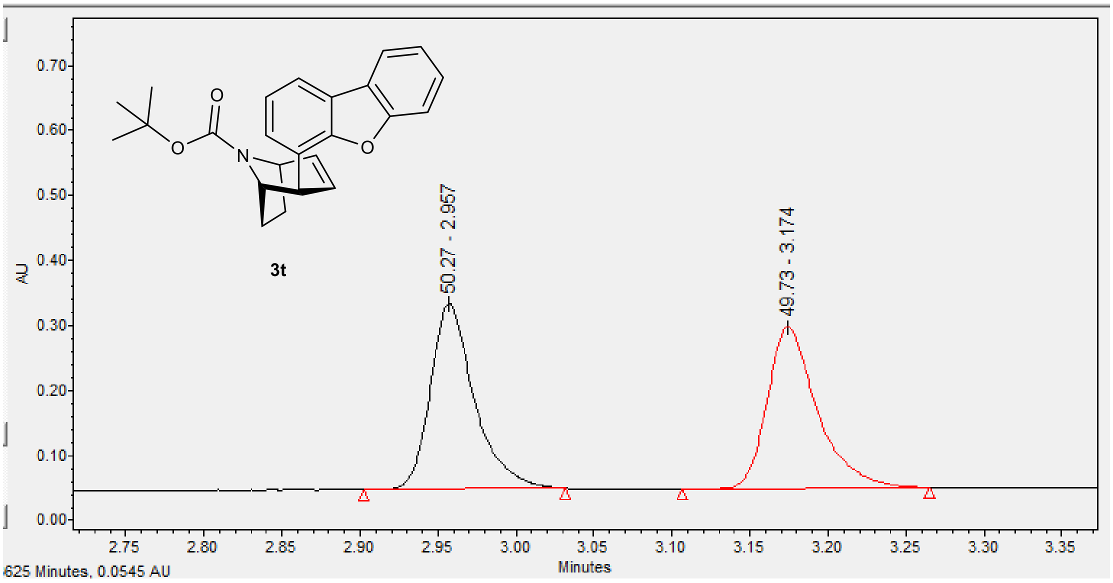

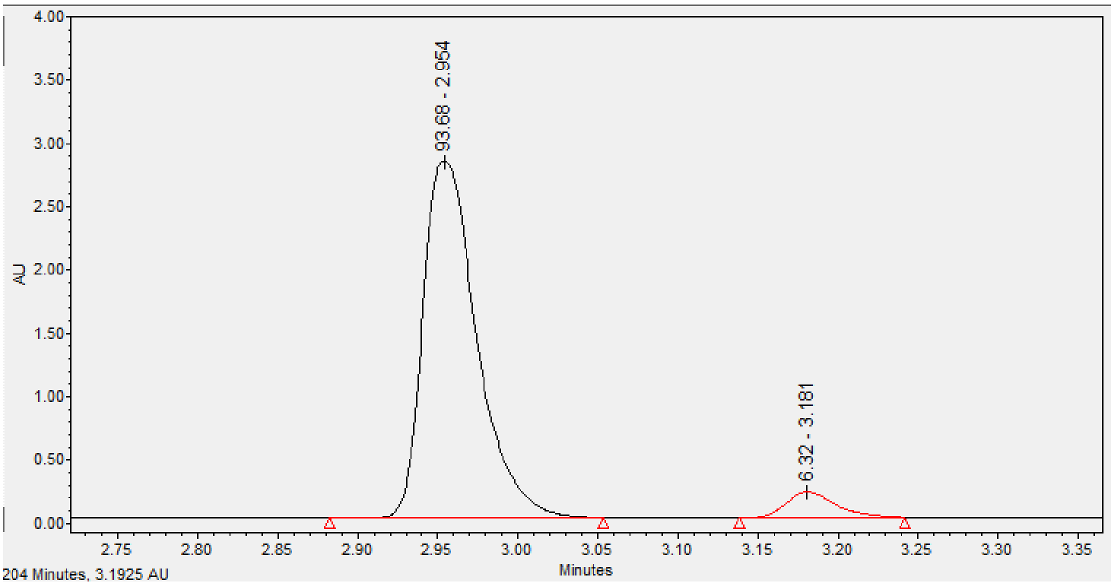

Figure S52. SFC trace of racemic (±)-**3t** and enantioenriched (–)-**3t**.

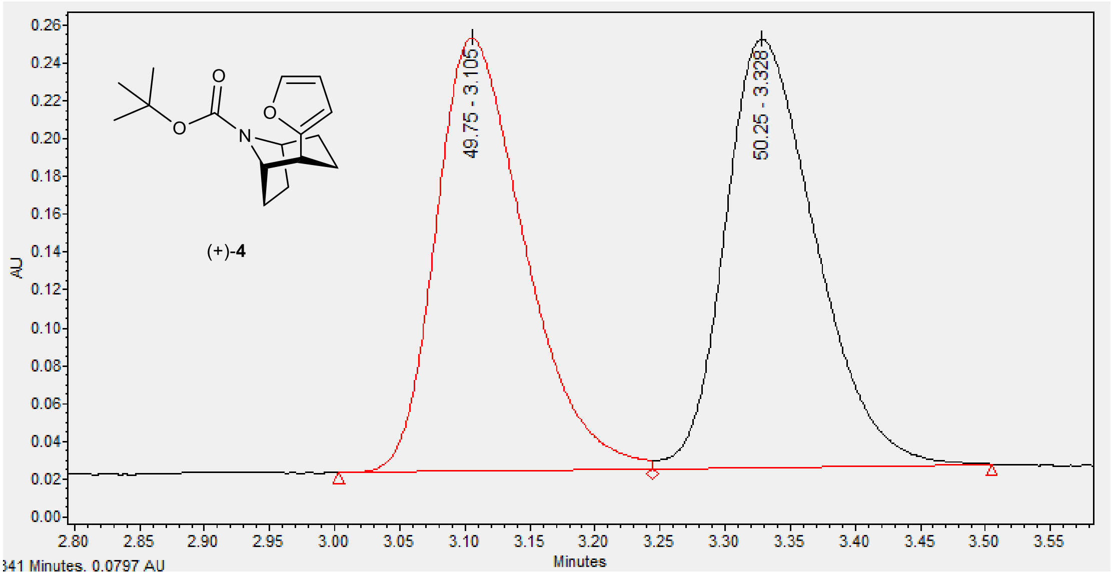

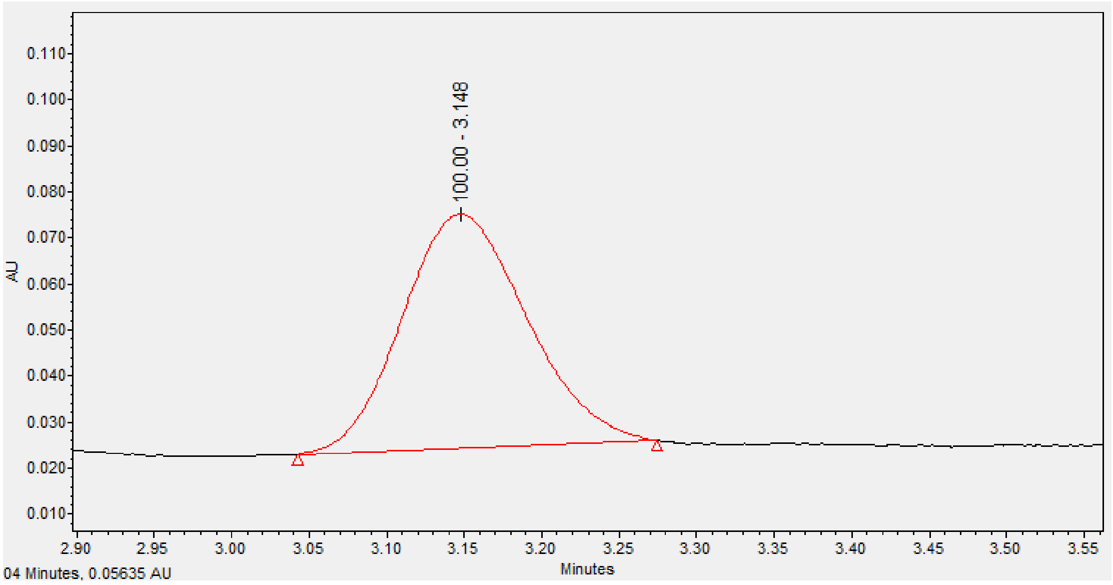

Figure S53. SFC trace of racemic (±)-**4** and enantioenriched (+)-**4**.

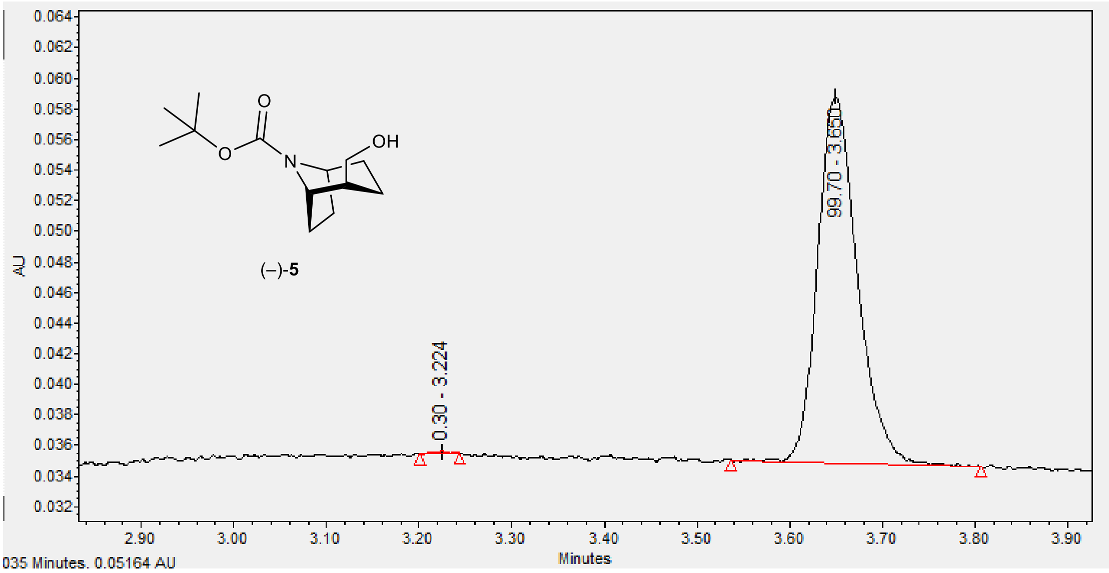

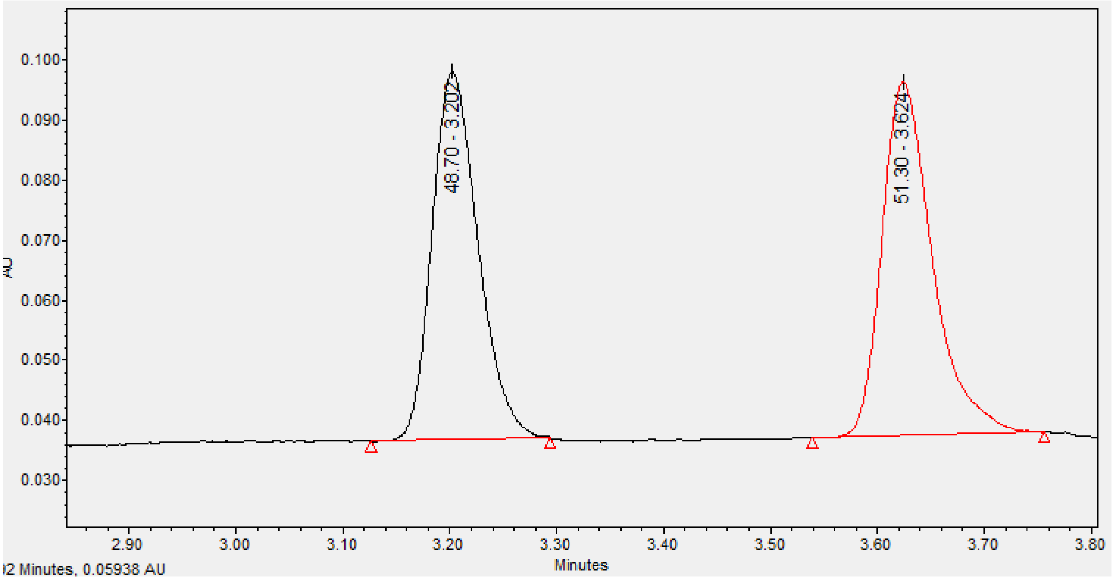

Figure S54. SFC trace of racemic (±)-**5** and enantioenriched (–)-**5**.

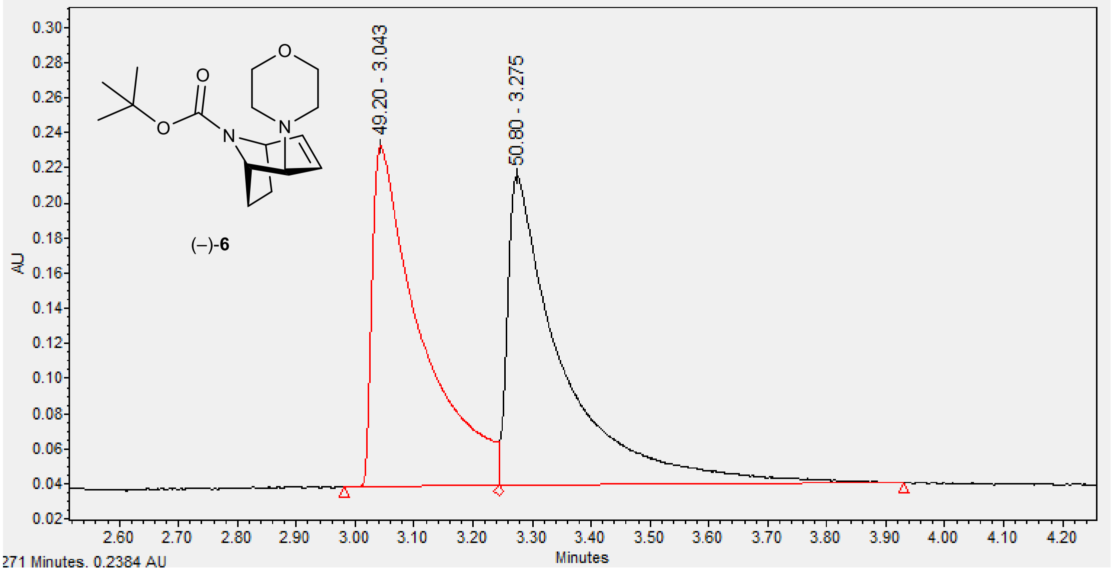

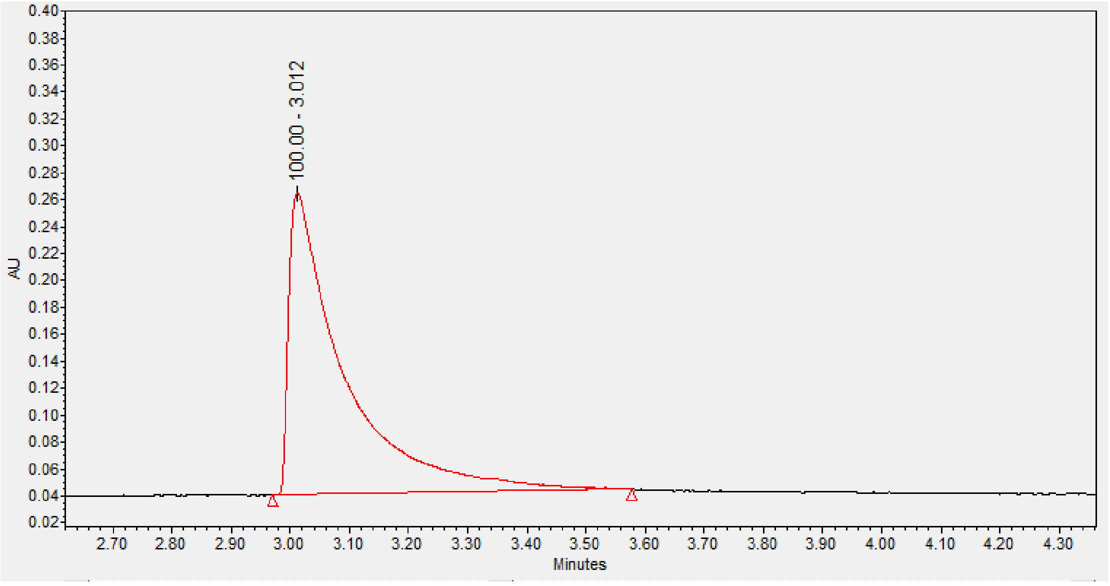

Figure S55. SFC trace of racemic (±)-**6** and enantioenriched (–)-**6**.

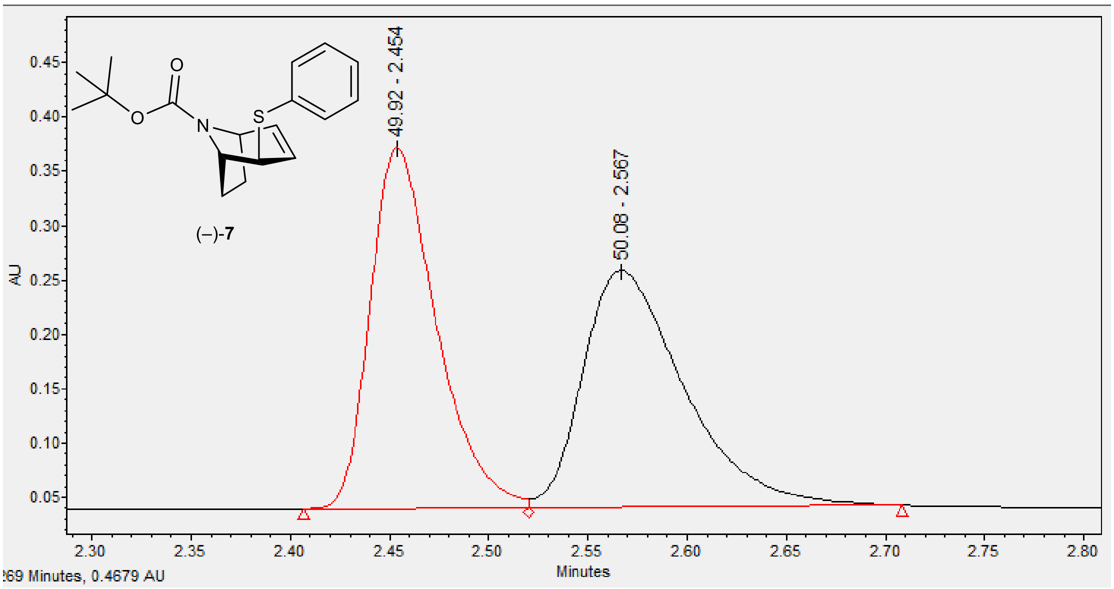

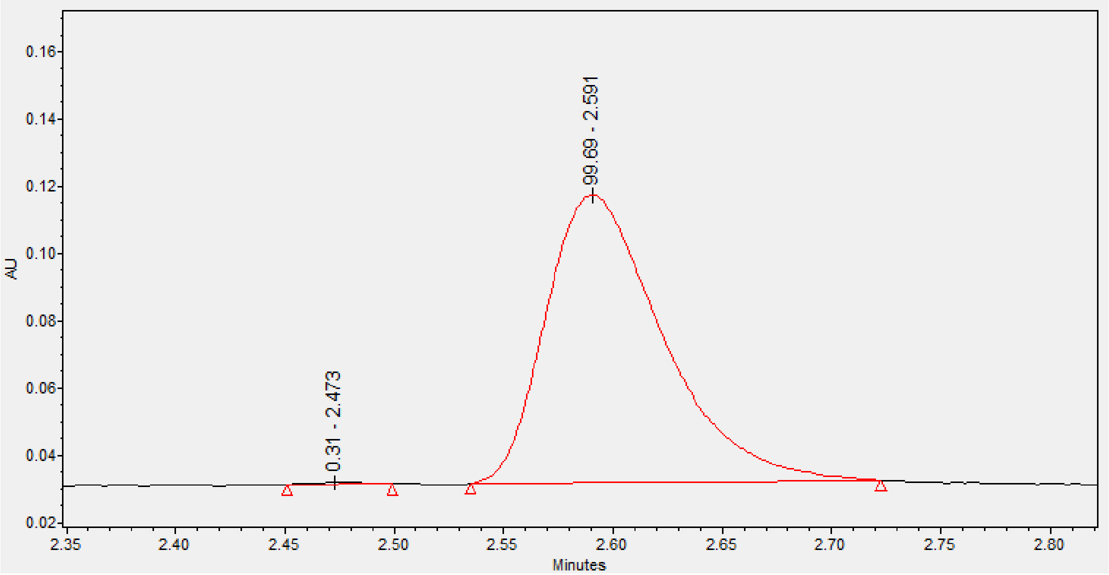

Figure S56. SFC trace of racemic (±)-**7** and enantioenriched (–)-**7**.

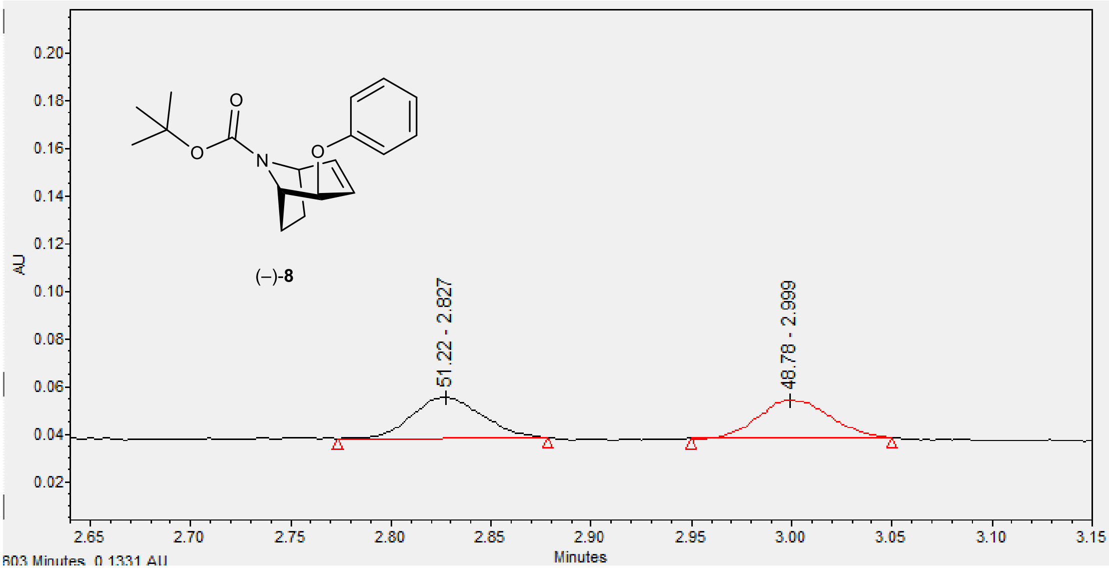

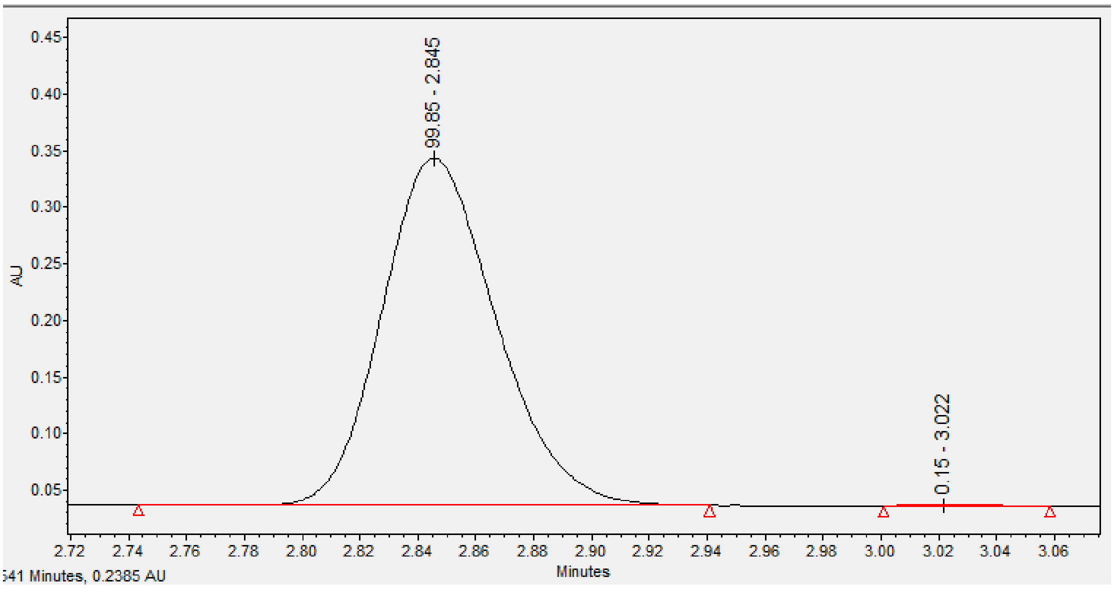

Figure S57. SFC trace of racemic (±)-**8** and enantioenriched (–)-**8**.

5. X-ray Crystallography Data

Low temperature single crystal X-ray diffraction data were collected using a (Rigaku) Oxford Diffraction SuperNova diffractometer. Raw frame data were reduced using CrysAlisPro and the structures were solved using 'Superflip' [10] before refinement with CRYSTALS as per the SI (CIF). [11, 12] Crystallographic data have been deposited with the Cambridge Crystallographic Data Centre as supplementary publication no. CCDC 2156812, 2156553, 2156805 and can be obtained via www. ccdc.cam.ac.uk/data_request/cif.

Table S4. Crystal data and structure refinement for **(+)-1a**.

CCDC number	2156812
Temperature	150 K
Wavelength	1.54184 Å
Crystal system / Space group	Orthorhombic / $P2_12_12_1$
Unit cell dimensions	a = 7.16900(10) Å α = 90°.
	b = 10.31260(10) Åβ = 90°.
	c = 17.30370(10) Åγ = 90°.
Volume	1279.28(2) Å^3
Z	4
Independent reflections	2649 [R(int) = 0.032]
Data / restraints / parameters	2649 / 0 / 147
Goodness-of-fit on F^2	1.0129
Final R indices [I>2sigma(I)]	R1 = 0.0202, wR2 = 0.0542
R indices (all data)	R1 = 0.0204, wR2 = 0.0544
Absolute structure parameter	-0.003(3)
Extinction coefficient	41(4)

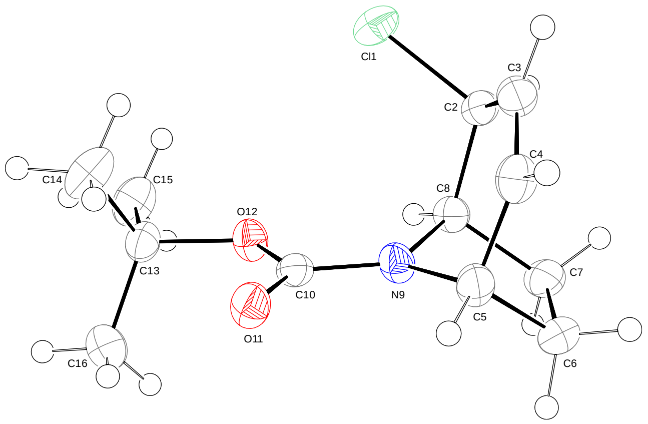

Table S5. Crystal data and structure refinement for **3p**.

CCDC number	2156553
Temperature	150 K
Wavelength	1.54184 Å
Crystal system / Space group	Monoclinic / $P2_1$
Unit cell dimensions	a = 8.00800(10) Å α= 90°.
	b = 16.0984(3) Å β = 103.3911(18)°.
	c = 11.7605(2) Å γ = 90°.
Volume	1474.90(4) Å^3
Z	4
Independent reflections	6078 [R(int) = 0.041]
Data / restraints / parameters	6076 / 1 / 363
Goodness-of-fit on F^2	1.0043
Final R indices [I>2sigma(I)]	R1 = 0.0345, wR2 = 0.0833
R indices (all data)	R1 = 0.0376, wR2 = 0.0868

Absolute structure parameter	0.01(8)
Extinction coefficient	13(3)

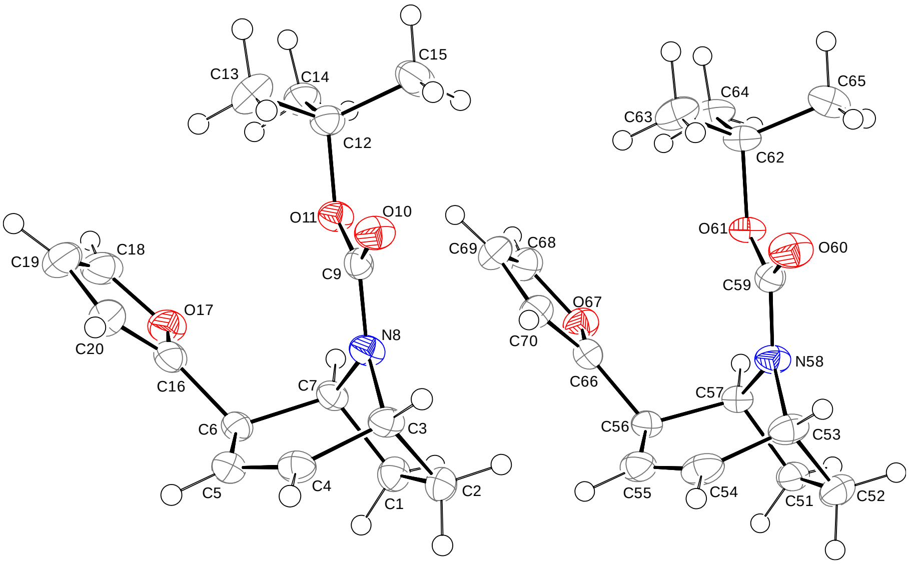

Table S6. Crystal data and structure refinement for (–)-**6**.

CCDC number	2156805
Temperature	150 K
Wavelength	1.54184 Å
Crystal system / Space group	Orthorhombic / $P2_12_12_1$
Unit cell dimensions	a = 6.13100(10) Å α = 90°.
	b = 15.83460(10) Åβ = 90°.
	c = 16.66730(10) Åγ = 90°.
Volume	1618.09(3) Å^3
Z	4
Independent reflections	3363 [R(int) = 0.026]

Data / restraints / parameters	3363 / 0 / 191
Goodness-of-fit on F^2	1.0052
Final R indices [I>2sigma(I)]	R1 = 0.0266, wR2 = 0.0710
R indices (all data)	R1 = 0.0270, wR2 = 0.0715
Absolute structure parameter	-0.03(3)
Largest diff. peak and hole	0.06 and -0.07 e.Å^{-3}

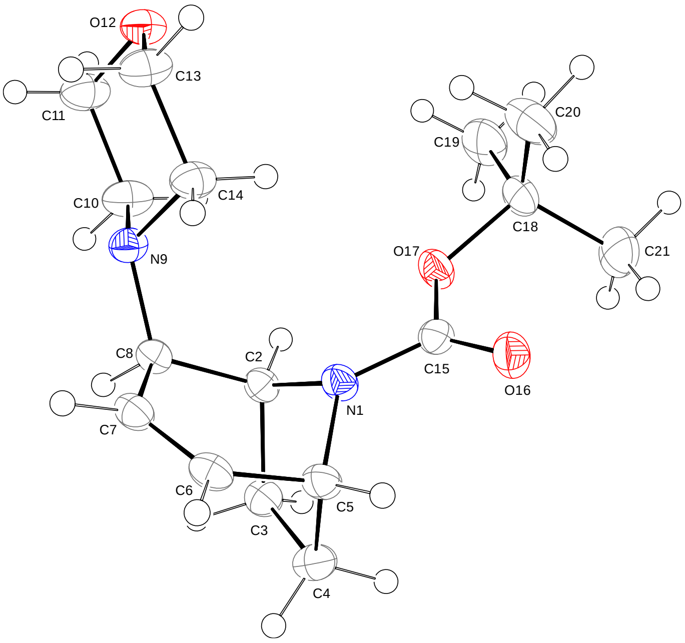

6. Author Contributions

Y.Z. performed all experiments. F.W.G. and S.P.F. conceived the project and supervised the work. K.E.C. performed and analysed single crystal X-ray diffraction experiments. Y.Z., F.W.G. and S.P.F. wrote the manuscript.
