# Supplementary material for: Asymmetric Synthesis of Nortropanes via Rh-Catalyzed Allylic Arylation
Source: ACS Catal. 2022 Jul 12;12(15):8995–9002. doi: 10.1021/acscatal.2c02259 (PMC9361292; doi:10.1021/acscatal.2c02259)

checkCIF/PLATON report

Structure factors have been supplied for datablock(s) 1a, 3p, 6

THIS REPORT IS FOR GUIDANCE ONLY. IF USED AS PART OF A REVIEW PROCEDURE FOR PUBLICATION, IT SHOULD NOT REPLACE THE EXPERTISE OF AN EXPERIENCED CRYSTALLOGRAPHIC REFEREE.

No syntax errors found. CIF dictionary Interpreting this report

Datablock: 1a

```
Bond precision:   C-C = 0.0016 A           Wavelength=1.54184

Cell:               a=7.1690(1)      b=10.3126(1)      c=17.3037(1)
                    alpha=90         beta=90           gamma=90
Temperature:        150 K

                 Calculated                    Reported
Volume           1279.28(2)                    1279.28(2)
Space group      P 21 21 21                    P 21 21 21
Hall group       P 2ac 2ab                     P 2ac 2ab
Moiety formula   C12 H18 Cl N O2               C12 H18 Cl N O2
Sum formula      C12 H18 Cl N O2               C12 H18 Cl N O2
Mr               243.72                        243.73
Dx,g cm-3        1.265                         1.265
Z                4                             4
Mu (mm-1)        2.536                         2.536
F000             520.0                         520.0
F000'            522.79
h,k,lmax         9,12,21                       8,12,21
Nref             2662[ 1553]                   2649
Tmin,Tmax        0.543,0.816                   0.550,0.820
Tmin'            0.493

Correction method= # Reported T Limits: Tmin=0.550 Tmax=0.820
AbsCorr = MULTI-SCAN

Data completeness= 1.71/1.00       Theta(max)= 76.170

R(reflections)= 0.0202( 2629)                     wR2(reflections)=
                                                  0.0544( 2649)
S = 1.013                  Npar= 147
```

The following ALERTS were generated. Each ALERT has the format
test-name_ALERT_alert-type_alert-level.
Click on the hyperlinks for more details of the test.

Alert level G

```
PLAT066_ALERT_1_G Predicted and Reported Tmin&Tmax Range Identical          ? Check
PLAT142_ALERT_4_G s.u. on b - Axis Small or Missing .............    0.00010 Ang.
PLAT143_ALERT_4_G s.u. on c - Axis Small or Missing .............    0.00010 Ang.
PLAT153_ALERT_1_G The s.u.'s on the Cell Axes   are Equal ..(Note)     0.0001 Ang.
PLAT769_ALERT_4_G CIF Embedded explicitly supplied scattering data     Please Note
PLAT791_ALERT_4_G Model has Chirality at C2          (Sohnke SpGr)          S Verify
PLAT791_ALERT_4_G Model has Chirality at C5          (Sohnke SpGr)          S Verify
PLAT791_ALERT_4_G Model has Chirality at C8          (Sohnke SpGr)          R Verify
PLAT802_ALERT_4_G CIF Input Record(s) with more than 80 Characters          1 Info
PLAT912_ALERT_4_G Missing # of FCF Reflections Above STh/L=  0.600          4 Note
```

0 **ALERT level A** = Most likely a serious problem - resolve or explain
0 **ALERT level B** = A potentially serious problem, consider carefully
0 **ALERT level C** = Check. Ensure it is not caused by an omission or oversight
10 **ALERT level G** = General information/check it is not something unexpected

2 ALERT type 1 CIF construction/syntax error, inconsistent or missing data
0 ALERT type 2 Indicator that the structure model may be wrong or deficient
0 ALERT type 3 Indicator that the structure quality may be low
8 ALERT type 4 Improvement, methodology, query or suggestion
0 ALERT type 5 Informative message, check

Datablock: 3p

Bond precision: C-C = 0.0023 A Wavelength=1.54184

Cell:	a=8.0080(1)	b=16.0984(3)	c=11.7605(2)
	alpha=90	beta=103.3911(18)	gamma=90
Temperature:	150 K		

	Calculated	Reported
Volume	1474.90(4)	1474.90(4)
Space group	P 21	P 21
Hall group	P 2yb	P 2yb
Moiety formula	C16 H21 N O3	C16 H21 N O3
Sum formula	C16 H21 N O3	C16 H21 N O3
Mr	275.34	275.35
Dx,g cm-3	1.240	1.240
Z	4	4
Mu (mm-1)	0.688	0.688
F000	592.0	592.0
F000'	593.79	
h,k,lmax	10,20,14	9,20,14
Nref	6164[3195]	6078
Tmin,Tmax	0.834,0.980	0.700,0.980
Tmin'	0.775	

Correction method= # Reported T Limits: Tmin=0.700 Tmax=0.980
AbsCorr = MULTI-SCAN

Data completeness= 1.90/0.99 Theta(max)= 76.133

R(reflections)= 0.0345(5707) wR2(reflections)= 0.0868(6076)

S = 1.004 Npar= 363

The following ALERTS were generated. Each ALERT has the format
test-name_ALERT_alert-type_alert-level.
Click on the hyperlinks for more details of the test.

Alert level G

```
PLAT398_ALERT_2_G Deviating  C-O-C   Angle From 120 for O17      .      106.0 Degree
PLAT398_ALERT_2_G Deviating  C-O-C   Angle From 120 for O67      .      106.7 Degree
PLAT769_ALERT_4_G CIF Embedded explicitly supplied scattering data     Please Note
PLAT791_ALERT_4_G Model has Chirality at C3         (Sohnke SpGr)          R Verify
PLAT791_ALERT_4_G Model has Chirality at C6         (Sohnke SpGr)          R Verify
PLAT791_ALERT_4_G Model has Chirality at C7         (Sohnke SpGr)          S Verify
PLAT791_ALERT_4_G Model has Chirality at C53        (Sohnke SpGr)          R Verify
PLAT791_ALERT_4_G Model has Chirality at C56        (Sohnke SpGr)          R Verify
PLAT791_ALERT_4_G Model has Chirality at C57        (Sohnke SpGr)          S Verify
PLAT912_ALERT_4_G Missing # of FCF Reflections Above STh/L=  0.600        13 Note
PLAT960_ALERT_3_G Number of Intensities with I  <   - 2*sig(I) ...         1 Check
```

---

0 **ALERT level A** = Most likely a serious problem - resolve or explain
0 **ALERT level B** = A potentially serious problem, consider carefully
0 **ALERT level C** = Check. Ensure it is not caused by an omission or oversight
11 **ALERT level G** = General information/check it is not something unexpected

```
0 ALERT type 1 CIF construction/syntax error, inconsistent or missing data
2 ALERT type 2 Indicator that the structure model may be wrong or deficient
1 ALERT type 3 Indicator that the structure quality may be low
8 ALERT type 4 Improvement, methodology, query or suggestion
0 ALERT type 5 Informative message, check
```

Datablock: 6

Bond precision: C-C = 0.0015 A Wavelength=1.54184

Cell:	a=6.1310(1)	b=15.8346(1)	c=16.6673(1)
	alpha=90	beta=90	gamma=90
Temperature:	150 K		

	Calculated	Reported
Volume	1618.09(3)	1618.09(3)
Space group	P 21 21 21	P 21 21 21
Hall group	P 2ac 2ab	P 2ac 2ab
Moiety formula	C16 H26 N2 O3	C16 H26 N2 O3
Sum formula	C16 H26 N2 O3	C16 H26 N2 O3
Mr	294.39	294.39
Dx,g cm-3	1.209	1.208
Z	4	4
Mu (mm-1)	0.671	0.671
F000	640.0	640.0
F000’	641.89	
h,k,lmax	7,19,20	7,19,20
Nref	3380[1964]	3363
Tmin,Tmax	0.851,0.880	0.810,0.880
Tmin’	0.834	

Correction method= # Reported T Limits: Tmin=0.810 Tmax=0.880
AbsCorr = MULTI-SCAN

Data completeness= 1.71/0.99 Theta(max)= 75.975

R(reflections)= 0.0266(3309) wR2(reflections)= 0.0715(3363)

S = 1.005 Npar= 191

The following ALERTS were generated. Each ALERT has the format
test-name_ALERT_alert-type_alert-level.
Click on the hyperlinks for more details of the test.

Alert level C

```
PLAT911_ALERT_3_C Missing FCF Refl Between Thmin & STh/L=    0.600          2 Report
```

Alert level G

```
PLAT142_ALERT_4_G s.u. on b - Axis Small or Missing .............    0.00010 Ang.
PLAT143_ALERT_4_G s.u. on c - Axis Small or Missing .............    0.00010 Ang.
PLAT153_ALERT_1_G The s.u.'s on the Cell Axes   are Equal ..(Note)     0.0001 Ang.
PLAT398_ALERT_2_G Deviating  C-O-C   Angle From 120 for O12      .      109.9 Degree
PLAT769_ALERT_4_G CIF Embedded explicitly supplied scattering data     Please Note
PLAT791_ALERT_4_G Model has Chirality at C2          (Sohnke SpGr)          S Verify
PLAT791_ALERT_4_G Model has Chirality at C5          (Sohnke SpGr)          R Verify
PLAT791_ALERT_4_G Model has Chirality at C8          (Sohnke SpGr)          R Verify
PLAT912_ALERT_4_G Missing # of FCF Reflections Above STh/L=  0.600          1 Note
```

0 **ALERT level A** = Most likely a serious problem - resolve or explain
0 **ALERT level B** = A potentially serious problem, consider carefully
1 **ALERT level C** = Check. Ensure it is not caused by an omission or oversight
9 **ALERT level G** = General information/check it is not something unexpected

1 ALERT type 1 CIF construction/syntax error, inconsistent or missing data
1 ALERT type 2 Indicator that the structure model may be wrong or deficient
1 ALERT type 3 Indicator that the structure quality may be low
7 ALERT type 4 Improvement, methodology, query or suggestion
0 ALERT type 5 Informative message, check

Publication of your CIF

You should attempt to resolve as many as possible of the alerts in all categories. Often the minor alerts point to easily fixed oversights, errors and omissions in your CIF or refinement strategy, so attention to these fine details can be worthwhile. In order to resolve some of the more serious problems it may be necessary to carry out additional measurements or structure refinements. However, the nature of your study may justify the reported deviations from journal submission requirements and the more serious of these should be commented upon in the discussion or experimental section of a paper or in the "special_details" fields of the CIF. *checkCIF* was carefully designed to identify outliers and unusual parameters, but every test has its limitations and alerts that are not important in a particular case may appear. Conversely, the absence of alerts does not guarantee there are no aspects of the results needing attention. It is up to the individual to critically assess their own results and, if necessary, seek expert advice.

If you wish to submit your CIF for publication in Acta Crystallographica Section C or E, you should upload your CIF via the web. If you wish to submit your CIF for publication in IUCrData you should upload your CIF via the web. If your CIF is to form part of a submission to another IUCr journal, you will be asked, either during electronic submission or by the Co-editor handling your paper, to upload your CIF via our web site.

PLATON version of 19/02/2022; check.def file version of 19/01/2022

Datablock 1a - ellipsoid plot

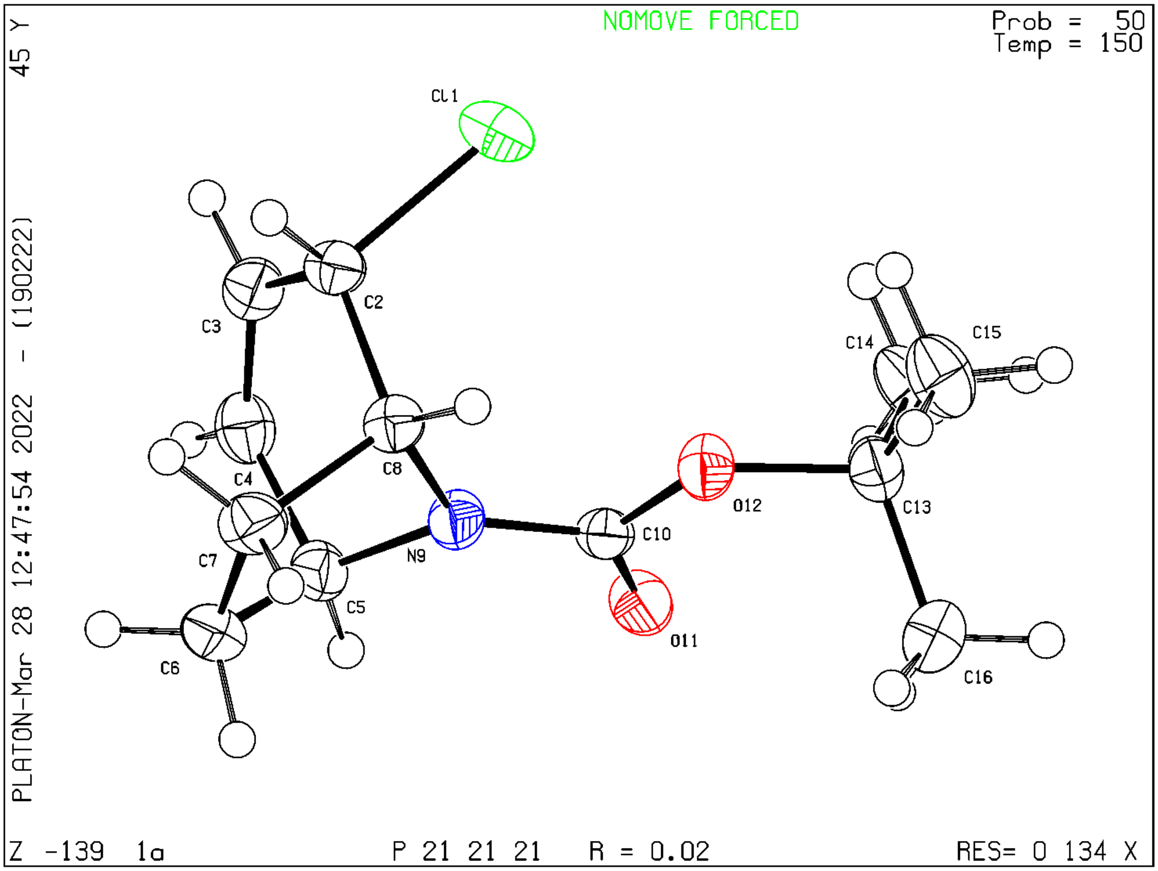

Datablock 3p - ellipsoid plot

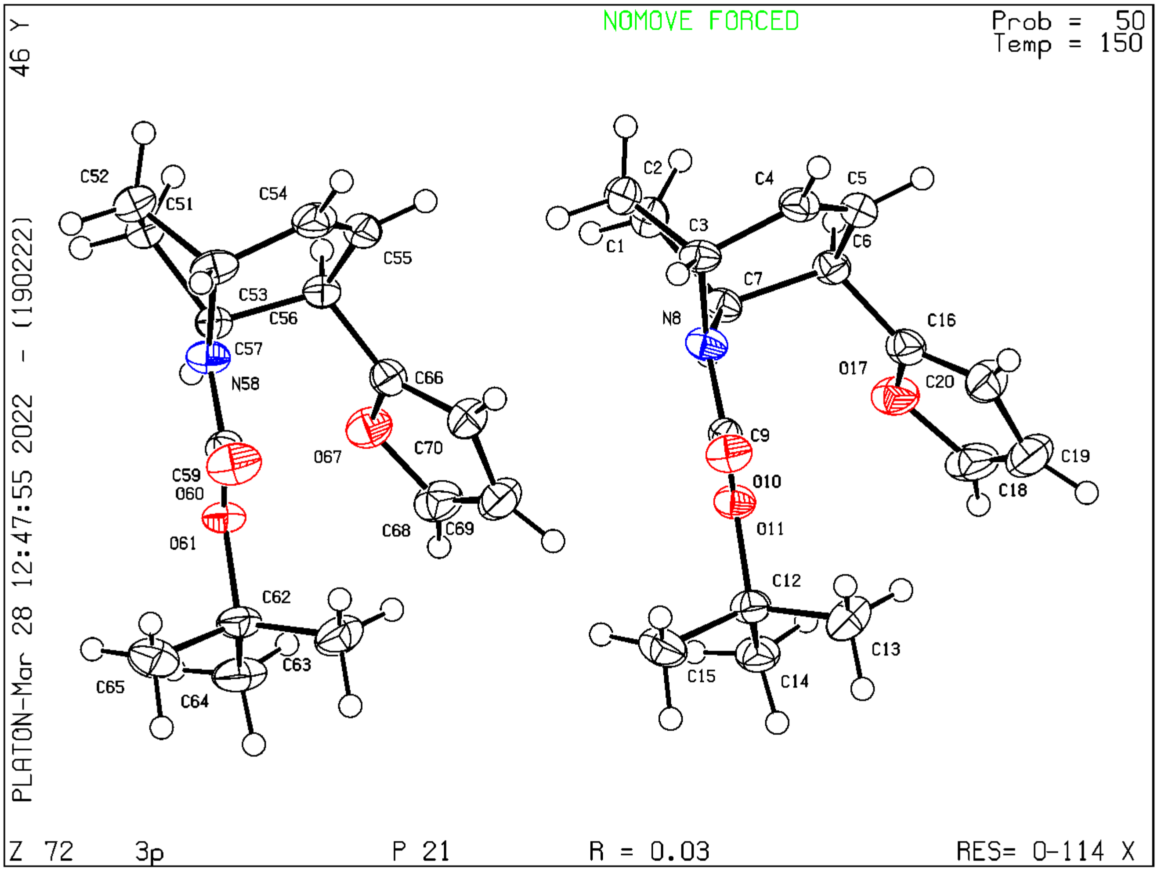

Datablock 6 - ellipsoid plot

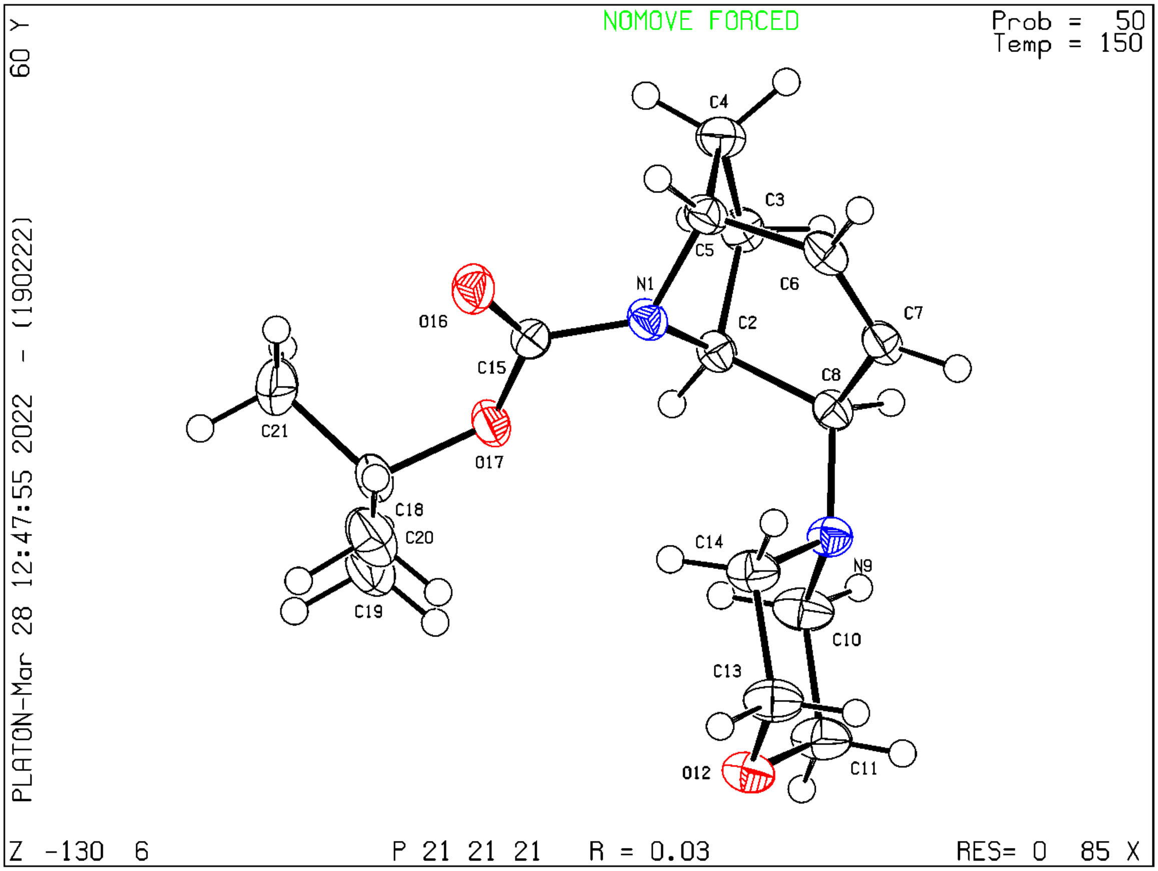

Supplement: Supplementary file 3 — cs2c02259_si_003.pdf [file cs2c02259_si_003.pdf]
